# Enumeration of Autocatalytic Subsystems in Large Chemical Reaction Networks

**DOI:** 10.1021/acs.jctc.5c01979

**Published:** 2026-05-04

**Authors:** Richard Golnik, Thomas Gatter, Peter F. Stadler, Nicola Vassena

**Affiliations:** † Bioinformatics Group, Department of Computer Science, Leipzig University, D-04107 Leipzig, Germany; ‡ Interdisciplinary Center for Bioinformatics, Leipzig University, D-04107 Leipzig, Germany; § Zuse School for Embedded and Composite Artificial Intelligence (SECAI), D-04107 Leipzig, Germany; ∥ Center for Scalable Data Analytics and Artificial Intelligence, Leipzig University, D-04107 Leipzig, Germany; ⊥ Max Planck Institute for Mathematics in the Sciences, D-04103 Leipzig, Germany; # Department of Theoretical Chemistry, University of Vienna, A-1090 Wien, Austria; ¶ Center for Non-coding RNA in Technology and Health, University of Copenhagen, DK-1870 Frederiksberg, Denmark; ∇ Facultad de Ciencias, Universidad Nacional de Colombia, 111111 Bogotá, Colombia; ○ Santa Fe Institute, Santa Fe, New Mexico 87501, United States

## Abstract

Autocatalysis is an important feature of metabolic networks, contributing crucially to the self-maintenance of organisms. Autocatalytic subsystems of chemical reaction networks (CRNs) are characterized in terms of algebraic conditions on submatrices of the stoichiometric matrix **S**. Here, we derive sufficient conditions for subgraphs supporting irreducible autocatalytic systems in the bipartite König representation of the CRN. On this basis, we develop an efficient algorithm to enumerate autocatalytic subnetworks and, as a special case, autocatalytic cores, i.e., minimal autocatalytic subnetworks, in full-size metabolic networks. The same algorithmic approach can also be used to determine autocatalytic cores only. As a showcase application, we provide a complete analysis of autocatalysis in the core metabolism of *E. coli* and enumerate irreducible autocatalytic subsystems of limited size in full-fledged metabolic networks of *E. coli*, human erythrocytes, and *Methanosarcina barkeri* (Archaea). The mathematical and algorithmic results are accompanied by software enabling the routine analysis of autocatalysis in large CRNs.

## Graphical abstract

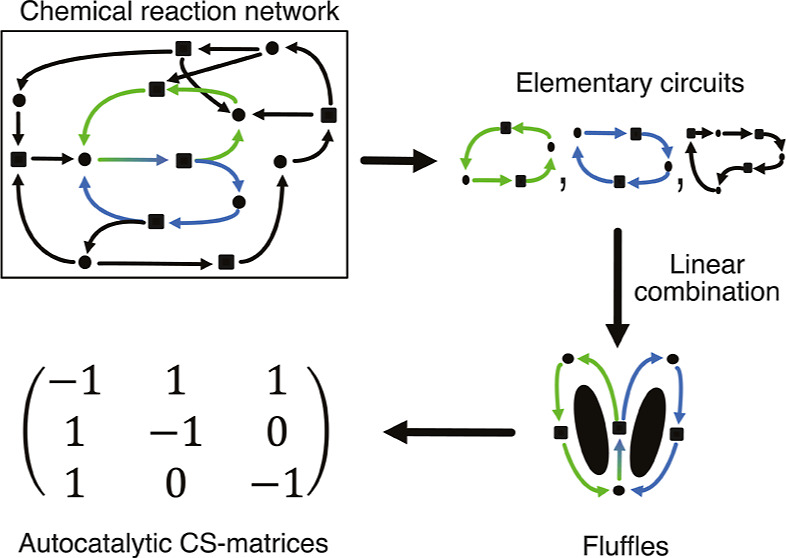

## Introduction

An autocatalytic reaction is “a chemical reaction in which a product (or a reaction intermediate) also functions as a catalyst”.^1^ Self-replication, i.e., the ability of multiplying instances of the self, is a special case of autocatalysis that is inherent to all living organisms. The emergence of self-replicating systems hence is a key issue in theories of the origin of life, independent of whether an RNA world, a lipid world, or a metabolism-first scenario is envisioned.^2–8^ In a more general setting, autocatalysis is a property of chemical reaction networks (CRNs) that collectively implements an autocatalytic overall reaction without any of the constituent reactions being autocatalytic. It is important to distinguish two fundamentally different modeling frameworks: networks of autocatalysts, such as the hypercycles of Eigen & Schuster,^3^ and catalytic reaction systems of Hordijk & Steel,^9^ which presuppose that all reactions are explicitly and specifically catalyzed by members of the system. Such systems naturally represent interactions of complex entities such as RNAs, proteins, or other heteropolymers, even though there is mounting evidence that small molecules and cofactors in metabolic networks, as well as metal ions, also exert catalytic activity.^10^ In contrast, catalysis is usually considered as an emergent property in networks of abiotic chemical reactions among small molecules. More precisely, catalysis in this setting is the net effect of a sequence of individual reactions. Here, we will be concerned exclusively with CRNs without explicit catalysts.

Autocatalysis in CRNs was generally considered to be scarce in nonenzymatic chemistry.^11–13^ On the other hand, it has been argued repeatedly, that metabolic networks are dominated by autocatalytic subsystems.^9,12,14^ Until recently, the lack of a consistent definition of autocatalysis made it difficult to discuss the prevalence of autocatalytic structures in chemical networks.^15^ This situation changed when Blokhuis et al. proposed an algebraic definition of autocatalytic submatrices based only on structural properties of a chemical network encoded by the stoichiometric matrix.^16^ This notion of autocatalysis has become widely accepted because it not only captures key features of collective autocatalysis, but also turned out to mathematically well-behave^17^ and to be suitable for constructing practical algorithms^18^ identifying autocatalytic subnetworks. To the best of our knowledge, available tools enumerating autocatalytic cycles are restricted to network sizes of approximately 300 metabolites and reactions.^18–21^ Gagrani et al.^18^ at present offers the most capable method currently accessible for networks of this scale, while the other approaches are either not yet publicly available^19^ or limited to smaller networks.^20^ This falls short of the capability to analyze much larger metabolic networks in living organisms ranging from bacteria to animals and plants.^22–28^

## Road Map

In this contribution, we describe a graph-theoretical approach to identify irreducible autocatalytic subsystems and the software package autogato that implements these methods. The concise and self-contained presentation of the mathematical results underlying the algorithmic developments requires a considerable level of technicality. We therefore start with a roadmap of this contribution that summarizes the main results and their implications in a less technical manner and with only a minimum of notation. Still, we introduce a few concepts formally already in this overview section. An illustration of this section is depicted in Figure 1.

**Figure 1:**
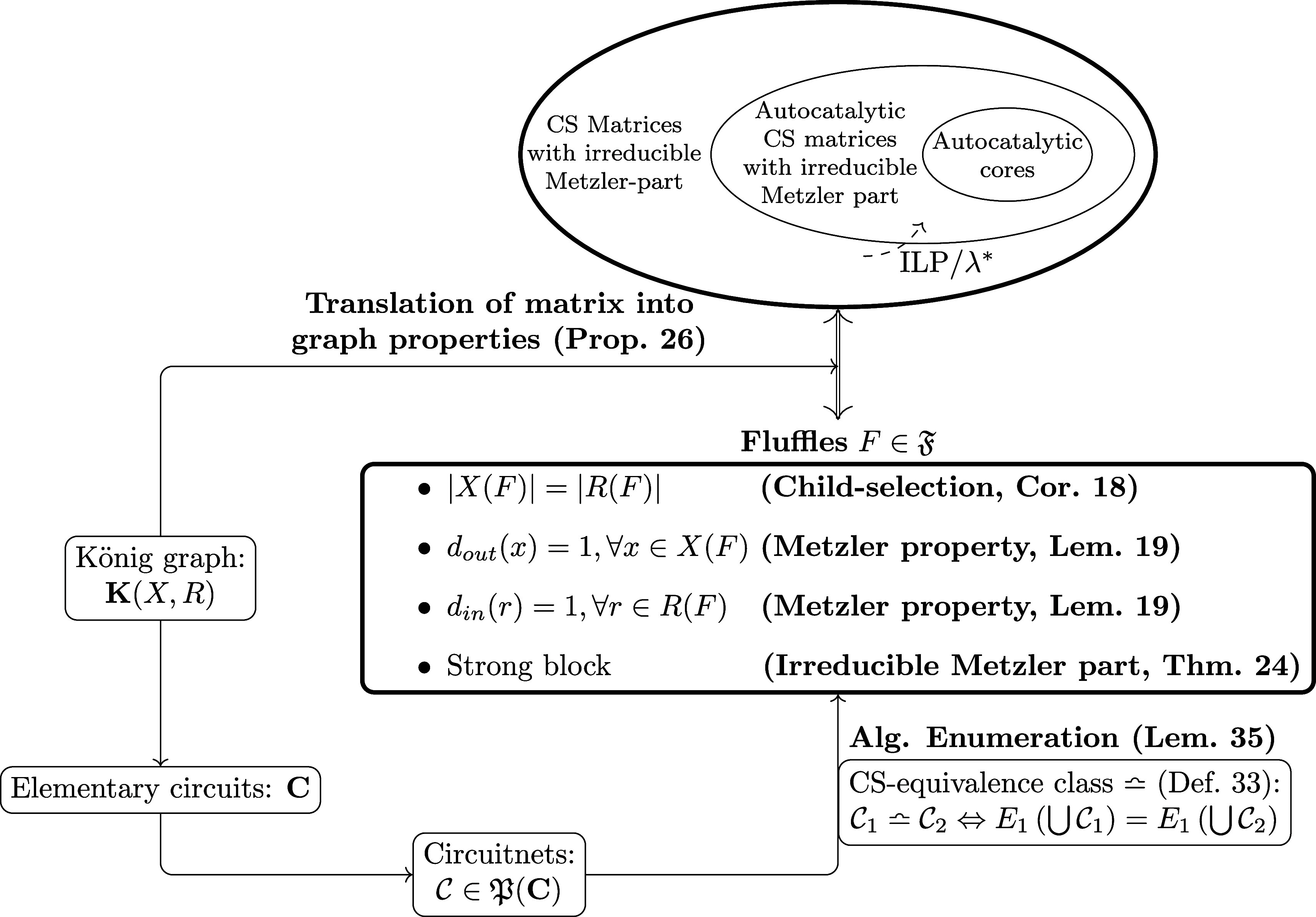
Schematic depiction of the main results. A CRN Γ = (*X*, *R*) is represented by its bipartite König graph **K**(*X*, *R*). Child-selection (CS) matrices with irreducible Metzler part can be identified as subgraphs of **K**, termed fluffles. Equivalent fluffles, w.r.t. to their CS matrices, are enumerated efficiently via sets of elementary circuits (circuitnets) by taking advantage of CS-equivalence classes. CS matrices **S**[**κ**] of fluffles are extracted and their autocatalytic capacity assessed using an ILP or, in the Metzler case, computing the leading eigenvalue λ* (Lemma 11). The matrix properties inducing fluffle properties and the relevant associated results are stated in brackets.

### Reaction Networks

A chemical reaction network (CRN) Γ is a pair of finite sets Γ ≔ (*X*, *R*), where *X* is a set of chemical species or metabolites and *R* is a set of chemical reactions. A reaction *r* is a directed transformation between nonnegative linear combinations of metabolites and can be described as

1 $$\sum_{x\in X}s_{xr}^{-}\cdot x\underset{r}{\to }\sum_{x\in X}s_{xr}^{+}\cdot x$$

with *s*_*xr*_^–^ ≥ 0 and *s*_*xr*_^+^ ≥ 0 denoting the nonnegative stoichiometric coefficients of the molecular count. Typically these coefficients are integers, but our theory does not make a distinction about it. Metabolites *x* appearing with nonzero *s*_*xr*_^–^ > 0 on the left-hand side of ([Disp-formula eq1]) are called reactants or substrates or educts of *r*, while metabolites *x* appearing with nonzero *s*_*xr*_^+^ > 0 on the right-hand side of ([Disp-formula eq1]) are called products of *r*. We may collect the stoichiometric coefficients of the reactant species in the |*X*| × |*R*| reactant matrix **S** ^–^ : **S**_*xr*_^–^≔ *s*_*xr*_^–^ and the coefficients of the product species in the |*X*| × |*R*| product matrix **S** ^+^ : **S**_*xr*_^+^≔ *s*_*xr*_^+^. The difference between the two matrices gives rise to the stoichiometric matrix **S**≔ **S** ^+^ – **S** ^–^ with entries

2 $$S_{xr}≔s_{xr}^{+}-s_{xr}^{-}$$

In the absence of explicit catalysis, no chemical species *x* is both reactant and product of the same reaction *r* and hence **S**_*xr*_ > 0 implies **S**_*xr*_ = *s*_*xr*_^+^ and *s*_*xr*_^–^ = 0, i.e., *x* is a product of *r*, while **S**_*xr*_ < 0 implies **S**_*xr*_ = −*s*_*xr*_^–^ and *s*_*xr*_^+^ = 0, i.e., *x* is a reactant of *r*. The stoichiometric matrix then completely describes the CRN. In particular, therefore, we do not consider here explicitly autocatalytic reactions of the form $s_{xr}^{-}\cdot x+...\underset{r}{\to }s_{xr}^{+}\cdot x+...$ with 0 < *s*_*xr*_^–^ < *s*_*xr*_^+^.^3,7^ Throughout this contribution, we assume that there are no explicit catalysts. An extension to the more general case will be considered elsewhere.^29^

The most natural representation of Γ is a directed, weighted hypergraph with vertex set *X* and edge set *R*. Each reaction *r* ∈ *R* is represented by a hyperarrow, whose inputs correspond to the reactant species and outputs to the product species. The stoichiometric coefficients appear as weights for the hyperarrow. Building on this perspective, we consider CRNs in König representation^30^ throughout, i.e., we represent the hypergraph Γ as a directed bipartite graph

3 K≔K(X,R)≔(X∪̇R,E)

with disjoint vertex sets *X* and *R* and edge set *E* ≔ *E*_1_ ∪ *E*_2_ where $E_{1}\left(K\right)≔\left\{\left(x,r\right)|s_{xr}^{-}>0\right\}$ and $E_{2}\left(K\right)≔\left\{\left(r,x\right)|s_{xr}^{+}>0\right\}$ . The absence of explicit catalysts excludes so-called digons, i.e., pairs of edges (*x*, *r*) and (*r*, *x*) are never present in **K**.

### Child-Selections (CS)

Child-selections (CS)^17,31^ are a central technical tool relating the structure of a CRN to certain dynamical properties. Mathematically, they are motivated by a structural and symbolic analysis of the Jacobian matrix of dynamical systems associated with a CRN. More precisely, via a Cauchy–Binet decomposition, only the square “CS” matrices **S**[**κ**] associated with a child-selection contribute to the characteristic polynomial of the Jacobian matrix. We briefly introduce them below, but we defer a detailed discussion of this topic to Section Parameter-Rich Chemical Kinetics.

**Definition 1**. A *k*-child-selection triple, or *k*-CS for short, is a triple **κ** = (*X*_κ_, *R*_κ_, κ) such that |*X*_κ_| = |*R*_κ_| = *k, X*_κ_ ⊆ *X, R*_κ_ ⊆ *R*, and κ: *X*_κ_ → *R*_κ_ is a bijection satisfying *s*_*x*κ(*x*)_^–^ > 0 for all *x* ∈ *X*_κ_. *We call* κ *a* CS bijection.

To any given *k*-CS **κ** = (*X*_κ_, *R*_κ_, κ), we associate a *k* × *k* CS matrix **S**[**κ**] defined as follows

4 $$S{\left[\kappa \right]}_{xw}≔s_{x\kappa \left(w\right)}^{+}-s_{x\kappa \left(w\right)}^{-},,\text{for}\;\text{all}\;x,w\in X_{\kappa }$$

Note that a CS matrix may differ from a submatrix of **S** by having a different column order. In particular, for a fixed ordering of the species *X* = {*x*_1_, ..., *x*_|*X*|_}, the column order of the stoichiometric matrix **S** depends on the ordering of the set *R*, whereas the ordering of **S**[**κ**] is independent of it, depending only on the order of *X*.

### Autocatalysis

Blokhuis et al.^16^ derived a matrix definition of autocatalysis from the definition of the IUPAC (International Union of Pure and Applied Chemistry):^1^

**Definition 2**. *A matrix* $A\in R^{n,m}$ is autocatalytic if

(i) there is $v\in R_{>0}^{m}$ such that **A** *v* > 0;
(ii) for each *r* ∈ {1, ..., *m*} there is *x*, *y* ∈ {1, ..., *n*}: **A**_*xr*_ < 0 and **A**_*yr*_ > 0.

We use **S**[*X*_κ_, *R*_κ_] to refer to the submatrix of **S** with species index in *X*_κ_ ⊆ *X* and reaction index in *R*_κ_ ⊆ *R*, and we can then define an autocatalytic network consequently.

**Definition 3**. A network Γ is autocatalytic if its stoichiometric matrix **S** possesses an autocatalytic submatrix.

Recent literature on autocatalysis^16–18^ put considerable emphasis on minimal autocatalytic matrices, so-called autocatalytic cores:

**Definition 4**. A matrix **A** is an autocatalytic core if **A** is autocatalytic and does not contain a proper autocatalytic submatrix.

The main goal of this contribution is to develop an algorithmic approach to compute autocatalytic substructures in large CRN.

### From Autocatalytic Matrices to Fluffle Graphs

The key idea underlying our approach is to exploit relationships between certain autocatalytic subnetworks (*X*′, *R*′), including autocatalytic cores, and subgraphs of **K** with special structures. This is not a trivial endeavor since the conditions on submatrices of **S** that define autocatalysis are algebraic by nature and do not have an obvious translation to the language of graphs.

Our starting point is the known observation that each autocatalytic core induces a unique child-selection **κ** since each reaction *r* in a core has a unique reactant, which appears as a negative entry in the diagonal of **A** = **S**[**κ**], see^17^ and Prop. 9 below. Child-selections, in turn, yield the first tangible connection to graphs: each pair (*x*, κ­(*x*)) appears as an edge in **K** that connects a reaction with one of its reactants. Each child-selection **κ** = (*X*′, *R*′, κ), therefore, can be associated with a subgraph **K**(**κ**) that is spanned by the set of reactant-to-reaction edges *E*_1_(**κ**) ≔ {(*x*, κ­(*x*)) | *x* ∈ *X*′} (see Thm. 16). Our task thus becomes the characterization of child-selections, and thus subgraphs of **K**, which can give rise to interesting autocatalytic subnetworks. To this end, we further investigate the matrices **S**[**κ**].

The second key observation is that for autocatalytic cores, **S**[**κ**] is a so-called Metzler matrix, i.e., all off-diagonal entries are non-negative, see ref 16,17 and Prop. 8 below, which implies that **K**(**κ**) is an induced subgraph of **K** (Cor. 41). For more general child-selections, we show that a necessary condition for **S**[**κ**] to be autocatalytic is that its Metzler part 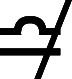 (S[κ]) is autocatalytic. This matrix is obtained by replacing all negative off-diagonal entries in **S**[**κ**] by 0. The matrix 
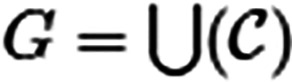
 (S[κ]) in turn corresponds exactly to the (not necessarily induced) subgraph **K**(**κ**).

Autocatalytic cores are not the only interesting autocatalytic subnetworks. In child-selections, every species *x* is consumed; if it is not produced, it cannot be part of an autocatalytic subnetwork. The definition of autocatalysis, furthermore, requires that every reaction has at least one reactant and at least one product. Thus, every species *x* ∈ *X*′ and every reaction *r* ∈ *R*′ of an autocatalytic CS **κ** = (*X*′, *R*′, κ) lies on a directed circuit in **K**, and equivalently, all *x* ∈ *X*′ lie on a circuit in the so-called substrate graph, which coincides with the graph representing the non-negative matrix 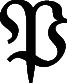 (S[κ]) . Very naturally, we restrict ourselves to irreducible matrices 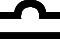 (S[κ]) and thus strongly connected substrate graphs. Considering the corresponding subgraph **K**(**κ**), we show that the irreducible matrices 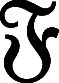 (S[κ]) are in 1–1 correspondence to subgraphs of **K**(**κ**) with the following properties:

(i) **K**(**κ**) is balanced, i.e., |*X*′| = |*R*′|, Cor. 18;
(ii) every *x* ∈ *X*′ has out-degree 1 and every *r* ∈ *R*′ has in-degree 1, Lem. 19;
(iii) **K**(**κ**) is a strong block, i.e., it is strongly connected and its underlying undirected graph is biconnected, Thm. 24.

We introduce the term fluffle for directed bipartite (sub)­graphs satisfying (i), (ii), and (iii).

In the following, we say that an (autocatalytic) subnetwork defined by a CS **κ** is irreducible if the Metzler part of its corresponding child-selection matrix, i.e., 

 (S[κ]) , is irreducible.

### From Fluffles to Enumerating Irreducible Autocatalytic Subnetworks

The discussion so far suggests an attractive avenue to list irreducible autocatalytic subnetworks: enumerate the fluffle subgraphs of **K** and test their corresponding CS matrices. Our task, therefore, becomes to list fluffles efficiently. The key property of fluffles that makes such an algorithm practicable is the fact that fluffles are characterized by a special class of ear decompositions (Thm. 27). More precisely, every elementary circuit is a fluffle, and a directed ear can be added to a fluffle if and only if it has an even number of inner vertices, its initial vertex is a reaction vertex, and its terminal vertex is a species vertex. This yields a simple condition on how a fluffle and an additional elementary circuit may overlap for the superposition to again be a fluffle, and suggests the notion of circuitnets as sets of compatible elementary circuits that produce fluffles (Thm. 31). The final building block is the observation that for every child-selection **κ** there is a representative (maximal) fluffle that can be constructed from elementary circuits as follows: *E*_1_(**κ**) = 
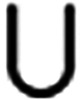
_*i*_ *E*_1_(*C*_*i*_), i.e., the union of the reactant-to-reaction edges of the superimposed elementary circuits defines the CS edges, and thus *X*′ and *R*′. The remaining (reaction-to-product) edges are then induced by **K**, i.e., *E*_2_ = *E*(**K**) ∩ (*R*′ × *X*′), see Prop. 38. Moreover, the *E*_2_ edges (κ­(*x*), *y*) correspond to positive off-diagonal entries 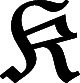 ${\left(S\left[\kappa \right]\right)}_{xy}>0$ . In particular, therefore, we need to consider only one elementary circuit for *X*′ ∪ *R*′ and restrict circuitnets to “fundamental” ones, where each circuit covers vertices not present in any other one.

### Computational Results and Performance

These mathematical results form the basis for a practical strategy to enumerate irreducible autocatalytic subnetworks, or more precisely, autocatalytic CS matrices with an irreducible Metzler part from the König graph **K** of a CRN. Conceptually, we can break up this task into four steps:

(1) Enumerating the elementary circuits of **K**.
(2) Construction of representatives for “CS-equivalence classes” (see Def. 33 and 37) of fluffles by iteratively adding elementary circuits.
(3) Testing of these candidate CS matrices **S**[**κ**] for autocatalysis.
(4) Identification of autocatalytic cores.

The algorithms addressing these basic tasks are described in detail in Section Basic Algorithms. The enumeration of elementary circuits in digraphs is a well-studied task for which we employ Johnson’s algorithm^32^ in a version that optionally limits the length of the circuit.^33^ The algorithm described in this contribution is implemented in the python package autogato (https://github.com/hollyritch/autogato), which is distributed with a detailed description and examples. We therefore refrain from a more thorough description of the software here.

The direct application of this strategy to large CRNs, in particular to genome-scale metabolic networks, requires prohibitive computational resources. We observe, however, that the König graph of metabolic networks is rather sparsely connected. Moreover, it typically contains modules with higher internal connectivity that often can be identified with functional biological submodules.^34–38^ We use this structure to decompose the CRN into smaller parts following a divide-and-conquer approach. Interfaces between submodules, however, may also be part of autocatalytic subsystems. We therefore consider elementary circuits that connect modules in the final stage. A major advantage of the decomposition into modules is that their analysis can be trivially parallelized, making it possible in practice to tackle large metabolic networks.

The exhaustive enumeration of all autocatalytic subnetworks with irreducible Metzler part or all autocatalytic cores is only feasible for moderate-sized CRNs, such as a model of the core carbon metabolism with 36 metabolites and 71 reactions obtained by removing exchange metabolites from the model proposed earlier.^39^ Nevertheless, our approach makes it feasible to tackle much larger networks by restricting the size of subnetworks of interest. For genome-scale metabolic networks (again, after removing a small set of exchange metabolites) autogato can enumerate all irreducible autocatalytic subnetworks (including all cores) up to sizes of 10 metabolites and 10 reactions (e.g., for *E. coli* DH5α).

The computational results in Section Showcase Applications indicate that autocatalysis is prevalent in all domains of life, however, to varying degrees. In particular, they support earlier claims that autocatalytic subsystems are abundant in metabolic networks.^9,12,14^ Moreover, they demonstrate that the graph-theoretic approach is robustly applicable to metabolic network models of practical interest and that autogato is a tool that can be used routinely by system biologists and chemists.

### The Remainder of This Contribution

The remainder of this contribution is concerned with the technical details required to make the roadmap above precise. We start in Section Reaction Kinetics, Child-selections, and Autocatalysis with a summary of the main results on autocatalytic sets and the—closely related—connection between child-selections, the stoichiometric matrix, and the reaction kinetics of a CRN. In Section Autocatalytic König graphs, we characterize a class of subgraphs of the bipartite König representation of a CRN that we term fluffles and show that only fluffles can induce autocatalytic subsystems with irreducible Metzler part in the CRN. The graph-theoretical algorithm enumerating the autocatalytic subsystems in a CRN is described in full detail in Section Algorithms. In these two sections, we introduce all necessary notation and report our mathematical results in the form of precise statements. Since this material is already quite extensive, we relegate all lengthy and technical proofs to the Supporting Information.

Subsequently, Section Showcase applications provides details on the selected computational results briefly discussed above. Although the technical part of this paper provides a fairly comprehensive understanding of the connection between autocalysis and the König graph of a CRN, some technical questions of interest remain open; they are briefly summarized in the Discussion. A short appendix connects the formalism derived here—focused on circuits in the König graph **K**—with the classification of autocatalyic cores by Blokhuis et al.^16^ To this end, we introduce the notion of centralized autocatalysis. Finally, we clarify the relationship of autocatalytic core and minimal autocatalytic subsystems (MAS) introduced by Gagrani et al.,^18^ and show that our algorithmic approach can also enumerate MAS.

## Reaction Kinetics, Child-Selections, and Autocatalysis

### Parameter-Rich Chemical Kinetics

Let $z\left(t\right)\in R_{\geq 0}^{\left|X\right|}$ indicate the vector of the chemical concentration of the species at time *t* in a well-mixed, spatially homogeneous, reactor. The time-evolution for *z*(*t*) is described by the system of ordinary differential equations (ODEs)

5 ż=f(z)≔S·ν(z)

where **S** is the stoichiometric matrix defined in ([Disp-formula eq2]), and $\nu \left(z\right)\in R^{\left|R\right|}$ is the vector of the reaction rate functions (kinetics). A primary modeling issue in reaction networks is the ubiquitous lack of precise knowledge of the mathematical form of the rates ν­(*z*). For this reason, the literature typically resorts to kinetic models: a wide class of reaction functions defined as follows.

**Definition 5**. A monotone kinetic model for a reaction network Γ ≔ (*X*, *R*) is a vector-valued function $\nu :R_{\geq 0}^{\left|X\right|}\to R_{\geq 0}^{\left|R\right|}$ , which satisfies the following conditions:

i. ν_*r*_(*z*) ≥ 0, for all $z\in R_{\geq 0}^{\left|X\right|}$ ;
ii. ν_*r*_(*z*) > 0 implies *z*_*x*_ > 0 for all species x with *s*_*xr*_^–^ > 0;
iii. *s*_*xr*_^–^ = 0 implies ∂ν_r_/*∂z*_*x*_ ≡ 0;
iv. *z* > 0 and *s*_*xr*_^–^ > 0 implies ∂ν_*r*_/*z*_*z*_ > 0.

Since this work always focuses on monotone kinetic models, for brevity, throughout we simply write “kinetic models”. Given the aforementioned uncertainty on the precise quantities involved, it is typical to consider parametric kinetic models. Whenever necessary, we will refer to this dependency by writing ν­(*z*, *p*). Standard examples of such parametric kinetic models are classic^40^ and generalized^41^ mass-action kinetics, both polynomials, and more involved rational functions such as Michaelis–Menten kinetics^42^ and the Hill model.^43^

Consider now a network Γ endowed with a kinetic model ν. Fixed points *z̅* ≥ 0 of *f* in ([Disp-formula eq5]), i.e.

6 0=f(z̅)=S·ν(z̅)

are called steady-states of Γ. Throughout this work, we consider only consistent networks,^44^ whose stoichiometric matrix admits a positive right kernel vector **ν** > 0: **S** ·**ν** = 0, which is a necessary condition for a network Γ to admit at least one positive steady-state. It is well-known^45^ that the dynamical stability of *z̅* can be addressed at first approximation by studying the linearization of system (5) at *z̅*

7 $$ż=D_{f}\left(\mathrm{z̅}\right)z=\left(S\cdot N\left(\mathrm{z̃}\right)\right)z$$

where $D_{f}\left(\mathrm{z̅}\right)$ is the Jacobian matrix evaluated at *z̅*. The nonnegative matrix $N\in R^{\left|R\right|\times \left|X\right|}$ with entries

8 $$N_{rx}\left(\mathrm{z̅}\right)≔{\frac{\partial \nu_{r}\left(z\right)}{\partial z_{x}}|}_{z=\mathrm{z̅}}$$

is called reactivity matrix. In particular for hyperbolic steady-states *z̅*, i.e., for which the Jacobian $D_{f}\left(\mathrm{z̅}\right)$ has only eigenvalues with nonzero real part, the spectrum of the Jacobian determines the dynamical stability: the steady-state *z̅* is stable if $D_{f}\left(\mathrm{z̅}\right)$ is Hurwitz-stable, i.e. it possesses only eigenvalues with negative-real part, and *z̅* is unstable if $D_{f}\left(\mathrm{z̅}\right)$ is Hurwitz-unstable, i.e., it possesses at least one eigenvalue with positive-real part.

Given a network Γ endowed with a parametric kinetic model ν­(*z*, *p*), the relation between the network structure and the possible spectrum configurations of the Jacobian at the varying of parameters *p* is a classic problem that has turned out to be very challenging.^46^ These relationships become much more tractable if the parametric kinetic model has sufficient internal freedom, at least as long existence results are the major concern.^17^

**Definition 6**. A monotone kinetic rate model ν­(*z*, *p*) is parameter-rich if, for every positive steady-state *z̅* > 0 and every choice of an |*R*| × |*X*| matrix N satisfying $N_{rx}>0$ iff *s*_*xr*_^–^ > 0, there exists a choice of parameters p̅=p(z̅,N) such that $\partial \nu_{rx}\left(z,\mathrm{p̅}\right)/\partial z_{x}{|}_{z=\mathrm{z̅}}=N_{rx}$ .

Far from being just a theoretical construct, widely used schemes in biochemistry—such as Michaelis–Menten, Hill, and generalized mass action—are naturally parameter-rich. Classical mass-action kinetics, however, lacks sufficient parametric freedom and is therefore not parameter-rich.

The advantage of the parameter-rich framework is that we may then consider a symbolic reactivity matrix N , that is, any |*R*| × |*X*| matrix whose nonnegative symbolic entries satisfy $N_{rx}>0\Leftrightarrow s_{xr}^{-}>0$ , and study the spectrum of the associated *symbolic Jacobian matrix*

9 D≔SN

which no longer depends explicitly on the steady-state value *z̅*. In particular, under parameter-rich kinetics, the existence of some evaluation of N such that **D** has a given spectrum directly implies the existence of kinetic parameters for which this very **D** is realized as the actual Jacobian at a steady state *z̅*, and thus dynamical conclusions can be drawn. Accordingly, we say that the network Γ admits instability if there exists a choice N of the kinetic matrix such that the symbolic Jacobian **D** is Hurwitz-unstable.

On the other hand, since the entries of the symbolic reactivity matrix are determined as zero or positive solely from the stoichiometric matrix, this approach can be used to draw conclusions about the range of realizable dynamics from purely structural information. To see this, we consider a *k*-CS **κ**. Without loss of generality, assume *X*_κ_ = {*x*_1_, .., *x*_*k*_} ⊂ *X*. By choosing the following rescaling for the symbolic reactivity matrix N

10 $$N_{rx}\left(\varepsilon \right)=\{\begin{array}{ll} 1 & \text{if}\;x\in X_{\kappa }\text{and}r=\kappa \left(x\right)\\ \varepsilon & otherwise,\text{if}\;s_{xr}^{-}>0 \end{array}$$

a straightforward computation shows that the associated symbolic Jacobian **D**(ε) reads

11 $$D\left(\varepsilon \right)=\left(\begin{array}{ll} S\left[\kappa \right]+O\left(\varepsilon \right) & O\left(\varepsilon \right)\\ ... & O\left(\varepsilon \right) \end{array}\right)$$

where *O*(ε) indicate an expression of order ε. A detailed derivation can be found in Vassena and Stadler^17^ In particular, for ε small enough, the *k* eigenvalues of **S**[**κ**] approximate the *k* largest (in absolute value) eigenvalues of **D**(ε). This argument shows that any *k*-CS matrix can be used to approximate *k* dominant eigenvalues of the Jacobian. In particular, we obtain a straightforward condition for a network to admit instability:

**Proposition 7** (Cor. 5.1^17^). Consider a network Γ ≔ (*X*, *R*) with parameter-rich kinetics. If there is a *k*-CS **κ** such that its associated *k* × *k* CS matrix is Hurwitz-unstable, then the network admits instability.

Moreover, using the same line of reasoning, it was shown that the presence of autocatalysis in the network always implies that the network admits instability.^17^ The next section briefly reviews this connection.

### Autocatalytic Matrices

Since we exclude explicit catalysis here, we can equivalently express network properties as properties of the matrix **S**. Key features of autocatalytic cores are collected in the following proposition, which has been proven previously.^16,17^

**Proposition 8**. Let **Ã** be an autocatalytic core. The following all hold true:

1. ***A*** *~* is an invertible square matrix;
2. There exists a unique autocatalytic core **A** with strictly negative diagonal obtained by reordering the columns of **Ã**;
3. The off-diagonal entries of **A** obtained at point 2 are nonnegative.

Square matrices with nonnegative off-diagonal entries are called Metzler in the literature, and their stability properties have been extensively studied in connection with the Frobenius-Perron Theorem.^47^ Throughout this paper, we refer to autocatalytic cores **A** always intending the Metzler representation with negative diagonal and nonnegative off-diagonal. In this case, further properties—related to dynamical stability—were shown in ref 17.

**Proposition 9**. Let **A** be an *n* × *n* autocatalytic core in Metzler form. The following all hold true:

1. **A** = **S**[**κ**] for a unique child-selection **κ**;
2. **A** is irreducible;
3. **A** is Hurwitz-unstable. More precisely, **A** possesses exactly one eigenvalue with positive real part, and thus its determinant is of sign sign det **A** = (−1)^*n*−1^.

As a key consequence, these properties imply:

**Corollary 10**. If the network is autocatalytic, then it admits instability.

The emphasis on minimal autocatalytic subnetworks is mostly justified for qualitative and classification purposes. In contrast, the Jacobian rescaling ([Disp-formula eq11]) suggests that larger CS matrices may better capture the overall dynamical impact of autocatalysis on the system, in terms of instability and growth rate, since fewer variables are ε-rescaled. We therefore aim to develop a detection algorithm that goes beyond autocatalytic cores. A natural broader class of interest is given by CS matrices that are irreducible Metzler matrices. For this class, the link between autocatalysis and instability is fully preserved, as in Prop. 9. It can be restated as a direct consequence of the Perron–Frobenius theorem^17,47^ as follows:

**Lemma 11**. Let **S**[**κ**] be an irreducible Metzler matrix. The following are equivalent:

1. **S**[**κ**] is Hurwitz-unstable;
2. **S**[**κ**] has a real positive eigenvalue;
3. **S**[**κ**] is autocatalytic.

For irreducible Metzler matrices, Hurwitz-instability (a spectral property in general sensitive to column ordering) is therefore equivalent to autocatalysis (a structural property independent of ordering). On the other hand, without loss of generality in network labeling, any reducible Metzler CS matrix **S**[**κ**] can be represented in block form as

12 $$S\left[\kappa \right]=\left(\begin{array}{cc} S\left[\kappa^{\prime }\right] & 0\\ B & S\left[\kappa^{\prime \prime }\right] \end{array}\right)$$

where **S**[**κ**′] is irreducible. In the above representation, we say that **S**[**κ**] is decomposed as a cascade originating from **S**[**κ**′]. The next result shows that autocatalysis for **S**[**κ**] necessarily requires autocatalysis in **S**[**κ**′].

**Proposition 12** (Proof: Supporting Information). Let **κ** = (*X*_κ_, *R*_κ_, κ) be a k-CS whose associated CS matrix **S**[**κ**] is reducible, Metzler, and autocatalytic. Then there exists a *k*′-CS **κ**′ = (*X*_κ′_, *R*_κ′_, κ′) with *X*_κ′_ ⊂ *X*_κ_, *R*_κ′_ ⊂ *R*_κ_, and κ′(*X*_κ′_) = κ­(*X*_κ′_), such that its associated CS matrix **S**[**κ**′] is an irreducible autocatalytic Metzler matrix.

In line with our emphasis on Metzler matrices, we will see that the Metzler part of a CS matrix, introduced below, plays a key role.

**Definition 13**. For every CS matrix 

 **S**[**κ**] we define the *k* × k matrix (S[κ]) with entries
13

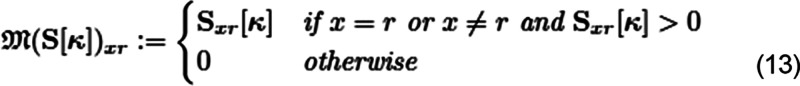




By construction, we have 

 ${\left(S\left[\kappa \right]\right)}_{xr}\neq S{\left[\kappa \right]}_{xr}$ if and only if *r* ≠ κ­(*x*) and **S**_*xr*_ < 0, i.e., if and only if *x* is a reactant of a reaction *r* other than the one assigned to *x* by the CS-bijection κ. These entries correspond exactly to the negative off-diagonal elements of **S**[**κ**]. Consequently, 

 (S[κ]) contains only nonnegative off-diagonal entries and is therefore a Metzler matrix. We therefore call 

 (S[κ]) the Metzler part of **S**[**κ**]. The Metzler part of a CS matrix will be of key interest because it yields a necessary condition for autocatalysis:

**Proposition 14**. If **S**[**κ**] is an autocatalytic CS matrix, then its Metzler part 

 (S[κ]) is autocatalytic.

Proof. We observe that 

 ${\left(S\left[\kappa \right]\right)}_{ij}\geq S{\left[\kappa \right]}_{ij}$ for all *i*, *j*, and hence 

 (S[κ])v≥S[κ]v holds for every nonnegative vector *v*.

In light of Prop. 12, any reducible autocatalytic CS matrix **S**[**κ**] can always be decomposed into a cascade originating from an irreducible autocatalytic Metzler matrix **S**[**κ**′]. Moreover, Prop. 14 thus justifies our focus on irreducible autocatalytic Metzler CS matrices.

□

## Autocatalytic König Graphs

### Child-Selective Subgraphs

We first turn our attention to identify substructures in the König graph that induce child-selections (CS). In the following, we will be concerned with subgraphs **K**′ of **K**. We write $V\left(K^{\prime }\right)=X\left(K^{\prime }\right)\cup ̇R\left(K^{\prime }\right)$ , and *E*(**K**′) = *E*_1_(**K**′) ∪ *E*_2_(**K**′) for the vertex and edge set of **K**′, respectively. Moreover, we denote the set of vertices incident with the edges in any edge set *E*_*i*_ by *V*(*E*_*i*_). Note that **K**′ is not necessarily an induced subgraph, i.e., *E*(**K**′) does not necessarily include all edges *e* ∈ **K** between two selected vertices *x*, *r* ∈ *V*(**K**′).

**Definition 15**. A subgraph **K**′ of **K** is child-selective if there exists a map κ: *X*(**K**′) → *R*(**K**′) such that **κ** = (*X*(**K**′), *R*(**K**′), κ) is a CS.

Since the map κ in a CS is bijective, **K**′ can only be child-selective if |*X*(**K**′)| = |*R*(**K**′)|. Recall that a matching in a graph is a set of vertex-disjoint edges, while a perfect matching is one incident with every vertex.

**Theorem 16**. A subgraph **K**′ ⊆ **K** is child-selective if and only if the subset $E_{1}\left(K^{\prime }\right)≔\left\{\left(x,r\right)|s_{xr}^{-}>0\right\}\subset E\left(K^{\prime }\right)$ of the reactant-to-reaction edges contains a perfect matching.

Proof. If **K**′ is child-selective with CS **κ**, we directly obtain a perfect matching by *E*_**κ**_ ≔ {(*x*, κ­(*x*)) | *x* ∈ *X*(**K**′)} ⊆ *E*_1_. Conversely, let *M* ⊆ *E*_1_(**K**′) ⊆ *E*_1_ be a perfect matching in **K**′, then there is (*x*, *y*) ∈ *M* for all *x* ∈ *X*(**K**′) and *y* is uniquely defined for every *x*. Thus, κ: *X*(**K**′) → *R*(**K**′) with κ­(*x*) = *y* if (*x*, *y*) ∈ *M* is uniquely defined and injective. Moreover, there is *u* with (*u*, *v*) ∈ *M* for all *v* ∈ *R*(**K**′). Hence κ is a bijection, and **κ** ≔ (*X*(**K**′), *R*(**K**′), κ) is a CS.

□

The proof of Thm. 16 contains an explicit recipe to construct CS. In fact, there is a 1–1 correspondence between perfect matchings in *E*_1_(**K**′) and bijections κ: *X*(**K**′) → *R*(**K**′). Moreover, the spanning subgraph **K**″ ⊆ **K**′ with *X*(**K**″) = *X*(**K**′), *R*(**K**″) = *R*(**K**′), and $E\left(K^{\prime \prime }\right)=M\cup ̇E_{2}\left(K^{\prime }\right)$ is child-selective for every perfect matching *M* ⊆ *E*_1_(**K**′). Conversely, for any CS **κ** = (*X*_κ_, *R*_κ_, κ), let *E*_1_^κ^ and *E*_2_^κ^ be subsets of *E*_1_ and *E*_2_, respectively, where edges have both adjacent vertices in $\left(X_{\kappa }\cup ̇R_{\kappa }\right)$ . Then we write

$$K\left(\kappa \right)≔\left(X_{\kappa }\cup R_{\kappa },M_{\kappa }\cup E_{2}^{\kappa }\right)$$ 14

defined by the perfect matching *M*_κ_ ⊆ *E*_1_^κ^. We note that **K**(**κ**) is a spanning subgraph of the induced subgraph **K**[**κ**] = **K**[*X*_κ_ ∪ *R*_κ_, *E*_1_^κ^ ∪ *E*_2_^κ^], see Figure 2 for an example. In the following subsections, we will exclusively investigate **K**(**κ**). We return to the induced subgraphs **K**[**κ**] in Section Metzler Matrices and Induced Fluffles only.

**Figure 2:**
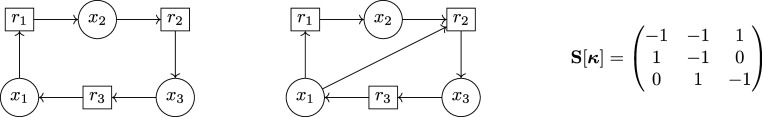
In general, **K**(**κ**) (left) is a proper subgraph of the induced subgraph **K**[**κ**] (middle). The example corresponds to the CS matrix **S**[**κ**] shown on the right with columns ordered as *x*_1_, *x*_2_, *x*_3_.

The subgraphs **K**(**κ**) naturally fit together with the Metzler matrices introduced in the previous section. Given a CS **κ** = (*X*′, *R*′, κ), we have a path (*x*, *r*, *y*) with *x*, *y* ∈ *X*′ and *r* ∈ *R*′ in **K**(**κ**) if and only if *r* = κ­(*x*) and *y* is a product of reaction *r*, i.e., if and only if **S**[**κ**]_*y*κ(*x*)_ > 0. Since we necessarily have *x* ≠ *y* in this case, and (*x*, *r*) is an edge in the child-selective subgraph if and only if *r* = κ(*x*), we observe that the nonzero off-diagonal entries of ​

 (S[κ]) uniquely determines the edge set *E*_2_(**K**(**κ**)), while κ, as we know, determines *E*_1_(**K**(**κ**)).

**Corollary 17**. For a child-selection **κ**, the subgraph **K**(**κ**) is the directed bipartite König graph determined by 

 (S[κ]) .

Moreover, each CS **κ**′ for the induced subgraph **K**[**κ**] gives rise to a distinct subgraph **K**(**κ**′), see Figure 3(left). We note in passing that polynomial-delay algorithms exist for enumerating perfect matchings in bipartite graphs.^48–50^ In the present work, however, we adopt a different approach to constructing the relevant child-selective subgraphs of **K**. The following statement is a direct consequence of the fact that κ is a bijection:

**Figure 3:**
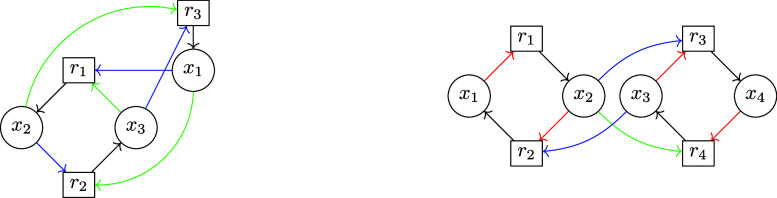
Left: Multiple CS may exist in a given induced subgraph of **K**. In the example, there are indeed two perfect matchings and thus two CS κ_1_ = (*X*_κ_, *R*_κ_, κ_1_) and **κ**_2_(*X*_κ_, *R*_κ_, κ_2_): κ_1_(*x*_1_) = *r*_1_, κ_1_(*x*_2_) = *r*_2_, κ_1_(*x*_3_) = *r*_3_ and κ_2_(*x*_1_) = *r*_2_, κ_2_(*x*_2_) = *r*_3_, κ_2_(*x*_3_) = *r*_1_. Right: A strongly connected child-selective subgraph may not have a CS matrix with irreducible Metzler part. Here, choosing, κ­(*x*_1_) = *r*_1_, κ­(*x*_2_) = *r*_2_, κ­(*x*_3_) = *r*_3_, and κ­(*x*_4_) = *r*_4_ (edges depicted in red) yields a block diagonal 

 (S[κ]) composed of two 2 × 2 blocks.

**Corollary 18**. Let **κ** be a CS. Then every substrate vertex *x* ∈ *X*_κ_ *in* **K**(**κ**) has out-degree 1 and every reaction vertex r ∈ *R*_κ_ *in* **K**(**κ**) has in-degree 1.

The subsequent results provide us with a purely graph-theoretical characterization of the subgraphs of **K** that derive from child selections.

**Lemma 19** (Proof: Supporting Information). Let $K^{\prime }=\left(X^{\prime }\cup ̇R^{\prime },E_{1}^{\prime }\cup ̇E_{2}^{\prime }\right)$ be a subgraph of **K** with reactant vertices *X*′, reaction vertices R′, and edges *E*_1_ ⊆ *X*′ × *R*′ and *E*_2_^′^ ⊆ *R* ^′^ × *X* ^′^ such that

1. |*X*′| = |*R*′|;
2. every *x* ∈ *X*′ has out-degree 1 and every *x* ∈ *R*′ has in-degree 1.

Then **K**′ is child-selective with κ­(*x*) = *r for* (*x*,*r*) ∈ *E*_1_^′^.

As an immediate consequence of Thm. 16 we note:

**Corollary 20**. Let **K** be an even elementary circuit graph. Then **K** is child-selective with a unique child-selection **κ**. Moreover, **K**(**κ**) = **K**.

**Corollary 21**. If **K** is not connected, then it is child-selective if and only if each weakly connected component is child-selective.

### Irreducibility and Strong Connectedness

We start this section with two simple technical observations:

**Lemma 22** (Proof: Supporting Information). Let **κ** = (*X*_κ_, *E*_κ_, κ) be a CS. Then **K**(**κ**) is strongly connected if and only if ​

 (S[κ]) is irreducible.

**Lemma 23** (Proof: Supporting Information). If **K**(**κ**) is strongly connected, then it does not contain a cut vertex.

Thus, if **K**(**κ**) is strongly connected, then its underlying undirected graph is also 2-connected. Such graphs are called strong blocks in the literature.^51^ Combining Lemmas 22 and 23 yields the main result of this section:

**Theorem 24**. Let **κ** be a CS. Then **K**(**κ**) is a strong block if and only if 

 (S[κ]) is irreducible.

The delicate interplay between the notions of child-selectiveness and strong blocks is exemplified in Figures 4 and 5. In particular, Figure 5b,c depict graphs that are strongly connected, albeit not strong blocks, but not child-selective. Figure 5d,e exemplify strong-blocks that are not child-selective.

**Figure 4:**
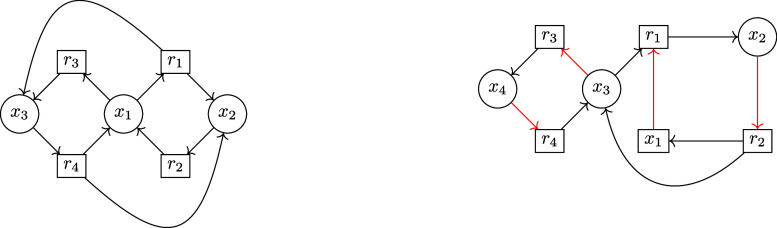
Left: Strongly connected bipartite graph without a cut-vertex but not child-selective, as the four reaction vertices are more than the three species vertices. Right: Strongly connected, child-selective (red edges) bipartite digraph with a cut-vertex.

**Figure 5:**
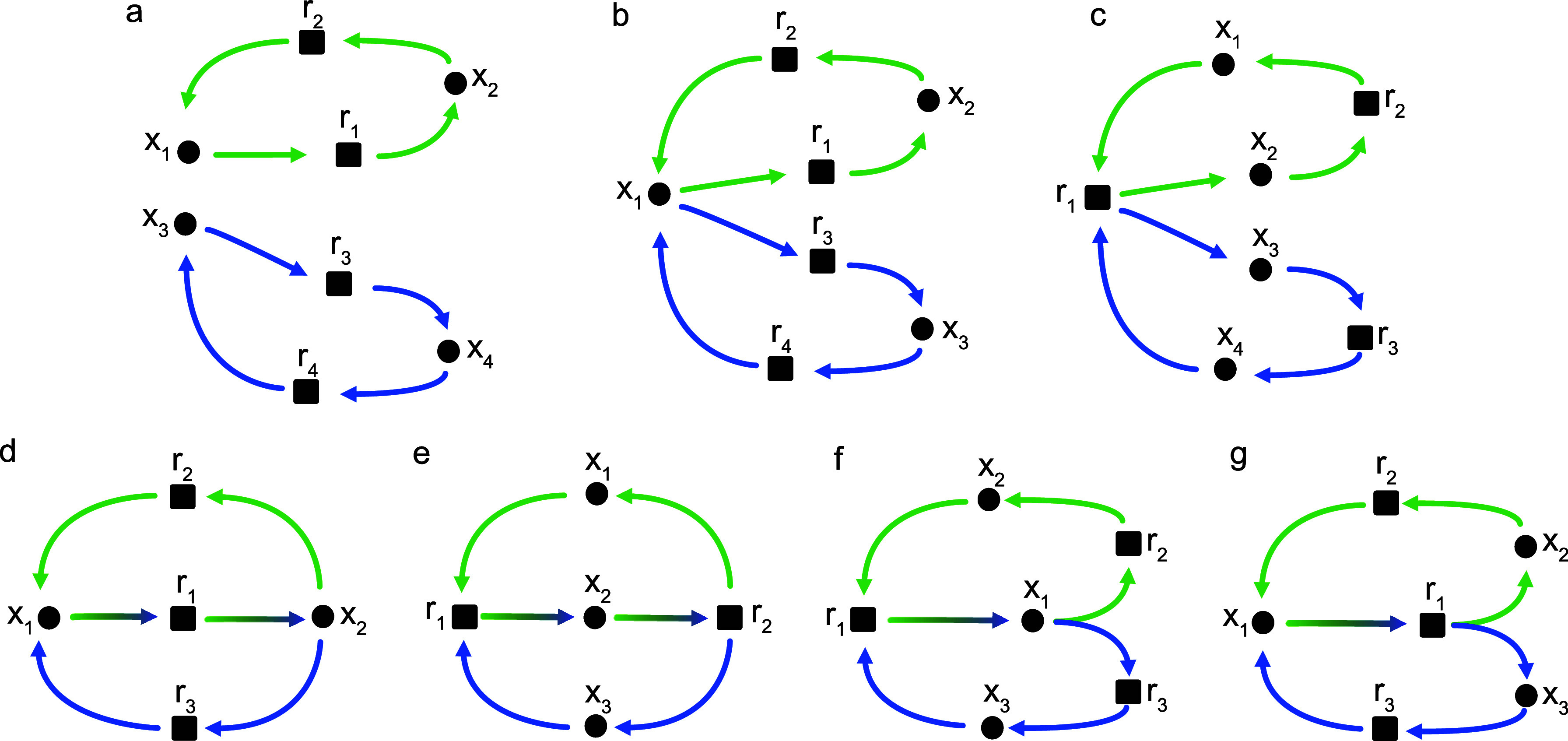
Unions of two elementary circuits, depicted in green and blue, yielding different configurations: (a) is not connected and thereby violates the fluffle condition (iii) in Prop. 26, (b–e) possess more metabolites than reaction vertices or vice versa, and contradict fluffle condition (i) in Prop. 26. In addition, (b,c) are also not a strong block and thus violate (iii) as well. The union of two elementary circuits as depicted in (f) has a substrate vertex *x*_1_ with out-degree two and a reaction vertex *r*_1_ with in-degree two, which contradicts fluffle condition (ii) in Prop. 26. The only combination consistent with the fluffle definition is depicted in (g), where indeed the intersection of the two elementary circuits is a path from a substrate to a reaction vertex, as prescribed by Thm. 29. The two elementary circuits in (d–f) moreover, also, constitute an example of circuitnets that are not associated with fluffles.

We conclude this section by revisiting the notion of autocatalysis and expressing Def. 2 in terms of the following graph-theoretic characterization of an autocatalytic CS. We recall the standard notions of source and sink vertices in a directed graph, i.e. vertices with no incoming edges (zero in-degree) and with no outgoing edges (zero out-degree), respectively.

**Lemma 25** (Proof: Supporting Information). A CS **κ** = (*X*_κ_, *E*_κ_, κ) is autocatalytic if and only if the following conditions both hold:

1. there is a positive vector *v* > 0 such that **S**[**κ**]*v* > 0.
2. **K**(**κ**) does not possess source and sink vertices;

### Fluffles and Circuitnets

Let us summarize the main arguments in the discussion so far. (1) By Prop. 14, we may restrict our attention to the Metzler parts of CS matrices. (2) By Prop. 12, an arbitrary autocatalytic CS matrix is either irreducible or it contains one or more disjoint irreducible autocatalytic blocks, so we may focus on CS matrices with irreducible Metzler part. (3) By Lemma 22 and Thm. 24, CS matrices with irreducible Metzler part correspond to strong blocks **K**(**κ**). (4) Using Cor. 18, Lemma 19, and Thm. 24, we finally derive the following central observation:

**Proposition 26**. Let G be a subgraph of **K**. Then there is a CS **κ** with a CS matrix that has an irreducible Metzler part 

 (S[κ]) such that *G* = **K**(**κ**) if and only if G satisfies

(i) G is bipartite with vertex partition V­(*G*) = *X*(*G*) ∪ *R*(*G*) such that |*X*(*G*)| = |*R*(*G*)|,
(ii) if *x* ∈ *X*(*G*) then x has out-degree 1 and if r ∈ *R*(*G*), then r has in-degree 1
(iii) *G* is a strongly connected block.

We call a graph satisfying these three properties a fluffle,[Fn fn1] and we denote such subgraphs with *G* throughout. Prop. 26 in particular implies that the enumeration of fluffle subgraphs in the König graph **K** encompasses all autocatalytic CS matrices with irreducible Metzler part. In the following, we describe how the fluffles can be constructed recursively.

Strongly connected blocks are precisely those graphs that admit an open directed ear decomposition,^51^ i.e., they can be constructed from an elementary circuit by iteratively adding open directed ears, which are directed paths whose end points are distinct vertices already present in the graph. Throughout the remainder of this contribution, we refer to open directed ear decompositions and open directed ears simply as “ear decompositions” and “ears”, respectively. After attaching an ear, the interior vertices of the ear have in-degree and out-degree 1, while its initial vertex has out-degree >1 and its terminal vertex has in-degree >1. In our bipartite setting, we then have the following theorem characterizing fluffle graphs.

**Theorem 27** (Proof: Supporting Information). A graph *G* is a fluffle if and only if it is bipartite with vertex set X ∪ R and it has an ear decomposition such that every ear initiates in a reaction vertex *r* ∈ *R* and terminates in a substrate vertex *x* ∈ *X*. In this case, all directed open ear decompositions have this property.

It will be useful to note that strong blocks in fluffles are again fluffles themselves:

**Lemma 28** (Proof: Supporting Information). Let G be a fluffle in **K** and *G*′ a subgraph of *G* that is a strong block. Then *G*′ is a fluffle.

Clearly, every elementary circuit in **K** is a fluffle. It is therefore natural to identify fluffles as unions of elementary circuits. According to Thm. 27, however, such unions must be consistent with an ear decomposition in which each ear originates at a reaction vertex and terminates at a substrate vertex. This requirement is made explicit in the following theorem. See also Figure 5 for an overview of possible ways to combine two elementary circuits, only one of which actually constitutes a fluffle.

**Theorem 29** (Proof: Supporting Information). Let G be a fluffle with vertex partition X ∪ R and C an elementary circuit such that Ø ⊂ *G* ∩ *C* ⊂ C. Then, the connected components of *G* ∩ *C* are directed paths P_i_. Moreover, *G* ∪ *C* is a fluffle if and only if all such paths P_i_ start from a substrate vertex *x*_*i*_ ∈ *X* and terminate with a reaction vertex *r*_*i*_ ∈ *R*.

**Definition 30**. A set $C=\left\{C_{1},...,C_{n}\right\}$ of elementary circuits in **K** is a circuitnet if there is an ordering π such that the union *G*_*k*_ ≔ 
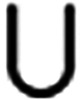
_*i*=1_^*k*^ *C*_*π*(*i*)_ is a strong block for all 1 ≤ *k* ≤ *n*.

We say that C is a circuitnet for a graph *G* if *G*, as a graph, is the union of all the elementary circuits in C , and we write 
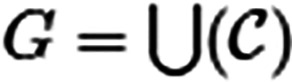
. The next theorem guarantees that there is a circuitnet for any fluffle *G*.

**Theorem 31**. Let G be a fluffle. Then there exists a circuitnet C for *G*, i.e.
15

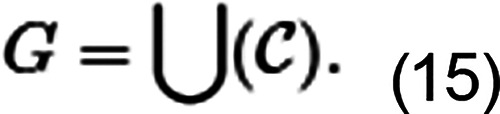




Proof. The statement follows directly from the well-known connection of elementary circuits, ear decompositions, and cycle bases: The cycle space of strongly connected digraphs has a circuit basis,^52^ and for strong blocks, such a basis can be constructed from an ear decomposition by completing each ear *P*_*i*_ to a directed circuit *C*_*i*_ using any directed path in *G*_*i*–1_ from the terminal to the initial vertex of the ear. In particular, therefore, every fluffle has a circuitnet.

□

Although there is at least one circuitnet for a fluffle *G*, in general, there may exist more circuitnets for the same fluffle. An example is depicted as the autocatalytic core of type V, (30): see Figure 6. A simple corollary follows from Thm. 31.

**Figure 6:**
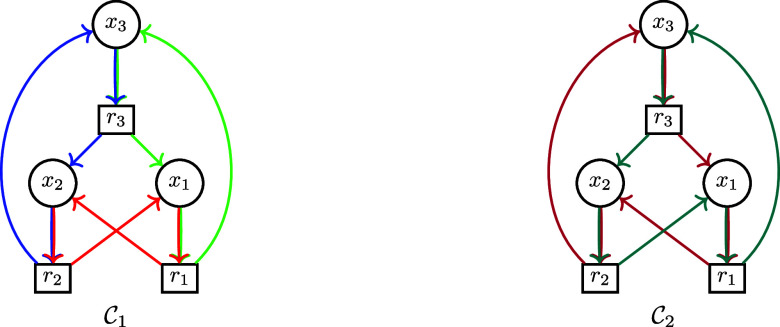
Fluffle corresponding to an autocatalytic core of Type V, eq 30. The fluffle *F* is depicted as a union of the elementary circuits in two different circuitnets. The circuitnet $C_{1}=\left\{C_{1},C_{2},C_{3}\right\}$ (left) comprises the three elementary circuits *C*_1_ = (*x*_1_, *r*_1_, *x*_3_, *r*_3_, *x*_1_) (green), *C*_2_ = (*x*_2_, *r*_2_, *x*_3_, *r*_3_, *x*_2_) (blue), and *C*_3_ = (*x*_1_, *r*_1_, *x*_2_, *r*_2_, *x*_1_) (red); the circuitnet $C_{2}=\left\{C_{4},C_{5}\right\}$ (right) consists of the two elementary circuits *C*_4_ = (*x*_1_, *r*_1_, *x*_3_, *r*_3_, *x*_2_, *r*_2_, *x*_1_) (teal) and *C*_5_ = (*x*_1_, *r*_1_, *x*_2_, *r*_2_, *x*_3_, *r*_3_, *x*_1_) (purple). We have F= 
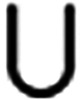
 $\left(C_{1}\right)=$ 
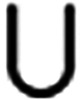
 $\left(C_{2}\right)$ .

**Corollary 32**. Let C be a circuitnet for the fluffle *G*. Then the following hold true.

1.
$C^{\prime }\subseteq C$ is a circuitnet for a fluffle *G*′ ⊆ *G* if and only if *G*′ is a strong block.
2. There exist an ordering π of the circuits in C such that *G*_*k*_ = 
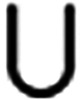
_*i*=1_^*k*^ *C*_*π*(*i*)_ is a fluffle.

Proof. The first statement follows directly from Lemma 28 and the second statement from the first and Def. 30.

□

### The Set of Fluffles of a CRN

Summarizing the discussion so far, we have shown that all CS matrices in Γ with an irreducible Metzler part, i.e., the viable candidates for “interesting” autocatalytic subnetworks, are the fluffles in the associated bipartite König graph **K** (Prop. 26). Moreover, any fluffle can be constructed by superimposing elementary circuits such that each intermediate step is itself a fluffle (Cor. 32). Equivalently, this boils down to enumerating the sets of circuitnets whose union is a fluffle. Elementary circuits can be enumerated efficiently in a lazy manner^32,53,54^ with linear delay, i.e., *O*(|*V*| + |*E*|). Moreover, algorithms exist that allow to restrict circuit length.^33^

Denote by 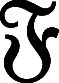 the set of circuitnets C whose union 
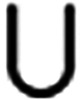
 (C) is a fluffle. In Supporting Information Section. The Set System of Circuitnets of Fluffles we summarize the properties of 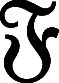 that enable efficient enumeration. The example in Figure 6 suggests to investigate the equivalence relation on 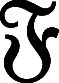 defined by $C_{1}∼C_{2}$ if 
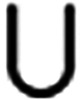
 $\left(C_{1}\right)=$ 
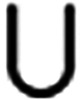
 $\left(C_{2}\right)$ . These equivalence classes are specified by subsets of edges in **K**. More precisely, we have $C_{1}∼C_{2}$ if and only if *E*(
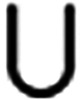
 $\left(C_{1}\right)$ ) = *E*(
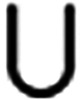
 $\left(C_{2}\right)$ ) because the corresponding vertex set is given implicitly by the vertices incident with these edge sets. Our primary aim, however, is not to enumerate fluffles, but to enumerate CS matrices **S**[**κ**] with irreducible Metzler parts. To this end, we recall that the CS **κ** is equivalent to the substrate-to-reaction edges in a fluffle, hence these edges completely determine **S**[**κ**]. This suggests to consider the following equivalence relation.

**Definition 33**. Let $C_{1}$ and $C_{2}$ be two circuitnets for fluffles *G*_1_ and *G*_2_. We say that $C_{1}$ and $C_{2}$ are CS-equivalent and we write $C_{1}$ 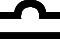 $C_{2}$ if
16

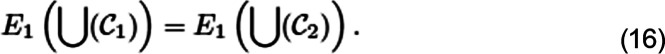




Let now κ_1_ = (*X*_**κ**_1__, *R*_**κ**_1__, κ_1_) and κ_2_ = (*X*_**κ**_2__, *R*_**κ**_2__, κ_2_) be two CS. We recall that we consider two CS matrices **S**[**κ**_1_] and **S**[**κ**_2_] are the same if

17 $$X_{\kappa_{1}}=X_{\kappa_{2}},,R_{\kappa_{1}}=R_{\kappa_{2}},,\kappa_{1}=\kappa_{2}$$

In other words, we consider two CS matrices the same if they involve the same species, reactions, and bijection between species and reactions. We do not require that the ordering of the rows is the same. That is, we consider **S**[**κ**_1_] and Π^*T*^ **S**[**κ**_2_]­Π to be same for any permutation matrix Π on *X*_**κ**_ × *X*_**κ**_. With this notion of same-ness, we obtain the following result.

**Lemma 34** (Proof: Supporting Information). Two circuitnets $C_{1}$ and $C_{2}$ for fluffles *G*_1_ and *G*_2_ yield the same CS matrix **S**[**κ**] if and only if $C_{1}$ 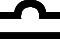 $C_{2}$ .

For example, consider the autocatalytic core of type V (30) depicted in Figure 6. The two circuitnets $C_{1}=\left\{C_{1},C_{2},C_{3}\right\}$ and $C_{2}=\left\{C_{4},C_{5}\right\}$ both are associated with the same fluffle and thus in particular they are CS-equivalent and give rise to the same CS matrix

18 $$S\left[\kappa \right]=\left(\begin{array}{ccc} -1 & 1 & 1\\ 1 & -1 & 1\\ 1 & 1 & -1 \end{array}\right)$$

since $E_{1}\left(C_{1}\right)=E_{1}\left(C_{2}\right)$ . Remarkably, 28 (!) circuitnets in the power set 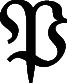 $\left(\{C_{1},C_{2},C_{3},C_{4},C_{5}\}\right)$ —all except for the empty set and the three singletons {*C*_1_}, {*C*_2_}, and {*C*_3_}, which correspond to elementary circuits involving only two species—induce the same CS matrix (eq 18) and therefore belong to the same equivalence class. Notably, even distinct elementary circuits, such as *C*_4_ and *C*_5_ in this example, can be CS-equivalent. En passant, we further note that all autocatalytic cores **S**[**κ**], with the sole exception of type III, admit a single-circuit representative in the CS-equivalence class of circuitnets associated with **S**[**κ**], see Supporting Information Example 1. The next Lemma is central in exploiting the CS-equivalence to lighten the computational cost of our approach.

**Lemma 35** (Proof: Supporting Information). Let $C_{1}$ and $C_{2}$ be circuitnets for fluffles *G*_1_ and *G*_2_, respectively, and let *C*′ and *C*″ be two elementary circuits. Assume $C_{1}$ 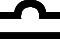 $C_{2}$ , *C*′ 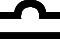 *C*″ and $G_{1}^{\prime }≔$ 
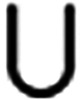
 $\left(C_{1}\cup \left\{C^{\prime }\right\}\right)$ is a fluffle. Then $G_{2}^{\prime }≔$ 
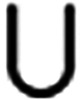
 $\left(C_{2}\cup \left\{C^{\prime }\right\}\right)$ is a fluffle as well with $C_{1}\cup \left\{C^{\prime }\right\}$ 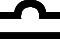 $C_{2}\cup \left\{C^{\prime \prime }\right\}$ .

The converse of Lemma 35 does not hold. A counterexample is again the autocatalytic core of type V (30) depicted in Figure 6. Consider the two circuitnets $C_{2}≔\left\{C_{4},C_{5}\right\}$ and $C_{3}≔\left\{C_{1},C_{2}\right\}$ . Note that the circuitnets $C_{3}$ is obtained from $C_{1}=\left\{C_{1},C_{2},C_{3}\right\}$ by removing the elementary circuit *C*_3_, red in Figure 6. Even if $C_{2}$ 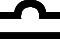 $C_{3}$ holds, the parallel removal of any elementary circuit from the two circuitnets destroys the equivalence relation as *C*_*i*_ 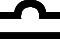 *C*_*j*_ does not hold for *i* = 4,5, and *j* = 4,5: the elementary circuits in $C_{2}$ comprise three species while the elementary circuits in $C_{3}$ comprise two.

However, we may still ensure that a representative of each CS-equivalence class can be reached by adding an elementary circuit to the representative of some CS-equivalence class of a smaller fluffle. More precisely, we have the following Lemma.

**Lemma 36** (Proof: Supporting Information). For every CS-equivalence class [C] there is a representative Ĉ such that there exists a CS-equivalence class $\left[C^{\prime }\right]$ with representative ${Ĉ}^{\prime }$ and an elementary circuit C* such that ${Ĉ}^{\prime }\cup \left\{C^{*}\right\}$ 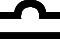 Ĉ and​ ​ |V( 
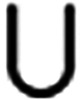
 $\left(C^{\prime }\right))|<|V($ 
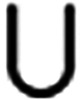
 (C))| .

Finally, we may naturally extend the notion of CS-equivalence to fluffles.

**Definition 37**. Two fluffles *G*, *G*′ are CS-equivalent if and only if *E*_1_(*G*) = *E*_1_(*G*′).

Moreover, we observe that each 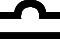-equivalence class has a natural fluffle representative, whose graph is indeed **K**(κ):

**Proposition 38**. Let G be a fluffle and let **κ** be the CS defined by *E*_1_(*G*). Consider now the graph *G̅* with vertex set V(G̅)=V(G) and edge set *E*_1_(*G*) ∪ *E*_2_(*V*(*G*)), i.e., *e* ∈ *E*_2_(*V*(*G*)) if and only if *e* = (*r*_1_, *x*_2_) with r_1_, *x*_2_ ∈ *V*(*G*). Then *G̅* is a fluffle with *G̅* 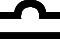 *G*, and such that G̅ = K­(κ).

Proof. Since *G* is a fluffle of **K**, then *E*_1_(*G*) is a perfect matching corresponding to the CS **κ**, and thus G̅ = K­(κ) by eq 14. By Prop. 26, *G̅* is a fluffle as well. As $E_{1}\left(\mathrm{G̅}\right)=E_{1}\left(G\right)$ holds by construction, we have *G̅* 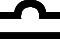 *G*.

□

Taken together, we can avoid the enumeration of 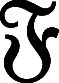 in favor of enumerating fluffle representatives only.

### Metzler Matrices and Induced Fluffles

Recall that by Prop. 14 **S**[**κ**] corresponds to the induced subgraph **K**[**κ**], while 

 (S[κ]) corresponds to the fluffle **K**(**κ**) defined on the same vertex set $X_{\kappa }\cup ̇R_{\kappa }$ . Therefore, **K**(**κ**) is always a spanning subgraph of **K**[**κ**]. However, in general, we have **K**[**κ**] ≠ **K**(**κ**), as shown in Figure 2. Nevertheless, many properties of **K**(**κ**) translate to the induced subgraphs. In this section, we collect some of these implications, which will be useful below in the context of autocatalytic cores.

As an immediate consequence of Lemma 19 we obtain.

**Corollary 39**. An induced subgraph $K\left[X^{\prime }\cup ̇R^{\prime }\right]$ is child-selective if and only if it contains a spanning subgraph *G* with edge set *E*′ such that *E*′ ∩ (*X*′ × *R*′) is a perfect matching in *G*.

**Theorem 40**. An induced subgraph **K**′ ⊆ **K** has a Metzler CS matrix **S**[**κ**′] if and only if *E*_1_(**K**′) is a perfect matching in **K**′.

Proof. If *E*_1_ is a perfect matching in **K**′ then each *x* ∈ *X*(**K**′) has only one outgoing edge and each *r* ∈ *R*(**K**′) has only one incoming edge. Hence, for each *r* ∈ *R*(**K**′) there is a unique *x* ∈ *X*(**K**′) with *s*_*xr*_^–^ > 0, while for all *y* ∈ *X*(**K**′) with *x* ≠ *y*, *s*_*yr*_^–^ > 0. Thus, S[κ]= 

 (S[κ]) and hence a Metzler matrix. If, on the other hand, **S**[**κ**] is a Metzler matrix, for each *r* ∈ *R*(**K**′) there is exactly one *x* ∈ *X*(**K**′) with *s*_*xr*_^–^ > 0, which implies that *r* has exactly one incoming edge given by (κ^–1^(*r*), *r*). Since κ is a bijection, κ^–1^ is in particular injective and hence these edges form a perfect matching, which by construction coincides with *E*_1_.

□

If S[κ]= 

 (S[κ]) then **K**(**κ**) already contains all edges of **K**[**κ**]. Conversely, if **K**(**κ**) = **K**[**κ**], then *E*_1_ is a perfect matching in this subgraph and hence **S**[**κ**] is Metzler.

**Corollary 41**. Let **κ** be a CS. Then the following statements are equivalent:

(i) **S**[**κ**] *is a* Metzler matrix
(ii) **K**[**κ**] = **K**(**κ**).

Since **K**(**κ**) is a spanning subgraph of **K**[**κ**], the following to observations are straightforward:

**Corollary 42**. If **κ** is a CS and 

 (S[κ]) is irreducible then **K**[**κ**] is strongly connected.

**Corollary 43**. If **κ** is a CS and 

 (S[κ]) is irreducible then **K**[**κ**] is a strong block.

The statement of Lemma 23, however, does not hold for **K**[**κ**] (see Figure 4, right). That is, a strongly connected induced subgraph **K**[**κ**] is not necessarily a strong block.

Moreover, the fact that **K**[**κ**] is strongly connected does not imply that 

 (S[κ]) is irreducible. As a counterexample, consider the disjoint union of two even elementary circuits, one containing a substrate vertex *x*_2_ and the other *x*_3_ (Figure 3, right). This graph is child-selective, with κ­(*z*) defined as the successor of each substrate vertex *z* along its circuit. Adding the two edges (*x*_2_, κ­(*x*_3_)) and (*x*_3_, κ­(*x*_2_)) (blue edges in Figure 3, right) produces a strongly connected graph that remains child-selective under the same map κ. Nevertheless, **K**(**κ**) remains the disjoint union of the two circuits and thus is not strongly connected. In this example, an alternative child-selection exists by defining κ′(*z*) = κ­(*z*) for *z* ∉ {*x*_2_, *x*_3_}, κ′(*x*_2_) = κ­(*x*_3_), and κ′(*x*_3_) = κ­(*x*_2_), though such a construction is not always possible. If only (*x*_3_, κ­(*x*_2_)) of the two blue edges was present, the subgraph would still be strongly connected if there existed an additional edge (*x*_2_, *r*) to a reaction *r* ≠ κ­(*x*_3_) in the second circuit (green edge). Suppose there is a perfect matching *M* that includes (*x*_2_, *r*). Then necessarily (κ^–1^(*r*), *r*) ∉ *M*. Since κ^–1^(*r*) ≠ *x*_3_, the vertex κ^–1^(*r*) has only one successor, *r*, along its circuit, implying that *M* cannot be a perfect matching in *E*_1_.

We can, however, rephrase Lemma 25 in terms of the induced subgraph **K**[**κ**], making use again of the fact that **K**(**κ**) is a spanning subgraph of **K**[**κ**].

**Corollary 44**. A CS **κ** = (*X*_κ_, *E*_κ_, κ) is autocatalytic if and only if the following conditions both hold:

1. there is a positive vector *v* > 0 such that **S**[**κ**]*v* > 0.
2. **K**[**κ**] does not possesses source and sink vertices;

Recall that autocatalytic cores are, in particular, irreducible Metzler CS matrices. Autocatalytic cores thus satisfy S[κ]= 

 (S[κ]) , i.e., **K**[**κ**] = **K**(**κ**). In other words, all autocatalytic cores correspond to induced fluffles. Prop. 38 thus implies that the natural representative of the CS-equivalence class of an irreducible Metzler CS matrix, and hence in particular of an autocatalytic core, is an induced fluffle.

Finally, we show that irreducible Metzler CS matrices **S**[**κ**] are autocatalytic whenever they contain an autocatalytic core. Our starting point is the following technical result, which then enables us to state the main result of this subsection.

**Lemma 45** (Proof: Supporting Information). Let **K**(κ*) be a fluffle with irreducible autocatalytic Metzler CS matrix **S**[κ*] and let **K**(**κ**) be obtained from **K**(κ*) by adding a single ear with initial vertex in R­(**K**(κ*)), terminal vertex in *X*(**K**(κ*)), and a nonempty set of internal vertices, together with all reaction-to-metabolite edges in *R*(**K**(**κ**)) × *X*(**K**(**κ**)). If **S**[κ*] is an autocatalytic CS matrix and **S**[**κ**] is a Metzler matrix, then **S**[**κ**] is autocatalytic irreducible CS matrix.

**Theorem 46** (Proof: Supporting Information). Let **S**[**κ**] be an irreducible Metzler CS matrix and suppose **S**[**κ**] contains an autocatalytic core **S**[κ*] as a principal submatrix. Then **S**[**κ**] is autocatalyic.

We conclude this section with a close look at autocatalytic cores. Consider a fluffle *G* and recall that for any fluffle *H* with *E*_1_(*H*) ⊆ *E*_1_(*G*) the matrix **S**[*E*_1_(*H*)] is a principal submatrix of **S**[*E*_2_(*G*)]. Since an autocatalytic core is irreducible and thus a superposition of elementary circuits, we immediately observe the following corollary:

**Corollary 47**. Let G be an autocatalytic core in **K** and C a circuitnet for G. Then **S**[*E*_1_(*C*)] is a Metzler CS matrix for every C∈C .

It is therefore of interest to consider the following subclass of elementary circuits:

**Definition 48**. A Metzler circuit in K=(X∪̇R,E) is an elementary circuit without a chord of the form (*x*, *r*) ∈ *X* × *R*.

Cor. 47 thus implies that every autocatalytic core can be constructed from the Metzler circuits in **K** alone. In a recent follow-up paper, we show that this is indeed feasible in combination with a specialized algorithm for enumerating partially chordless circuits.^55^

## Algorithms

### Basic Algorithms

#### Elementary Circuits

Johnson’s algorithm^32^ enumerates all elementary circuits of a directed graph with linear delay. Since we only need an arbitrary representative of each CS-equivalence class, it suffices to record the sets *E*_1_(*C*). We denote the set of representatives by **C**.

#### Recognition of Fluffles

Based on Thm. 29 and Prop. 38, the key task is to expand a representative fluffle *G* by a representative circuit *C* and to test whether the union *G* ∪ *C* is again a fluffle. The following result greatly simplifies this task:

**Lemma 49** (Proof: Supporting Information). Let *G* be a fluffle and C an elementary circuit. Then *G* ∪ C is a fluffle if and only if Ø ≠ *V*(*G*) ∩ *V*(*C*) = *V*(*E*_1_(*G*) ∩ *E*_1_(*C*)).

As a consequence of Lemma 49, it suffices to consider only the edge sets *E*_1_(*G*) and *E*_1_(*C*), and their incident vertex sets when constructing representatives of CS-equivalence classes of fluffles. This considerably simplifies the practical implementation since we do not have to maintain graph data structures for the fluffles. By Prop. 38, we may use *G̅* = **K­(κ)** as canonical representative of [*G*], where **κ** is the CS defined by *E*_1_(*G*). If desired, circuitnets for the fluffle *G̅* can be reconstructed in linear time by means of a directed ear decomposition.

#### Representatives of CS-Equivalence Classes

As a consequence of Lemma 36 and Def. 37, a representative fluffle for each CS-equivalence class of 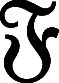 can be obtained, sparsely, by computing unions of elementary circuits with representative fluffles of CS-equivalence classes with fewer vertices. To this end, we start from the set **C** of representatives of elementary circuits and initialize a queue *Q* with these elementary circuits. *Q* will contain an arbitrary representative of each CS-equivalence class of fluffles for further expansion. We maintain a separate set ε of all representatives as the output of the algorithm. The queue *Q* is processed in first-in-first-out order.

For each *G* ∈ *Q*, all representative elementary circuits *C* ∈ **C** are tested for *G* ∪ *C* constituting a fluffle by means of Lemma 49. If so, *G*′ = *G* ∪ *C* serves as a representative of the CS-equivalence class [*G*′]. If there is a fluffle $G^{\prime \prime }\in E$ such that *G*″ ∈ [*G*′], i.e., *E*_1_(*G*″) = *E*_1_(*G*′) then *G*′ is discarded without changes to *Q*. Otherwise [*G*′] is appended to *Q*. If a concurrent hashset to *Q* is maintained with all representative edgesets as keys, then the comparison of *E*_1_(*G*′) and *E*(*G*″) can be performed in constant time. The algorithm terminates when *Q* is empty, i.e., all maximal fluffles have been found. Finding overlapping elementary circuits *C* for each fluffle *G* can be sped up by a hashmap *M* linking edges in *E*_1_ to sets of circuits they are contained in. We only have to consider pairs where at least one such edge is shared. A pseudocode for this procedure is given in Alg. 1.

#### Identification of Autocatalytic Matrices and Cores

Each entry in E obtained by Alg. 1 constitutes a candidate **S**[**κ**] for an irreducible autocatalytic CS matrix. Since every irreducible autocatalytic CS matrix **S**[**κ**] contains an autocatalytic core, it either satisfies S[κ]= 

 (S[κ]) or it strictly contains a principal submatrix **S**[**κ**′] with this property. In the latter case, **κ**′ is a restriction of **κ** and thus **K**(**κ**′) is a proper subgraph of **K**(**κ**). In fact, by Cor. 41, **K**(**κ**′) = **K**[**κ**′] must also be an induced subgraph of **K**(**κ**). Moreover, we have *E*_1_(**K**(**κ**′)) ⊆ *E*_1_(**K**(**κ**)) if and only if **κ**′ is a restriction of **κ**. Thus, we have the following necessary condition:

**Corollary 50**. If *G* is a fluffle that defines an autocatalytic CS matrix, then there is an induced subgraph *G*′ of *G* such that *G*′ is also an induced subgraph of **K**.

On the other hand, if **S**[*E*_1_(*G*)] is a Metzler matrix, i.e., if *G* is an induced fluffle representative, and *G* contains an autocatalytic core, then *G* is itself autocatalytic by Thm. 46.

These simple observations suggest computing the Hasse diagram with respect to set inclusion, Hasse(E) , for sets E of fluffle equivalence classes. Traversing Hasse(E) in bottom-up order, one then checks, for each ⊆-minimal candidate *G* in E :

(a) whether *G* = **K**[*V*(*E*_1_(*G*))], which is equivalent to *G* giving rise to a Metzler CS matrix **S**[*E*_1_(*G*)], and, if so,
(b) whether **S**[*E*_1_(*G*)] is Hurwitz unstable.

A minimal element in (E,⊆) that satisfies (a) and (b) is an autocatalytic core. Moreover, any Metzler matrix that contains an autocatalytic submatrix is automatically autocatalytic, and in this case, test (b) can be omitted. Taken together, an explicit test for autocatalyticity needs to be performed only for inclusion-minimal induced fluffle representatives that are Metzler and for nonminimal non-Metzler matrices in E . If one of the conditions (a) or (b) is violated, *G* is removed from Hasse(E) and each parent of *G* is connected to each immediate descendant of *G*. Upon completion of the traversal, all minimal elements in Hasse(E) are autocatalytic cores and thus Metzler matrices. Moreover, all descendants of a Metzler matrix in Hasse(E) are again Metzler. Similarly, all parents of a non-Metzler matrix are again non-Metzler. For each of the non-Metzler matrices we explicitly test whether they are autocatalytic using an LP solver^56^ to determine whether there is a vector *v* > 0 such that **S**[**κ**]*v* > 0.

Determining the complete structure of the Hasse diagram, however, severely compromises performance, and simply testing all CS-equivalence classes for their autocatalytic capacity would be more efficient. In contrast, predecessor relations are sufficient to avoid unnecessary testing and can be obtained without additional costs. By Alg. 1, for each element *G* retrieved from *Q*, subset relations with all CS-equivalence classes of elementary circuits intersecting in at least one *e* ∈ *E*_1_(*G*) are determined. These subset relations, however, define predecessor relations in Hasse(E) and can be utilized to avoid unnecessary testing for autocatalysis as suggested. By construction, only CS-equivalence classes of elementary circuits can be leaves. If their set of predecessors is empty, they can be excluded from testing whenever their associated CS matrix is non-Metzler. In the Metzler case, all predecessors are recursively screened for the autocatalytic capacity of their associated Metzler matrix. Whenever this is the case, the search is stopped and testing can be omitted. Additional flags avoid visiting and testing an element twice.

### Direct Enumeration of Autocatalytic Cores

Since autocatalytic cores are necessarily Metzler matrices, it is possible to modify Alg. 1 to enumerate autocatalytic cores only: first, the enumeration of elementary circuits is restricted to Metzler circuits (Def. 48) since by Cor. 47 every induced fluffle, and thus every candidate for an autocatalytic core is a union of Metzler circuits. Moreover, if *C*_1_, *C*_2_, ..., *C*_*n*_ is a circuitnet for a Metzler fluffle *G* and each *C*_*i*_ is a Metzler fluffle, then any fluffle *G*_*k*_ = 
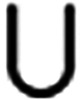
_*i*=1_^*k*^ *C*_*π*(*i*)_ (Cor. 32) leads to a corresponding Metzler matrix **S**[*E*_1_(*G*_*k*_)] since *G*_*k*_ is a fluffle subgraph of an induced fluffle. Thereby, **S**[*E*_1_(*G*_*k*_)] is a principal submatrix of a Metzler matrix and hence itself a Metzler matrix. Therefore, all non-Metzler fluffles can be discarded immediately. Moreover, if *E*_1_(*G*) is an autocatalytic core, none of its extensions can be cores. Hence, *E*_1_(*G*) is only pushed on the queue *Q* if it corresponds to an induced fluffle and **S**[*E*_1_(*G*)] is not autocatalytic, while only the induced autocatalytic cases are added to the output E . This procedure, however, is not guaranteed to detect all predecessor relationships between autocatalytic matrices. The resulting false positive candidates can be identified in a postprocessing step by checking whether there are subset relationships among the core candidates in E . This inclusion testing can be parallelized to increase efficiency. However, empirical tests revealed that a different strategy performs better: pushing all elementary circuits and larger fluffles, along with their associated autocatalytic Metzler CS matrices, into the queue while restricting their processing to inclusion-relation detection only. This approach drastically reduces the number of required set-inclusion tests between candidates and therefore offers a substantial performance advantage.

### Extensions for Large CRNs

With increasing network size, the number of expected elementary circuits grows exponentially. Exhaustive enumeration of elementary circuits as required by Alg. 1 therefore becomes infeasible for large CRNs. A natural restriction is to limit the size of circuits, at the cost of also limiting the size of resulting fluffles in the assembly. In biochemical networks, one may expect that autocatalytic subsystems are predominantly confined to functional modules or pathways. We therefore aim to enumerate circuits first within such modules and only then extend the search for circuits to connections between modules. We proceed in two steps: the network is clustered and elementary circuits within units are enumerated exhaustively. Then circuits crossing (typically sparsely connected) borders of neighboring clusters (as identified by the cluster partition tree) are enumerated with size restrictions.

A useful decomposition of a CRN should ideally generate subnetworks of roughly equal size while preserving the cycle structure within modules as much as possible. Moreover, as mentioned, the modules should be biochemically meaningful, i.e., encapsulate specific metabolic functions. This problem has received considerable attention in applications to metabolic networks.^38,57,58^ Here, we reimplemented the partitioning algorithm described previously,^38^ which is based on spectral methods.^59^ A detailed description and pseudocode for cluster and cycle enumeration algorithms are provided in Supporting Information Section Algorithmic Overview.

### Implementation Details

The algorithms detailed above are implemented as a Python package autogato. It is structured in different submodules. First, a metabolic model is imported in xml-format in partitionNetwork.py via libsbml^60^ and translated into a networkX^61^ DiGraph. After modularisation by means of leading eigenvector computations using NumPy^62^ in partitionComputations.py, the partition tree and all relevant parameters are pickled and saved separately for each strongly connected component. In the second step, each strongly connected component is now analyzed separately in the module partitionAnalysis.py. The submatrix of the stoichiometric matrix is extracted, and then the set E of elementary circuits enumerated. Depending on the strategy chosen by the user, the associated CS matrices and their autocatalytic capacities are determined concurrently or downstream after assembly. During assembly, feasible combinations of CS-equivalence and CS-equivalence classes of elementary circuits are combined. Finally, autocatalytic capacity is computed using the real part of the largest eigenvalue or by solving a linear programming problem with SciPy.^56^ If feasible, partitioning, enumeration of elementary circuits, and assembly of larger equivalence classes is processed in parallel using ConcurrentFutures ProcessPoolExecutors. Heavy computations are processed with Cython.^63^

### Showcase Applications

To demonstrate the performance of autogato, and thus the practical use of the algorithms described above, we investigated four metabolic networks: the *E. coli* core model,^39^ a larger model of *E. coli* DH5α,^64^ a metabolic model of human erythrocytes,^65^ and a model of the archaeon *Methanosarcina barkeri*.^66^ In all cases, we removed small, highly connected molecules (e.g., CO_2_ and H_2_O) as well as exchange metabolites such as ADP and NADH, since they are of little relevance for the biological interpretation of autocatalysis. A full list for each model is provided in S1 Section Additional Computational Data.

Our version of the *E. coli* core metabolism CRN comprises 36 metabolites and 71 reactions. Its König graph contains 2021 elementary circuits, all belonging to distinct CS-equivalence classes. The enumeration algorithm identified 202,206 fluffles, grouped into 8551 CS-equivalence classes. For a summary of their size distribution, we refer to Figure 7. Even for relatively small networks, restricting to representatives of CS-equivalence classes provides a drastic reduction in computational resources: fluffle enumeration took about 12,500 s, while enumeration of CS-equivalence classes required only about 25 s and only 5 s when being computed in parallel. In total, autogato required 17.4 s for completion, including decomposition of the network and construction of the stoichiometric matrix from the reaction data.

**Figure 7:**
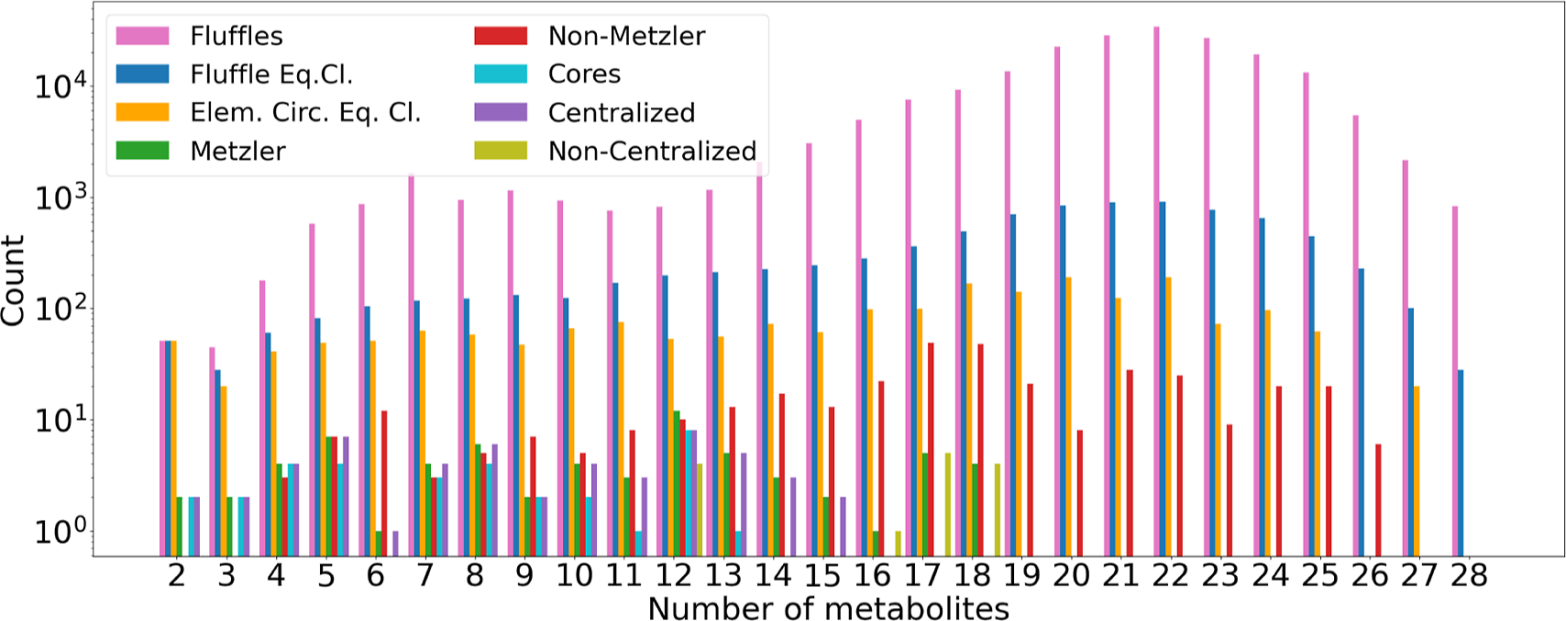
Size distribution of CS-equivalence classes for elementary circuits and fluffles, and autocatalytic Metzler and non-Metzler matrices in the *E. coli* core metabolism. See Figure S3 for additional details.

Autocatalytic subsystems are common in metabolic networks. In the central carbon metabolism, represented by our *E. coli* core model, 158 of the 2021 elementary circuits (7.9%) are autocatalytic. Of these, 42 are associated with Metzler CS matrices and 114 with non-Metzler CS matrices. Overall, approximately 5% (426 of 8551) of CS-equivalence classes are autocatalytic; 67 of these have a Metzler **S**[**κ**], while 359 are non-Metzler. Of the 67 autocatalytic Metzler matrices, 53 were centralized and only 14 were noncentralized (see Def. 52 below for more details). Interestingly, the ratio of autocatalytic Metzler CS to non-Metzler matrices roughly halves when moving from elementary circuits to all equivalence classes, from 1/3 to 1/6, which corresponds to the overall decrease in the fraction of equivalence classes associated with Metzler matrices, from 6.4% to 1.8%. Among the 67 autocatalytic CS-Metzler matrices, we identified 33 autocatalytic cores. One of these, shown in Figure S5, is an autocatalytic core of Type IV. Previously, no example of this type had been reported in the literature.^16,67^

We compared our implementation with the ILP formulation of Gagrani et al.^18^ To this end, we computed the stoichiometric matrix and passed these data as input to the ILP, which found 31 autocatalytic cores in 1.69 s. Restricting autogato to enumerating autocatalytic cores exclusively, only 0.342 s (averaged over 1000 iterations) were required, and 33 cores were identified, which included all found by Gagrani et al.^18^ The two additional cores are depicted in Figure S4 of the Supporting Information. We comment on potential reasons for these differences in the Appendix below.

The larger *E. coli* DH5α network comprised 2779 reactions and 1951 metabolites. After removing small and highly connected metabolites (see Supporting Information Section Additional Computational Data), as performed for the *E. coli* core network, 10 strongly connected components with at least 2 reactions remained. In total, we retained 1142 reactions and 622 metabolites. The largest strongly connected component comprised of 568 metabolites and 1061 reactions. Overall, 2,647,664 CS-equivalence classes with at most 10 metabolites and 10 reactions could be detected; the majority (94.8%; 2, 516, 295) comprised CS-equivalence classes with associated non-Metzler matrices; 161, 589 (6.4%) of them autocatalytic and 2, 354, 706 (93.6%) nonautocatalytic. In contrast, 131, 369 (5.2%) of the enumerated CS-equivalence classes were associated with a Metzler matrix; 109, 391 (83.3%) autocatalytic and only 21, 978 (16.7%) nonautocatalytic matrices. The majority of autocatalytic Metzler matrices (56%; 61,903) form autocatalytic cores. Centralized autocatalysis dominated with 57% noncentralized autocatalysis with 43% slightly among the autocatalytic Metzler matrices. Overall, 10% of all CS-equivalence classes were autocatalytic. The size distributions are depicted in Figure 8. In total, autogato required 1 h:49 min:24 s with maximum consumption of 16.9 Gb internal memory. To compare with the ILP of,^18^ we used the same approach as for the smaller network and supplied the stoichiometric matrix of the largest strongly connected component as input. After 14 h of running time, 4700 autocatalytic cores had been enumerated up to a size of 8 species and reactions. At this point, 20 s were required for the computation of the next core. We, therefore, terminated the enumeration process. In contrast, the restriction of our algorithm to enumerating only cores took 4 min:5 s with a maximum memory consumption of 302 Mb. The majority of the time was spent on the enumeration of elementary circuits, while assembly of larger equivalence classes and postprocessing finished in 26 s and 17 s, respectively.

**Figure 8:**
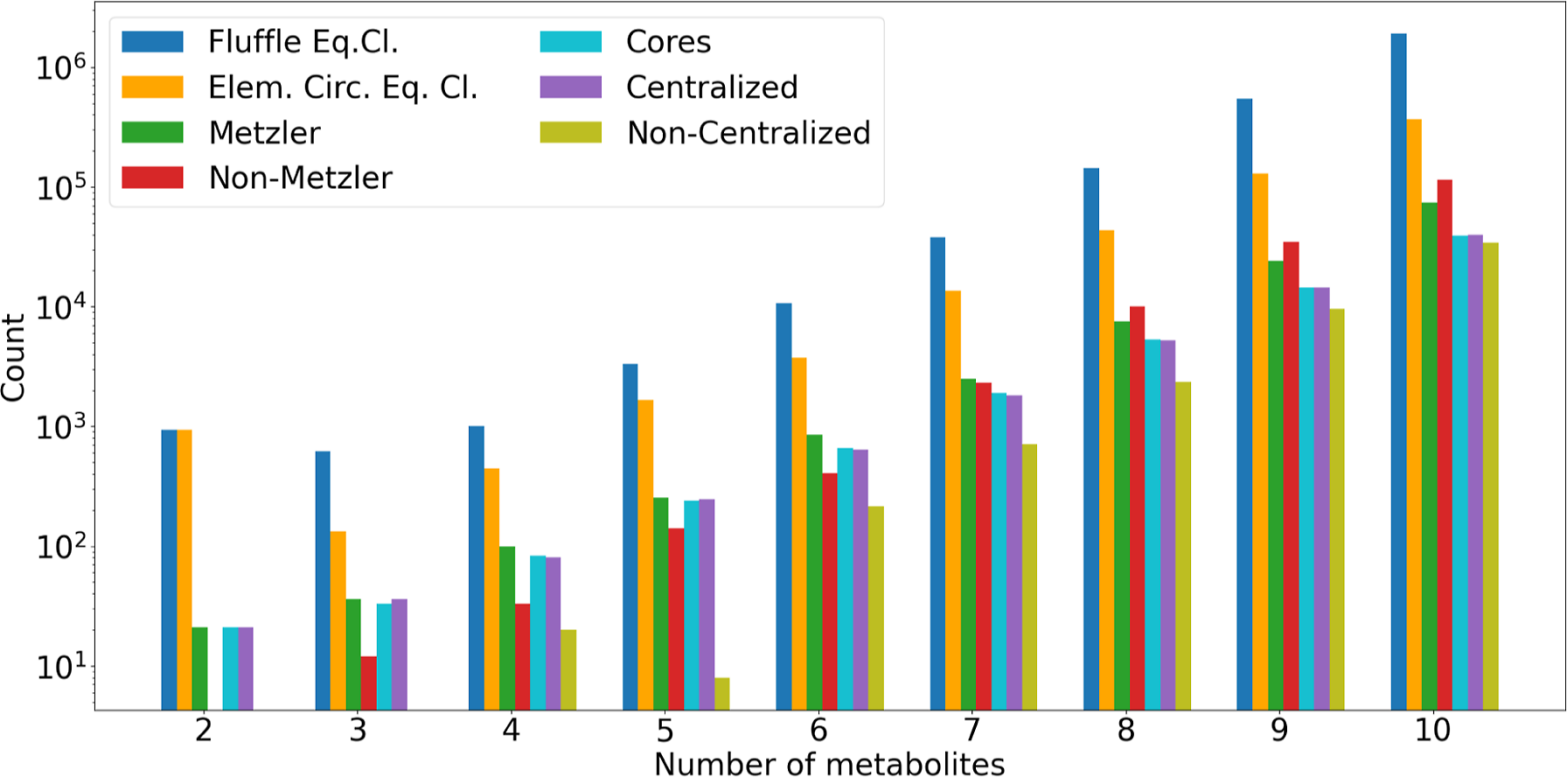
Size distribution of CS-equivalence classes for elementary circuits and fluffles, as well as autocatalytic CS-Metzler and non-Metzler matrices in the largest connected component of our modified *E. coli* *DH*5α network.

To investigate the frequency of autocatalysis in nonbacterial species, we applied the algorithm to another network, of human erythrocytes,^65^ which contained 342 metabolites and 469 reactions. After removal of all small metabolites, two larger strongly connected components remained: one composed of 69 metabolites and 112 reactions, respectively, covering central carbon metabolism, including glycolysis, PPP, and amino-acid metabolism, and a second component composed of 72 metabolites and 135 reactions, largely covering lipid metabolism. In summary, 1,379,913 CS-equivalence classes with a maximum size of 25 metabolites/reactions were enumerated. In contrast to the *E. coli* networks, only 940 (0.068%) were autocatalytic. The network reflecting central carbon metabolism exhibited approximately 8% (103/1258) autocatalytic CS-equivalence classes (35/150 Metzler and 113/183 non-Metzler), which is in line with the results obtained from the *E. coli* core network. However, the network reflecting lipid metabolism contained 1,378,647 CS-equivalence classes, of which only 0.06% (837) were autocatalytic; of these 156 are Metzler and 681 non-Metzler matrices. Size distributions for both networks together are depicted in Figure 9.

**Figure 9:**
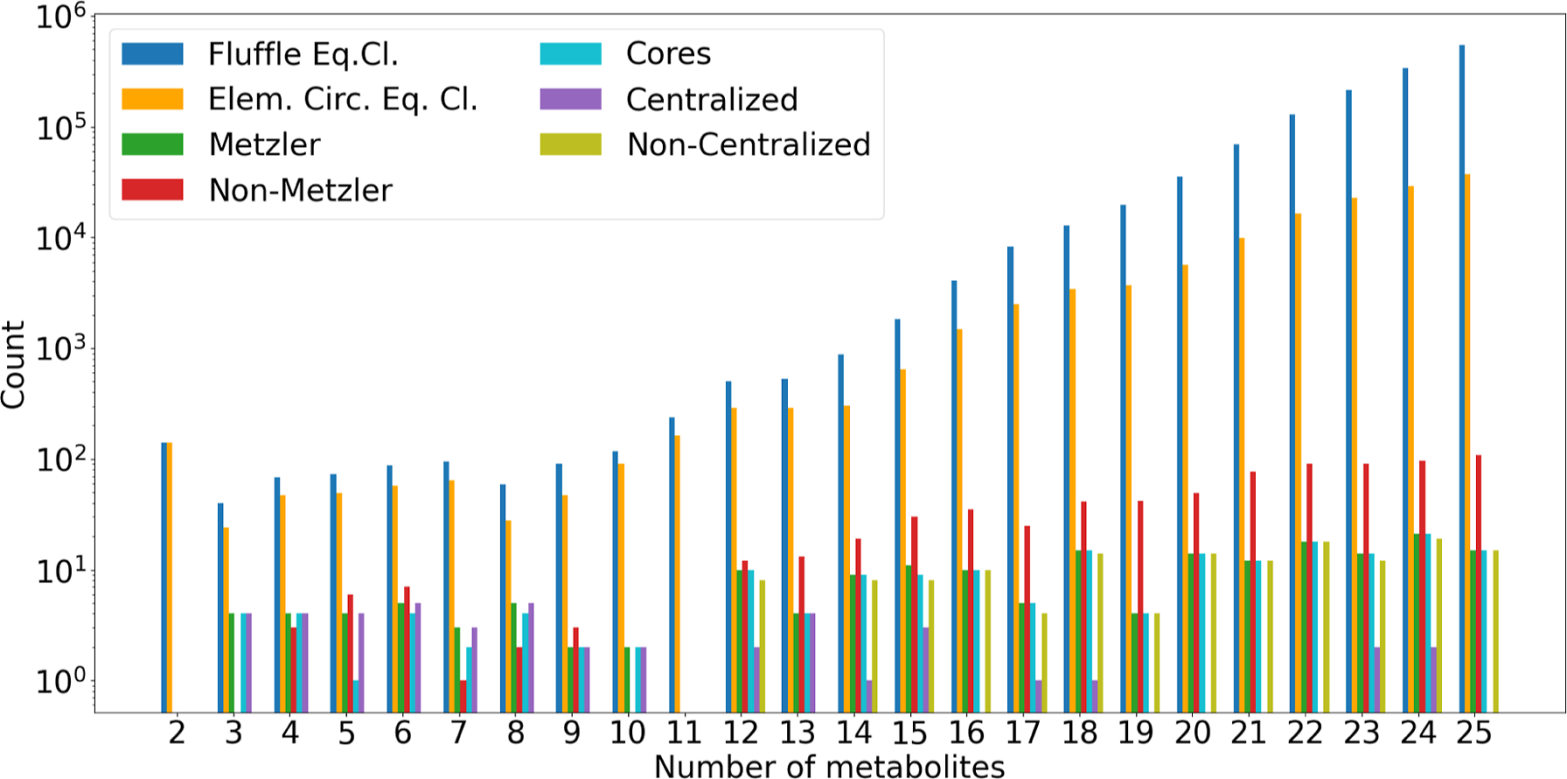
Size distribution of CS-equivalence classes for elementary circuits and fluffles, as well as autocatalytic CS Metzler and non-Metzler matrices in the largest connected component of our modified erythrocyte network.

Finally, we investigated whether autocatalysis could be found in a member of the Archaea domain. To this end, we took advantage of the metabolic model of *Methanosarcina barkeri* ^66^ with 690 metabolites and 692 originally, of which 249 and 402, respectively, remained in the largest strongly connected component. We restricted the size of all CS-equivalence classes to 10 metabolites and reactions. Within this connected component, only 1.2% (20,194) of all CS-equivalence classes (1,677,604) were found to be autocatalytic; 4105 with a Metzler and 16,089 with a non-Metzler matrix. Nearly three-quarters of the Metzler matrices (5483) were autocatalytic, while for the non-Metzler matrices this is the case for only 1% (16,089/1,656,158). Two-thirds of the CS-equivalence classes with autocatalytic Metzler matrices correspond to autocatalytic cores. The size distribution is depicted in Figure 10.

**Figure 10:**
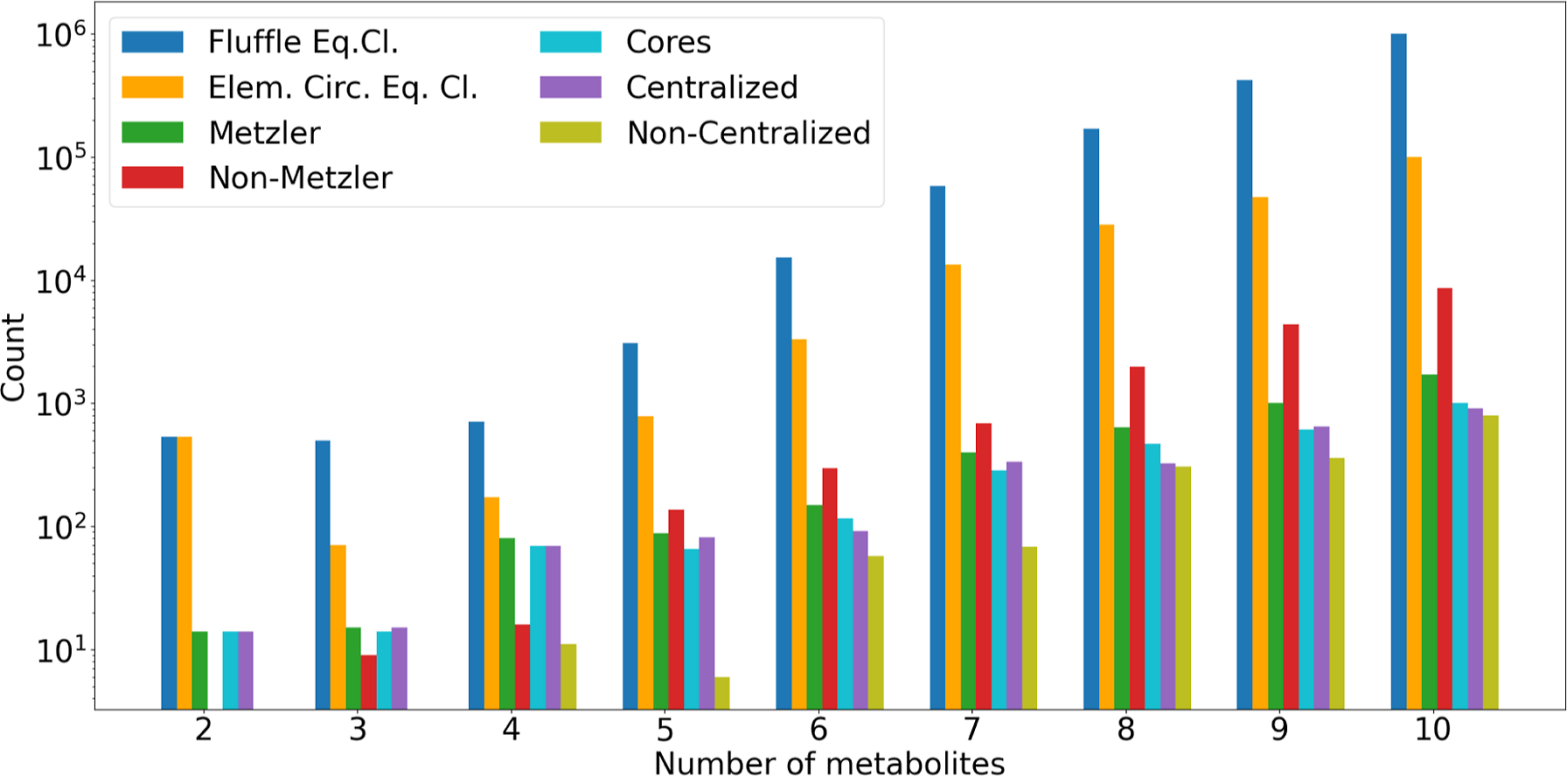
Size distribution of CS-equivalence classes for elementary circuits and fluffles, as well as autocatalytic CS-Metzler and non-Metzler matrices in the largest connected component of our modified *Mathanosarcina barkeri* network.

## Discussion

We have presented a detailed mathematical analysis of autocatalytic substructures in large CRNs. Starting from the stoichiometric matrix **S**, we identify a specific class of subgraphs in the bipartite (König) representation of the CRN, called fluffles, which are necessary to support irreducible autocatalytic subnetworks. Fluffles fall into equivalence classes determined solely by the corresponding child-selections. These correspond to Metzler matrices that form autocatalytic cores if and only if they are induced subgraphs of the CRN, while larger irreducible autocatalytic subnetworks only need to contain a Metzler part, or equivalently, a spanning fluffle, as well as a smaller autocatalytic core.

Based on these structural insights, we developed an algorithmic approach to produce representative fluffles by superimposing elementary circuits. This purely graph-theoretical method avoids the complex ILP formulation used previously to detect autocatalytic cores.^18,21^ Furthermore, it extends to a much broader class of autocatalytic subsystems beyond the autocatalytic cores. Tests on four metabolic networks, a small model of the *E. coli* core metabolism and three much larger CRNs comprising up to more than 600 metabolites and 1100 reactions showed that our algorithmic approach is feasible in practice. For the small network, a complete analysis is obtained within about 17 s. For the large network, the computation had to be limited to moderate-size fluffles, with up to 10, 10, and 25 metabolites and reactions for *E. coli*, *Methanosarcina barkeri*, and human erythrocytes, respectively. Clearly, this does not capture all autocatalytic cores, since cores in the smaller *E. coli* network ranged up to 13 metabolites, i.e., almost half of the size of the CRN. Nevertheless, in the *E. coli* *DH*5α model, we identified more than 100,000 irreducible autocatalytic CS subnetworks, more than half of which are autocatalytic cores. These results reinforce the conclusion of earlier studies (in particular those based on different definitions of autocatalysis^14^) on the ubiquitous nature of autocatalysis in metabolic CRNs.

A direct comparison of the restricted variant of Alg. 1 that computes autocatalytic cores only with the ILP formulation^18^ turned out favorably for our approach with respect to resource consumption. An evaluation of the *E. coli* core network shows furthermore that the ILP does not enumerate all autocatalytic cores and struggles with larger network sizes. In fact, the algorithm described previously^18^ focuses on enumerating only the minimal subsets of reactions that contain an autocatalytic core, without imposing any restriction on the species involved. Consequently, for reaction sets *R*_1_ ⊂ *R*_2_ associated with two autocatalytic cores *A*_1_ and *A*_2_ based on different sets of species, the ILP formulation would identify only the minimal set *R*_1_. We briefly elaborate on this issue in the Appendix.

Once autocatalytic subsystems have been identified, they can provide further insight into the potential behaviors of the CRN. For example, the close connection between autocatalysis and sustained oscillations has been explored before.^68^ Building on this work, one can state the following sufficient condition:

**Proposition 51** (Proof: Supporting Information). Let **S**[**κ**] be a Hurwitz-stable autocatalytic CS matrix. Then there exists a choice of parameters such that the system (5), *ż* = *f*(z)≔ *S*·*v*(*z*), admits periodic solutions.

This result sets the stage for identifying minimal subnetworks that are responsible for “interesting” dynamical behavior such as periodic oscillations.

For large CRNs, in particular models of complete metabolisms, an exhaustive enumeration of fluffle CS-equivalence classes is probably infeasible even on an HPC system. This is certainly true in the (chemically unrealistic) worst-case scenario, since it is possible to construct CRNs in which all autocatalytic cores have size 2 but there are exponentially many autocatalytic Metzler matrices: it suffices to consider a CS matrix **S**[**κ**] such that **S**[**κ**]_ii_ = −1, **S**[**κ**]_*ij*_ = 2 for *i* > *j*, and **S**[**κ**]_*ij*_ = 1 for *i* < *j*; in this case, only the 2 × 2 principal submatrices of **S**[**κ**] are autocatalytic cores while every principal submatrix of **S**[**κ**] is an irreducible, autocatalytic Metzler matrix.

By Cor. 47, all autocatalytic cores are superpositions of Metzler circuits. Since worst-case instances may also contain very large numbers of elementary circuits, this raises the question of whether it is possible to enumerate the subset of Metzler circuits without enumerating all elementary circuits. In our analysis of the large *E. coli* network, we pragmatically limited the length *L* of the elementary circuits. Current versions of Johnson’s algorithm allow such a cutoff. In particular, the algorithm of Gupta & Suzumura^33^ for sparse graphs, with running time $O\left(\left(c+\left|V\right|\right)L{\mathrm{d̅}}^{L}\right)$ where *d̅* is the average degree, is attractive for applications to CRNs. So far, there is no comparably efficient approach to produce elementary circuits ordered by size. A related algorithm to enumerate chordless elementary circuits, optionally restricted to length *L*, is described in ref 69. We show that this is indeed feasible in a follow-up paper.^55^

All autocatalytic cores derive from induced fluffles, and more broadly from (not necessarily maximal) induced strong blocks in **K**. A recent linear-delay algorithm for enumerating strongly connected induced subgraphs^70,71^ may thus serve as an alternative starting point for the efficient generation of candidate subsets for autocatalytic cores.

As expected, the computational examples in the previous section identified a large number of irreducible autocatalytic subnetworks in metabolic systems. It remains an open question what fraction of those is of biological relevance. Clearly, this will depend on the metabolic fluxes that can potentially be realized given specific food sets.^18,21^ The enumeration of irreducible autocatalytic subnetworks at least makes it possible to address such questions computationally in systematic studies.
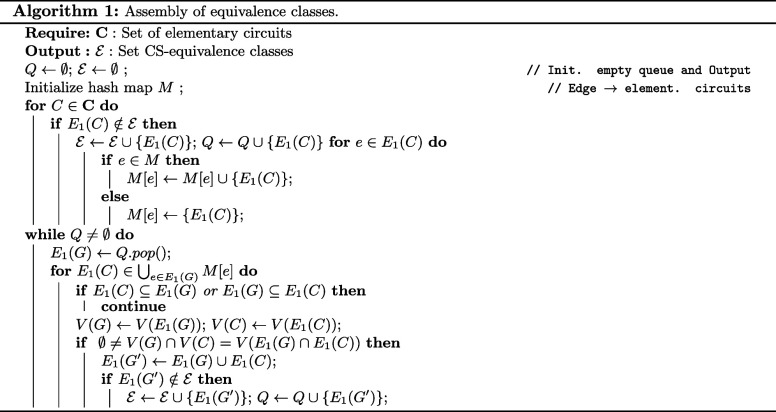


## Supplementary Material



## Data Availability

The implementation, models, and all necessary data is available at https://github.com/hollyritch/autogato.

## Footnotes

1
*Fluffle* is an informal, whimsical term for a group of rabbits, motivated by the rabbit’s ears in our context.

## Appendix

### Centralized Autocatalysis

Upon analyzing autocatalytic structures one is bound to notice a qualitative, dichotomic difference between two types. Consider the following two examples:

example I	example II
*x*_1_ → *x*_2_ + *x*_3_	*x*_1_ → *x*_2_ + *x*_3_
*x*_2_ → *x*_1_	*x*_2_ → *x*_1_ + *x*_3_
*x*_3_ → *x*_1_	*x*_3_ → *x*_1_ + *x*_2_

with associated CS matrices, respectively

$$\left(\begin{array}{ccc} -1 & 1 & 1\\ 1 & -1 & 0\\ 1 & 0 & -1 \end{array})\;(\begin{array}{ccc} -1 & 1 & 1\\ 1 & -1 & 1\\ 1 & 1 & -1 \end{array}\right)$$
19

In Example I, species *x*_1_ produces *x*_2_ and *x*_3_, and both in turn react back to *x*_1_. The autocatalytic process is thus centralized around *x*_1_: every reaction cycle passes through *x*_1_, so that autocatalysis can be interpreted as an amplification mechanism for *x*_1_. In contrast, in Example II, no single species plays such a role, due to a stronger interconnection of the network: there is a clear symmetry among indices 1, 2, 3, and the network topology is invariant under permutations of these labels. To capture and formalize this intuitive difference, we introduce the notion of centralized autocatalysis. Our first formulation is based on permutation cycles of the associated CS matrix. Subsequently, we will provide an equivalent graph-theoretic characterization. Recall the standard Leibniz formula for the determinant of a *k* × *k* CS matrix **S**[**κ**]­

20
$$\det S\left[\kappa \right]=\sum_{\pi \in P_{k}}sgn\left(\pi \right)\prod_{m=1}^{k}S{\left[\kappa \right]}_{m,\pi \left(m\right)}$$

where *P*_*k*_ denotes the symmetric group on *k* elements. Every non-identity permutation π ≠ id can be decomposed as a product of disjoint cyclic permutations of at least two elements

21
$$\pi =C_{1}\cdot ...\cdot C_{n_{\pi }}$$

We write $C_{\pi }$ for the set of permutation cycles of π. For simplicity of notation, we consider each cycle *C*_*i*_ as an element itself of *P*_*k*_, i.e., as a permutation of its *k* = |*X*_κ_| elements. We say that a permutation cycle C contributes (to det **S**[**κ**]) if

22
$$\prod_{m=1}^{k}S{\left[\kappa \right]}_{m,C\left(m\right)}\neq 0$$

We denote with  [κ] the set of non-trivial permutation cycles contributing to det **S**[**κ**], i.e. with length ≥2. In turn, permutation cycles without a contribution in the Leibniz formula [Disp-formula eq20] can be ignored in the following. We are now in the position to formalize the distinction between the two examples above:

**Definition 52**. Let **S**[**κ**] be a *k* × *k* irreducible autocatalytic Metzler CS matrix and denote by M_κ_ *the set of all m** ∈ {1, ..., *k*} such that every permutation cycle *C* contributing to det **S**[**κ**] satisfies $S{\left[\kappa \right]}_{m^{*},C\left(m^{*}\right)}>0$ . Then **S**[**κ**] is centralized if *M*_κ_ ≠ Ø . The elements of M_κ_ are the autocatalytic centers of **S**[**κ**] and we say that **S**[**κ**] is centered at *M*_κ_ provided *M*_κ_ ≠ Ø .

As a direct consequence of the definition, moreover, for centralized autocatalysis, the determinant computed via the Leibniz formula [Disp-formula eq20] naturally provides the sum over the weights of the different cycles in the network, as the next proposition states.

**Proposition 53** (Proof: Supporting Information). *Let* **S**[**κ**] be a *k* × *k* irreducible autocatalytic Metzler CS matrix that exhibits centralized autocatalysis. Then

23
$$\frac{\det S\left[\kappa \right]}{\prod_{m=1}^{k}S{\left[\kappa \right]}_{mm}}=1-\sum_{C}\prod_{m\in C}\frac{S{\left[\kappa \right]}_{m,C\left(m\right)}}{\left|S{\left[\kappa \right]}_{mm}\right|}$$

where the sum runs on all permutation cycles.

In essence, Prop. 53 differs from the Leibniz formula [Disp-formula eq20] because the sum runs over permutation cycles, only, instead over all permutations. Prop. 53 holds for centralized autocatalysis. Nevertheless, equality (23) does not solely apply to centralized autocatalysis as stated in Thm. 58. Hence, it does not provide a characterization of centralized autocatalysis.

Although examples of stoichiometric coefficients different from (0,1) do exist in metabolic networks, they are very rare. A well-known example is the condensation of two acetyl-CoA molecules into acetoacetyl-CoA in the synthesis of HMG-CoA during cholesterol or isopentenyl pyrophosphate (IPP) biosynthesis.^72^ In the special case of unit stoichiometric coefficients, Prop. 53 simplifies to an easily interpretable statement regarding the *number* of contributing permutation cycles of the stoichiometric matrix.

**Corollary 54**. Let **S**[**κ**] be a *k* × *k* irreducible autocatalytic Metzler CS matrix that exhibits centralized autocatalysis, and such that

$$S{\left[\kappa \right]}_{ij}\in \left\{-1,0,1\right\},forall\left(i,j\right)$$

Then

$$\det S\left[\kappa \right]{\left(-1\right)}^{k}=1-{\#}_{C}$$

where #_*C*_ is the number of permutation cycles *C* such that $\prod_{m=1}^{k}S{\left[\kappa \right]}_{m,C\left(m\right)}\neq 0$ .

Proof. It directly follows from Prop. 53.

□

For a *k*-CS **κ** = (*X*_κ_, *R*_κ_, κ), centralized autocatalysis can be characterized in graph-theoretical terms using a correspondence between contributing permutation cycles in its CS matrix **S**[**κ**] and directed elementary circuits in the induced subgraphs **K**[**κ**] ≔ **K**[*X*_κ_ ∪ *R*_κ_] of the König graph of the CRN. The key observation is that if *x* and *y* are consecutive vertices in a permutation cycle *C* that contributes to **S**[**κ**], then (*x*, κ­(*x*), *y*) is a path in **K**[**κ**] and, vice versa, if (*x*, *r*, *y*) is a path in **K**[**κ**] of an irreducible autocatalytic child-selection, then *r* = κ­(*x*). Denoting the elementary circuits (viewed as subgraphs of **K**[**κ**]) by **C**(**κ**), we obtain the following formal statement:

**Lemma 55** (Proof: Supporting Information). Let **S**[**κ**] be an autocatalytic Metzler CS matrix and  [κ] the set of contributing permutation cycles of length ≥2. Then there is a one-to-one correspondence between  [κ] and **C**(**κ**) such that a contributing permutation cycle (*x*_1_, *x*_2_, ...*x*_*k*_) corresponds to the elementary circuit (*x*_1_, κ­(*x*_1_), *x*_2_, κ­(*x*_2_), ..., κ­(*x*_*k*–1_), *x*_*k*_, κ­(*x*_*k*_), *x*_1_) *in* **K**[**κ**].

As a direct consequence of Lemma 55, we can now rephrase Def. 52 in graph-theoretical terms:

**Corollary 56**. *Let* **A** ≔ **S**[**κ**] *be* an autocatalytic Metzler CS matrix. Then **A** is centralized if and only if there is a vertex *x** ∈ *X*_κ_ such that *x** ∈ *X*(*C*) for all *C* ∈ **C**(**κ**).

We collect all center species *x** in a set *X*_κ_^*^ and refer to it as the autocatalytic center of **A**. Note that *x** and *X*_κ_^*^ correspond to *m** and *M*_κ_ above.

To simplify the notation, we introduce a normalized version of the matrix **A** by setting *N*(**A**)_*ij*_ ≔ **A**_*ij*_/|**A**_*jj*_| for all *i*, *j*. With this notation, Prop. 53 and Lemma 55 immediately imply.

**Corollary 57**. Let $A\in {\left\{Z\right\}}^{k\times k}$ be centralized in *X*_κ_^*^, denote by **C**_*x* ^*^_(**κ**) the set of cycles containing *x**, and let *s*_C_(*y*) be the successor of y along the elementary circuit *C.* Then

24
$$\det\left(A\right)\cdot {\left(-1\right)}^{k-1}=\sum_{C\in C_{x^{*}}\left(\kappa \right)}\prod_{y\in X\left(C\right)}\left|N{\left(A\right)}_{y,s_{C}\left(y\right)}\right|$$

for all autocatalytic centers *x* ^*^ ∈ *X*_κ_^*^. In particular if **A** ∈ {−1,0,1}^*k*^^×^^*k*^ then

25
$$\det\left(A\right)\cdot {\left(-1\right)}^{k-1}=\left|C_{x^{*}}\left(\kappa \right)\right|-1$$

We conclude this section by connecting the concept of centralized autocatalysis with the classification of autocatalytic cores proposed by Blokhuis et al.^16^ Their five types, in essence, correspond to the following five motifs (up to different stoichiometric coefficients)

26
$$TypeI:x_{1}\to x_{2}\to 2x_{1},\left(\begin{array}{cc} -1 & 2\\ 1 & -1 \end{array}\right)$$

27
$${TypeII}^{*}:\{\begin{array}{l} x_{1}\to x_{2}+x_{3}\\ x_{2}\to x_{3}\\ x_{3}\to x_{1} \end{array},\left(\begin{array}{ccc} -1 & 0 & 1\\ 1 & -1 & 0\\ 1 & 1 & -1 \end{array}\right)$$

28
$$TypeIII:\{\begin{array}{l} x_{1}\to x_{2}+x_{3}\\ x_{2}\to x_{1}\\ x_{3}\to x_{1} \end{array},\left(\begin{array}{ccc} -1 & 1 & 1\\ 1 & -1 & 0\\ 1 & 0 & -1 \end{array}\right)$$

29
$$TypeIV:\{\begin{array}{l} x_{1}\to x_{2}+x_{3}\\ x_{2}\to x_{1}+x_{3}\\ x_{3}\to x_{1} \end{array},\left(\begin{array}{ccc} -1 & 1 & 1\\ 1 & -1 & 0\\ 1 & 1 & -1 \end{array}\right)$$

30
$$TypeV:\{\begin{array}{l} x_{1}\to x_{2}+x_{3}\\ x_{2}\to x_{1}+x_{3}\\ x_{3}\to x_{1}+x_{2} \end{array},\left(\begin{array}{ccc} -1 & 1 & 1\\ 1 & -1 & 1\\ 1 & 1 & -1 \end{array}\right)$$

Examples 1 and 2 above are of Type III and Type V, respectively. For Type II, the classification of Blokhuis et al.^16^ allows for a more general structure with additional internal cycles. In what follows, we consider only the simplest Type II* motif above and refer to the original paper for a description of the general case. The next result states which of such motifs are centralized and establishes the range of validity of Eq. 23a.

**Theorem 58** (Proof: Supporting Information). An autocatalytic core of type I, II*, III, or IV is centralized. Moreover, eq 23 holds for all five types of cores.

We underline that Type II, in its more general form, is typically not centralized due to the presence of additional internal cycles. Similarly, eq 23 does not need to hold in this case. For brevity, we omit the straightforward demonstration of this observation, which lies outside the scope of this work.

### Algorithmic Considerations

To test whether an autocatalytic Metzler matrix **S**[**κ**] on (*X*_κ_, *R*_κ_) exhibits centralized autocatalysis, counting of elementary circuits passing each *x* ∈ *X*_κ_ is required. Since κ: *X*_κ_ → *R*_κ_ is bijective, there is a 1–1 correspondence between circuits in the induced subgraph **K**[**κ**] and the graph with vertex set *X*_κ_ and edges (*x*, *y*) whenever **S**[κ]_*yx*_ > 0. It therefore suffices to enumerate the set C of elementary circuits in **K**[**κ**], e.g., using Johnson’s algorithm and to store the vertices *V*(*C*) ∩ *X*_κ_ in a bit vector ζ_*C*_ for each C∈C . The component-wise conjunction of these vectors

31
$$\zeta^{*}≔\underset{C\in C}{\wedge }\zeta_{C}$$

identifies the set of autocatalytic centers as *M* = {*x* | *ζ*_*x*_^*^ = 1}. The autocatalytic Metzler matrix **S**[**κ**] is therefore centralized if and only if there is an *x* ∈ *X*_κ_ : *ζ*_*x*_^*^ ≠ 0.

### Minimal Autocatalytic Systems (MAS)

The ILP approach of^18^ is based on subnetworks (*X*′, *R*′) of a CRN (*X*, *R*) where *R*′ ⊆ *R* and *X*′ ≔ *X*(*R*′) is defined as the set of species participating in the set of reactions *R*′ either as reactant or as product (or both). From the perspective of the present paper, any child-selection (CS) **κ** = (*X*_κ_, *R*_κ_, κ) uniquely defines a subnetwork (*X*(*R*_κ_), *R*_κ_) as above, where typically *X*_κ_ ⊂ *X*(*R*′). The converse is, however, not true: the same subnetwork (*X*(*R*′), *R*′) may support another child-selection ***κ* ~**, i.e. with $R_{\mathrm{\kappa ̃}}=R_{\kappa }$ . Thus, different child-selections may be supported by the same subnetwork (*X*(*R*′), *R*′). Moreover, ***κ* ~** need not be defined on the same set of species, i.e. we may have $X_{\mathrm{\kappa ̃}}\neq X_{\kappa }$ as long as $X_{\mathrm{\kappa ̃}}\subseteq X\left(R_{\mathrm{\kappa ̃}}\right)=X\left(R_{\kappa }\right)$ . The key concept in the work of Gagrani et al.^18^ is introduced by Def. 3.2 (III.2 in the arXiv preprint) as follows: “A minimal autocatalytic subnetwork (MAS) is defined to be the subnetwork with the least number of reactions containing a particular autocatalytic core.” We rephrase this statement here as follows:

**Definition 59** (Def. 3.2 ^18^). A minimal autocatalytic subnetwork (MAS) is a subnetwork (*X*(*R*′), *R*′) containing an autocatalytic core that is induced by an inclusion-minimal set of reactions with this property.

Inclusion minimality of the reaction set *R*′ implies that an MAS does not contain more reactions than specified by the child-selection of its defining autocatalytic core, i.e. *R*′ = *R*_κ_ for a child-selection **κ** for which **S**[**κ**] is an autocatalytic core. Since Def. 59 imposes minimality only on the set of reactions, not all autocatalytic cores are associated with an MAS. In particular, an autocatalytic core **S**[**κ**_2_] with reaction set *R*_κ_2__ is not associated with an MAS if *R*_κ_2__ strictly contains a reaction set *R*_κ_1__ ⊂ *R*_κ_2__ that supports another autocatalytic core **S**[**κ**_1_]. By minimality of cores, **S**[**κ**_1_] necessarily involves a set *X*_κ_1__ of reactants that is not contained in *X*_κ_2__ = κ_2_^–1^(*R*_κ_2__). To see that such cases indeed exist, consider the following simple reaction network

32
$$\{\begin{array}{ll} x_{1}+x_{3} & \underset{1}{\to }x_{2}+x_{4}\\ x_{2} & \underset{2}{\to }2x_{1}+x_{3}\\ x_{4} & \underset{3}{\to }x_{3}, \end{array}$$

with stoichiometric matrix

33
$$S=\left(\begin{array}{ccc} -1 & 2 & 0\\ 1 & -1 & 0\\ -1 & 1 & 1\\ 1 & 0 & -1 \end{array}\right)$$

The first two columns and first two rows, corresponding to the CS κ_1_ = ({*x*_1_, *x*_2_}, {1, 2}, κ­(*x*_1_, *x*_2_) = (1, 2)), form the autocatalytic core

34
$$S\left[\kappa_{1}\right]=\left(\begin{array}{cc} -1 & 2\\ 1 & -1 \end{array}\right)$$

The MAS-oriented ILP implementation of Gagrani et al.^18^ therefore discards any reaction set strictly containing {1, 2}. However, by considering all three columns and rows 2, 3, and 4, we obtain another autocatalytic core

35
$$S\left[\kappa_{2}\right]=\left(\begin{array}{ccc} -1 & 1 & 0\\ 1 & -1 & 1\\ 0 & 1 & -1 \end{array}\right)$$

associated with the CS

$$\kappa_{2}=\left(\left\{x_{2},x_{3},x_{4}\right\},\left\{1,2,3\right\},\kappa \left(x_{2},x_{3},x_{4}\right)=\left(2,1,3\right)\right)$$

where *X*_κ_1__ = {*x*_1_, *x*_2_} ⊄ {*x*_2_, *x*_3_, *x*_4_}. Indeed, the ILP of Gagrani et al. does not detect the autocatalytic core **S**[**κ**_2_]. The two missing autocatalytic cores in the *E. coli* core metabolism can also be explained in this manner.

We note, finally, that it is a simple task to determine the set of all MAS in a given CRN if all autocatalytic cores have been computed. By considering the sets of reactions associated to all autocatalytic cores, it suffices to remove the sets that are non-minimal with respect to the inclusion relation. The graph-theoretic approach thus can also be used to enumerate MAS in addition to autocatalytic cores.

## Notes

Background: Graphs and Matrices (Basic Notation, Graph Theoretical Constructions). Proofs of Statements in the Main Text. The Set System of Circuitnets of Fluffles ((strong) accessibility, commutability, no confluence). Additional Computational Data: Information on the metabolic models analyzed in this contribution for *E. coli* (core metabolism), *E. coli* (DH5α), *human erythrocytes*, and *Methanosarcina barkeri*. Examples: Example for a type III autocatalytic core that does not admit a single elementary-circuit CS representative, Example for an autocatalytic core type IV in the pentose-phosphate-pathway (PPP) of the *E. coli* core model, Algorithmic Overview - Pseudocode and proof of correctness for the following algorithms: *Partitioning*, *Enumeration of elementary circuits*, Detecting autocatalytic capacity. Figures: Figure S1: Depiction of a type V autocatalytic core, Figure S2: Counterexample that the set system of circuitnets of fluffles is confluent, Figure S3: Length distribution of elementary and fluffles and their corresponding equivalence classes in the *E. coli* core model, Figure S4: Depiction of the two autocatalytic cores identified by autogato but not by the ILP approach of Gagrani et al.^18^ in the *E. coli* core model, Figure S5: Schematic depiction of an autocatalytic core type IV and an example from the PPP of the *E. coli* core model, Figure S6: Illustration for the fusion of two vertices of the partitioning tree with four intersecting metabolites (PDF)
