# Supplementary material for: Enumeration of Autocatalytic Subsystems in Large Chemical Reaction Networks
Source: J Chem Theory Comput. 2026 May 4;22(10):4888–907. doi: 10.1021/acs.jctc.5c01979 (PMC13217572; doi:10.1021/acs.jctc.5c01979)
Supplement: Supplementary file 1 [file ct5c01979_si_001.pdf]

Enumeration of Autocatalytic Subsystems in Large Chemical Reaction Networks – Supplementary Information

Richard Golnik*[1,3], Thomas Gatter[1], Peter F. Stadler[1,2,3,4,5,6,7,8,9], and Nicola Vassena[1]

[1]Bioinformatics Group, Department of Computer Science, Leipzig University, D-04107, Leipzig, Germany
[2]Interdisciplinary Center for Bioinformatics, Leipzig University, D-04107, Leipzig, Germany
[3]Zuse School for Embedded and Composite Artificial Intelligence (SECAI)
[4]Center for Scalable Data Analytics and Artificial Intelligence, Leipzig University, D-04107, Leipzig, Germany
[5]Max Planck Institute for Mathematics in the Sciences, D-04103 Leipzig Germany
[6]Department of Theoretical Chemistry, University of Vienna, A-1090 Wien, Austria
[7]Center for non-coding RNA in Technology and Health, University of Copenhagen, DK-1870, Frederiksberg, Denmark
[8]Facultad de Ciencias, Universidad Nacional de Colombia, Bogotá, Colombia
[9]Santa Fe Institute, Santa Fe, NM 87501, USA

*Email: richard@bioinf.uni-leipzig.de

Background: Graphs and Matrices

The main text follows well-established textbook terminology and notation. For completeness, we briefly review the basic definitions and well-known facts about matchings and circuits in directed graphs.

Basic Notation

Graphs. We consider here directed graphs $G = (V, E)$ with vertex set V and edge set $E \subseteq V \times V$ without loops, i.e., $(v, v) \notin E$ for all $v \in V$. Where necessary, we write $V(G)$ and $E(G)$. Whenever there is a directed edge (u, v) from vertex u to v, we say that u is an in-neighbor of v, and v is an out-neighbor of u. The in-degree and out-degree of a vertex are the number of its in-neighbors and out-neighbors, respectively. H is a subgraph of G if H is graph, $V(H) \subseteq V(G)$, and $E(G) \subseteq E(H)$. The subgraph H is spanning if $V(H) = V(G)$ and induced if $u, v \in V(H)$ and $(u, v) \in E(G)$ implies $(u, v) \in E(H)$. The adjacency matrix $\mathbf{A}$ of G is the $V \times V$ matrix with entries $\mathbf{A}_{uv} = 1$ if $(u, v) \in E(G)$ and $\mathbf{A}_{uv} = 0$ otherwise.

A walk of length $h \geq 0$ in G is an alternating sequence $(v_0, e_1, v_1, \ldots, e_h, v_h)$ of vertices and edges such that $e_i = (v_{i-1}, v_i)$ for $1 \leq i \leq h$. A walk is closed if $v_0 = v_h$. It is a *path* if $i \neq j$ implies $v_i \neq v_j$ and thus also $e_i \neq e_j$. A closed walk is an *elementary circuit* if $i \neq j$ implies $v_i \neq v_j$ for $i, j \neq 0$, i.e., if $(v_1, e_2, \ldots, e_h, v_h)$ is a path. A graph is *strongly connected* if there is a path from u to v for $u, v \in V$. The underlying undirected graph is obtained from G by ignoring the direction of the edges. It is equivalent to the *symmetrized graphs* G_s obtained by setting $V(G_s) = V(G)$ and $(u, v), (v, u) \in E(G_s)$ whenever $(u, v) \in E(G)$. A graph G is *connected* if its underlying undirected graph is connected in the usual sense for undirected graphs, or equivalently, if its symmetrized graph G_s is strongly connected.

Hypergraphs. Chemical reaction networks can be represented as directed hypergraphs with vertex set X, representing the chemical species, and a set of directed hyperedges R denoting the reactions. A directed hyperedge (E^-, E^+) is a pair of non-empty subsets $E^-, E^+ \subseteq V$ denoting the reactants and products,

respectively. The *König representation* of a directed hypergraph (X, R) is the directed bipartite graph with vertex set $V = X \cupdot R$ and edge set E such that $(x, r) \in E$ if there is $r = (E^-, E^+) \in R$ such that $x \in E^-$, and $(r, x) \in E$ if there is $r = (E^-, E^+) \in R$ such that $x \in E^+$. Throughout, we denote the König graph of the CRN under consideration by $\mathbf{K}$.

Linear Algebra. Given a matrix A with rows and columns indexed by ordered sets N and M, respectively, we denote by $A[K, L]$ the submatrix with rows indexed by $K \subseteq N$ and columns $L \subseteq M$. When $|K| = |L|$ the determinant of $A[K, L]$ is called a *minor*. If A is a square matrix, i.e., $|N| = |M|$, a square submatrix $A[K, L]$ with $K = L$ is called *principal submatrix* and its determinant *principal minor*. Since only one set K is needed to define a principal submatrix, we refer to a principal submatrix as $A[K]$. A permutation matrix P of size n is a square matrix that has exactly one entry 1 in each row and each column, and 0 elsewhere. A square matrix is *irreducible* if there exists no permutation matrix P of size n such that PAP^{-1} is an upper triangular matrix. Considering corresponding row and column indices as vertices and introducing an edge (i, j) if $A_{ij} \neq 0$ yields a graph Γ that is strongly connected if and only if A is irreducible. A matrix $A \in \mathbb{C}^{n \times n}$ is *Hurwitz stable* if all of its eigenvalues have negative real part. It is called *Hurwitz unstable* if it possesses at least one eigenvalue with positive real part.

Graph Theoretical Constructions

Matchings. A *matching* in $G = (V, E)$ is a subset $M \subseteq E$ of edges such that each vertex is incident to at most one edge. The notion of matchings is the same in directed and undirected graphs, i.e., the direction of the edges does not play a role. A matching M is *perfect* if every vertex is incident with an edge in M. While the existence of a perfect matching can be verified in polynomial time (using any algorithm for computing a maximum matching), counting the number of perfect matchings is #P-complete. Enumeration can be achieved with constant amortized time[1]. In the applications below, we consider matchings on directed bipartite graphs. Writing the vertex partition of the bipartite graph as $V =: X \cupdot R$, we are interested only in matchings $M \subseteq E_1 := (X \times R) \cap E$. Clearly, this is equivalent to matchings in the subgraph $G_1 = (V, E_1)$.

Cycle Bases and Ear Decompositions in Digraphs. Recall that a digraph is *strongly connected* if every vertex is reachable from every other vertex by means of a directed path. Equivalently, G is (weakly) connected if the underlying undirected graph is connected, and every vertex $x \in V(G)$ is contained in an elementary circuit. The cycle space of digraph is usually defined over $\mathbb{Z}$ as the kernel of the directed incidence matrix $\mathbf{H}$ with entries $H_{xe} = -1$ if x is the tail of the edge e, $H_{xe} = +1$ if x is the head of e, and 0 otherwise. The (incidence vectors of the) elementary circuits define the extremal rays of the non-negative cone $\{z \mid \mathbf{H}z = 0, z_e \geq 0 \,\forall e \in E\}$[2–4]. Every strongly connected digraph has a basis of the cycle space that consists of elementary circuits only, see Thm. 9 of Berge et al.[5].

A *directed ear* in a digraph G is a directed path in which all internal vertices have in-degree 1 and out-degree 1, while the initial vertex has out-degree at least 2 and the terminal vertex has in-degree at least 2. An ear is called *open* if its initial and terminal vertices are distinct, and *closed* otherwise. A digraph G is strongly connected if it can be obtained from a single directed cycle by successively adding (open or closed) ears[2,3,6]. The digraph G is a *strong block* if it is strongly connected and has no cut vertices. Equivalently, any two vertices in G lie on some elementary circuit. Moreover, G is a strong block if and only if it can be constructed by means of an open ear decomposition[7]. For the ear decompositions, an *ear basis* is obtained by completing each ear (after it has been attached) to an elementary circuit by a directed path from the terminal to the initial vertex of the ear.

In the main text, we will use the following straightforward property of ear decompositions for which we could not find a convenient reference, and thus it is proved here:

Lemma 1. *Let G' be a subgraph of G and suppose both G' and G are strong blocks. Then G' can be extended to G by adding a sequence of open ears.*

Proof. Consider a vertex $x \in V(G) \setminus V(G')$. Since G is a strong block, there is a vertex $y \in V(G')$ and an elementary circuit C that contains both x and y. Let $u \in V(G')$ be the first predecessor of x on C and $v \in V(G')$ the first successor on C in G'. Then the path $P = (u, \ldots, x, \ldots, v)$ is an ear. Clearly $G'' = G' \cup P$ is again a strong block. Thus, all vertices in $V(G) \setminus V(G')$ can be added to $V(G')$ sequentially attaching ears. The resulting graph G^* is a spanning subgraph of G. Any missing edges have both endpoints in

$V(G^*) = V(G)$ and thus are ears. As an immediate consequence, any circuit basis of G' can be extended to a circuit basis of G by adding elementary circuits composed of an ear as described above and a directed path connecting its attachment vertices in the previously constructed subgraph. □

The same argument works for strongly connected graphs if open and closed ears are allowed.

Proofs of Statements in the Main Text

Proposition M12. Let $\boldsymbol{\kappa} = (X_\kappa, R_\kappa, \kappa)$ be a k-CS whose associated CS-matrix $\mathbf{S}[\boldsymbol{\kappa}]$ is reducible, Metzler, and autocatalytic. Then there exists a k'-CS $\boldsymbol{\kappa}' = (X_{\kappa'}, R_{\kappa'}, \kappa')$ with $X_{\kappa'} \subset X_\kappa$, $R_{\kappa'} \subset R_\kappa$, and $\kappa'(X_{\kappa'}) = \kappa(X_{\kappa'})$, such that its associated CS-matrix $\mathbf{S}[\boldsymbol{\kappa}']$ is an irreducible autocatalytic Metzler matrix.

Proof. Since $\mathbf{S}[\boldsymbol{\kappa}]$ is reducible, there exists a permutation matrix such that

$$P\mathbf{S}[\boldsymbol{\kappa}]P^{-1} = \begin{pmatrix} A & 0 \\ B & C \end{pmatrix} \tag{1}$$

with irreducible A. Let now $X_{\kappa'} \subset X_\kappa$ represent the species corresponding to the rows of A. Then the triple $\boldsymbol{\kappa}' := (X_{\kappa'}, R_{\kappa'} := \kappa(X_{\kappa'}), \kappa' := \kappa|_{X_{\kappa'}})$ is a CS satisfying $\kappa(X_{\kappa'}) = \kappa'(X_{\kappa'})$. Moreover, $\mathbf{S}[\boldsymbol{\kappa}'] := A$ has negative diagonal entries since $\mathbf{S}[\boldsymbol{\kappa}]$ has negative diagonal entries; a fact that is not changed upon simultaneous rearrangement of rows and columns. $\mathbf{S}[\boldsymbol{\kappa}]$ being a Metzler matrix implies that $\mathbf{S}[\boldsymbol{\kappa}']$ has only non-negative off-diagonal entries, thus $\mathbf{S}[\boldsymbol{\kappa}']$ is Metzler. An analogous argument can be made for $\mathbf{S}[\boldsymbol{\kappa}''] := C$.

In addition, $\mathbf{S}[\boldsymbol{\kappa}]$ being autocatalytic implies that there exists $v > 0 : \mathbf{S}[\boldsymbol{\kappa}]v > 0 \Rightarrow P\mathbf{S}[\boldsymbol{\kappa}]v > 0$. We let $w := Pv$, then $w > 0$:

$$0 < P\mathbf{S}[\boldsymbol{\kappa}]v = P\mathbf{S}[\boldsymbol{\kappa}]P^{-1}Pv = \begin{pmatrix} \mathbf{S}[\boldsymbol{\kappa}'] & 0 \\ B & \mathbf{S}[\boldsymbol{\kappa}''] \end{pmatrix} w = \begin{pmatrix} \mathbf{S}[\boldsymbol{\kappa}']w_1 \\ Bw_1 + \mathbf{S}[\boldsymbol{\kappa}'']w_2 \end{pmatrix} \tag{2}$$

Thus $\mathbf{S}[\boldsymbol{\kappa}']w_1 > 0$. Assume now there is a column in $\mathbf{S}[\boldsymbol{\kappa}']$ without a positive entry. Then one reaction of $\mathbf{S}[\boldsymbol{\kappa}']$ has no product. Hence, there exists a permutation matrix P' such that:

$$P'\mathbf{S}[\boldsymbol{\kappa}']P'^{-1} = \begin{pmatrix} A' & \vec{0} \\ C' & x \end{pmatrix} \tag{3}$$

with $x < 0$ and $\vec{0} \in 0^{(m-1)\times 1}$. However, this implies that $\mathbf{S}[\boldsymbol{\kappa}']$ is reducible, which is a contradiction. Thereby, $\mathbf{S}[\boldsymbol{\kappa}']$ is autocatalytic. □

Lemma M19. Let $\mathbf{K}' = (X' \cup R', E_1' \cup E_2')$ be a subgraph of $\mathbf{K}$ with reactant vertices X', reaction vertices R', and edges $E_1 \subseteq X' \times R'$ and $E_2' \subseteq R' \times X'$ such that

1. $|X'| = |R'|$;
2. every $x \in X'$ has out-degree 1 and every $x \in R'$ has in-degree 1.

Then $\mathbf{K}'$ is child-selective with $\kappa(x) = r$ for $(x, r) \in E_1'$.

Proof. Since $x \in X'$ has out-degree 1 and $\mathbf{K}'$ is bipartite, there is a unique $\kappa(x) \in R'$. Analogously, for every $r \in R'$ there is a unique $\mu(r) \in X'$ and we have $(x, \kappa(x)) \in E_1'$ for all $x \in X'$ as well as $(\mu(r), r) \in E_1'$ for all $r \in R'$. Thus we have $\kappa(\mu(r)) = r$ and $\mu(\kappa(x)) = x$, i.e., $\mu(\kappa)$ is the identity on X' and $\kappa(\mu)$ is the identity on R'. Hence κ is a bijection and $\boldsymbol{\kappa} = (X', R', \kappa)$. By construction, we have $\mathbf{K}' = \mathbf{K}(\boldsymbol{\kappa})$. □

Lemma M22. Let $\boldsymbol{\kappa} = (X_\kappa, E_\kappa, \kappa)$ be a CS. Then $\mathbf{K}(\boldsymbol{\kappa})$ is strongly connected if and only if $\mathfrak{M}(\mathbf{S}[\boldsymbol{\kappa}])$ is irreducible.

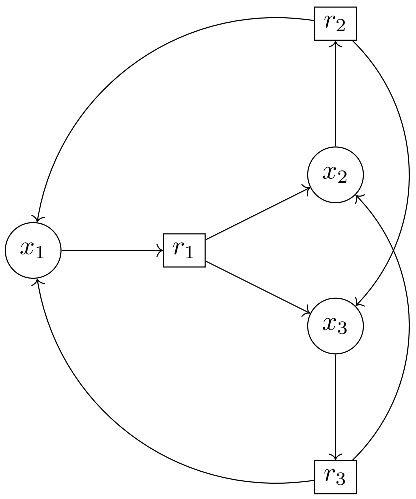

Figure S1: Depiction of a type V autocatalytic core

Proof. There is a path (x, r, y) in $\mathbf{K}(\boldsymbol{\kappa})$ if and only if $r = \kappa(x)$ and y is a product of r in X_κ, which is the case if and only if $\mathbf{S}_{y\kappa(x)} = \mathbf{S}[\boldsymbol{\kappa}]_{yx} > 0$, and hence if and only if $\mathbf{M}_{xy} := \mathfrak{M}(\mathbf{S}[\boldsymbol{\kappa}])_{xy} > 0$.
First suppose $\mathbf{K}(\boldsymbol{\kappa})$ is strongly connected. Then there is a path from x to y for all $x, y \in X_\kappa$, say $(x = z_0, r_1, z_1, \ldots, r_k, y = z_k)$ with $r_i = \kappa(z_{i-1})$. Thus $\mathbf{M}_{z_{i-1}z_i} > 0$ and hence $\mathbf{M}$ is irreducible. Conversely, suppose $\mathbf{M}$ is irreducible. Then for every pair $x, y \in X_\kappa$ there is a sequence of vertices $z_i \in X_\kappa$ with $x = z_0$ and $y = z_k$ such that $\mathbf{M}_{z_{i-1}z_i} > 0$ and hence $(x = z_0, \kappa(z_0), z_1, \kappa(z_1), \ldots, \kappa(z_{k-1}), y = z_k)$ is a path in $\mathbf{K}(\boldsymbol{\kappa})$. Moreover, for every reaction vertex r there is an edge (x, r) with $x = \kappa^{-1}(r)$ and an edge (r, y) since there is $y \in X_\kappa$ with $\mathbf{M}_{\kappa^{-1}(r),y} > 0$. Hence all vertices of $\mathbf{K}(\boldsymbol{\kappa})$ are reachable from each other. □

Lemma M23. If $\mathbf{K}(\boldsymbol{\kappa})$ is strongly connected, then it does not contain a cut vertex.

Proof. Indirectly assume that v is a cut vertex and $\mathbf{K}(\boldsymbol{\kappa})$ is strongly connected: this assumption implies that v has at least two in-edges and two out-edges. If v is reaction vertex, $v = r$, its in-edges in $\mathbf{K}(\boldsymbol{\kappa})$ are of the form $(\kappa^{-1}(r), r)$. Since κ is a bijection, there is at most one such edge, and thus we reach a contradiction. If v is in turn a substrate vertex, i.e., $v = x$, then all its out-edges are of the form $(x, \kappa(x))$, i.e., again, there is exactly one such edge, and thus we reach a contradiction analogously. Therefore, $\mathbf{K}(\boldsymbol{\kappa})$ cannot contain a cut vertex. □

Lemma M25. A CS $\boldsymbol{\kappa} = (X_\kappa, E_\kappa, \kappa)$ is autocatalytic if and only if the following conditions both hold:

1. there is a positive vector $v > 0$ such that $\mathbf{S}[\boldsymbol{\kappa}]v > 0$.
2. $\mathbf{K}(\boldsymbol{\kappa})$ does not possess source and sink vertices;

Proof. Property 1 is identical to property (i) of Def. M2.
First, suppose $\boldsymbol{\kappa}$ is autocatalytic. Property (i) of Def. M2 further implies that no substrate vertex is a source in $\mathbf{K}(\boldsymbol{\kappa})$ because the row $\mathbf{S}[\boldsymbol{\kappa}]_x$, corresponding to substrate x, satisfies $\mathbf{S}[\boldsymbol{\kappa}]_x v > 0$ and thus x is a product in at least one reaction. By Eq. (M14), this implies that x is not a source in $\mathbf{K}(\boldsymbol{\kappa})$. Property (ii) in Def. M2, on the other hand, implies that no reaction vertex is a sink. By Thm. M16, the CS bijection κ explicitly guarantees the existence of a perfect matching in the set of reactant-to-reaction edges in $\mathbf{K}(\boldsymbol{\kappa})$. Thus, no substrate vertex is a sink and no reaction vertex is a source $\mathbf{K}(\boldsymbol{\kappa})$. In summary, statement 2 of the lemma is satisfied.
Conversely, suppose conditions 1 and 2 hold. Since $\boldsymbol{\kappa}$ is a CS and there is no source or sink vertex in $\mathbf{K}(\boldsymbol{\kappa})$, then for every reaction vertex r, there is an edge (x, r) and an edge (r, y) and thus for every reaction r (that is, for every column) we have $\mathbf{S}[\boldsymbol{\kappa}]_{xr} < 0$ and $\mathbf{S}_{ry} > 0$, i.e., condition (ii) in Def. M2 is satisfied. Together with (i), $\mathbf{S}[\boldsymbol{\kappa}]$ is autocatalytic. □

Theorem M27. A graph G is a fluffle if and only if it is bipartite with vertex set $X \cup R$ and it has an ear decomposition such that every ear initiates in a reaction vertex $r \in R$ and terminates in a substrate vertex $x \in X$. In this case, all directed open ear decompositions have this property.

Proof. Let G be a fluffle. Then G is bipartite and a strong block. Thus, in particular, it has a directed open ear decomposition[7]. Now consider *any* decomposition $(P_1 = C, P_2, \dots, P_h)$, where $h \geq 1$ and P_1 is an elementary circuit and P_h, $h \neq 1$, is a path. As any ear decomposition starts from an elementary circuit C, it follows that for any substrate vertex $x \in V(C) \cap X$ of C, an out-neighbor of x is contained as well in $V(C)$. By condition (ii) in Prop. 26, such out-neighbors must be unique in the fluffle, and thus any substrate vertex $x \in V(C) \cap X$ cannot be an initial vertex of any ear. Respectively and in total analogy, a reaction vertex $r \in V(C) \cap R$ of the elementary circuit C cannot be a terminal vertex of an ear, since its only in-neighbor is also located along $V(C)$. Now let G_2 be the graph obtained by attaching the ear P_2 to C. Since G_2 is 2-connected, every substrate vertex $x \in V(G_2) \cap X$ already has a unique out-neighbor in $V(G_2)$ and every reaction vertex $r \in V(G_2) \cap R$ has a unique in-neighbor in $V(G_2)$. Thus, the next ear can only initiate at a vertex $r' \in V(G_2) \cap R$ and terminate at a vertex $x' \in V(G_2) \cap R$. Inductively, this argument holds true for all subsequent ears. Moreover, all ear decompositions are of this form.

Conversely, assume that G is bipartite with vertex partition $X \cup R$, let $(C, P_2, \dots, P_h)$ be an ear decomposition of G such that each P_i initiates in a reaction vertex $r \in V(G) \cap R$ and terminates in a substrate vertex $x \in V(G) \cap X$. Since G has an ear decomposition, then G is a strong block, i.e., G satisfies (iii) in Prop. 26. Moreover, as G is bipartite, the vertices along each ear P_i alternate between reaction vertices in R and substrate vertices in X, and since the first and last vertex of P_i belong to different sets we have $|V(P_i) \cap X| = |V(P_i) \cap R| = |V(P_i)|/2$. Writing $G_1 = C$ and G_i for the graph obtained by attaching the ear P_i to G_{i-1}, we have $|V(G_1) \cap X| = |V(G_1) \cap R| = |C|/2$ and $|V(G_i) \cap R| = |V(G_{i-1}) \cap R| + (|P_i|/2 - 1)$ as well as $|V(G_i) \cap X| = |V(G_{i-1}) \cap X| + (|P_i|/2 - 1)$, where the -1 accounts for the fact that initial and terminal vertices of P_i are already present in G_{i-1}. By induction, it follows immediately that $|V(G_i) \cap R| = |V(G_i) \cap X|$ for all i, and thus $|R(G)| = |X(G)|$, i.e., G satisfies (i). By construction, every substrate vertex $x \in X$ has a single out-neighbor r_x. We have $r_x \in V(C)$ if $x \in V(C)$ and $r_x \in V(P_i)$ if $x \in V(P_i)$. Similarly, every reaction vertex $r \in R$ has a single in-neighbor x_r satisfying $x_r \in V(C)$ if $r \in V(C)$ and $x_r \in V(P_i)$ if $r \in V(P_i)$. Thus G satisfies property (ii). Taking (iii), (i), and (ii) together, G is a fuffle. □

Lemma M28. Let G be a fluffle in $\mathbf{K}$ and G' a subgraph of G that is a strong block. Then G' is a fluffle.

Proof. Trivially, G' is bipartite, each substrate vertex $x \in X(G')$ has out-degree at most 1 and each reaction vertex $r \in R(G')$ has in-degree at most 1. Since G' is a strong block by assumption, it has no vertices with in-degree or out-degree 0, i.e., every $x \in X(G')$ has out-degree 1 and every $r \in R(G')$ has in-degree 1. Thus G' satisfied condition *(ii)*. Moreover, $|X(G')| = |R(G')|$ is satisfied because indirectly $|X(G')| < |R(G')|$ would imply that there is a reaction vertex $r \in R(G')$ without in-edge and $|X(G')| > |R(G')|$ would imply that there is a substrate vertex $x \in X(G')$ without out-edge, both leading to a contradiction to property *(ii)*, which we just proved. Hence G' also satisfies *(i)* and is a fluffle. □

Theorem M29. Let G be a fluffle with vertex partition $X \cup R$ and C an elementary circuit such that $\emptyset \subset G \cap C \subset C$. Then, the connected components of $G \cap C$ are directed paths P_i. Moreover, $G \cup C$ is a fluffle if and only if all such paths P_i start from a substrate vertex $x_i \in X$ and terminate with a reaction vertex $r_i \in R$.

Proof. Trivially, any connected component of proper subsets of an elementary circuit is a path whenever it starts and terminates with a vertex. In particular, then, the connected components P_i of the intersection $\emptyset \subset G \cap C \subset C$ are paths. Without loss of generality, we can arrange such paths P_i in circular order along C. Let then Q_i identifies the path in C that starts with the terminal vertex of P_{i-1} and terminates with the starting vertex of P_i (here the index i is to be intended in a cyclic group). Note that the Q_i are in a 1-to-1 relation with the connected components of the complement $C \setminus G$ of $C \cap G$ in C: they are obtained by adding to each connected component of $C \setminus G$ the starting and terminal vertices to obtain a path. Clearly, by construction, the paths Q_i are ears for G. Since G is a fluffle, then it admits itself an ear decomposition. Because the paths Q_i are vertex-disjoint, any arbitrary ear decomposition for G can then be extended to an ear decomposition for $G \cup C$ by adding in arbitrary order the paths Q_i. Theorem M27 therefore implies that

$G \cup C$ is a fluffle if and only if each ear Q_i initiates in a reaction vertex $r \in R$ and terminates in a substrate vertex $x \in X$. By complementary construction, this is equivalent to each path P_i initiating in a substrate vertex $x \in X$ and terminating in a reaction vertex $r \in R$. □

Lemma M34. Two circuitnets $\mathcal{C}_1$ and $\mathcal{C}_2$ for fluffles G_1 and G_2 yield the same CS matrix $\mathbf{S}[\boldsymbol{\kappa}]$ if and only if $\mathcal{C}_1 \doteq \mathcal{C}_2$.

Proof. Two circuitnets yield the same CS-matrix if and only if Eq. (M17) holds. That is, for the graphs $\bigcup(\mathcal{C}_1) = (X^{(1)} \cup R^{(1)}, E_1^{(1)} \cup E_2^{(1)})$, $\bigcup(\mathcal{C}_2) = (X^{(2)} \cup R^{(2)}, E_1^{(2)} \cup E_2^{(2)})$ it holds that $X^{(1)} = X^{(2)}$, $R^{(1)} = R^{(2)}$, $E_1^{(1)} = E_1^{(2)}$, and in particular $\mathcal{C}_1 \doteq \mathcal{C}_2$. In turn, since $\mathcal{C}_1$ and $\mathcal{C}_2$ are circuitnets for fluffles G_1 and G_2, then $E_1 = (x_i, r_j)$ is a perfect matching (Prop. M26 and Eq. (M14)) and thus fully specifies (X, R). □

Lemma M35. Let $\mathcal{C}_1$ and $\mathcal{C}_2$ be circuitnets for fluffles G_1 and G_2, respectively, and let C' and C'' be two elementary circuits. Assume $\mathcal{C}_1 \doteq \mathcal{C}_2$, $C' \doteq C''$ and $G'_1 := \bigcup(\mathcal{C}_1 \cup \{C'\})$ is a fluffle. Then $G'_2 := \bigcup(\mathcal{C}_2 \cup \{C''\})$ is a fluffle as well with $\mathcal{C}_1 \cup \{C'\} \doteq \mathcal{C}_2 \cup \{C''\}$.

Proof. First consider any fluffle $G = \bigcup(\mathcal{C})$ with circuitnet $\mathcal{C}$ and let C be an elementary circuit such that $G \cup C$ is a fluffle. By construction, we have $E_1(G \cup C) = E_1(G) \cup E_1(C)$. Moreover, $E(G) \cap E(C) \neq \emptyset$ since $G \cup C$ is fluffle, and hence a strong block. In particular, via Thm. M29, G and C share at least one directed path P initiating at a substrate-vertex and terminating at a reaction-vertex, i.e., $E_1(G) \cap E_1(C) \neq \emptyset$. Hence we have $E_1(G_1) \cup E_1(C') = E_1(G_1) \cup E_1(C'') = E_1(G_2) \cup E_1(C') = E_1(G_2) \cup E_1(C'')$ and $\emptyset \neq E_1(G_1) \cap E_1(C') = E_1(G_2) \cap E_1(C'')$, and thus Thm. M29 applies for G'_2, concluding that G'_2 is a fluffle. Def. M33 implies $\mathcal{C}_1 \cup \{C'\} \doteq \mathcal{C}_2 \cup \{C''\}$. □

Lemma M36. For every CS-equivalence class $[\mathcal{C}]$ there is a representative $\hat{\mathcal{C}}$ such that there exists a CS-equivalence class $[\mathcal{C}']$ with representative $\hat{\mathcal{C}}'$ and an elementary circuit C^* such that $\hat{\mathcal{C}}' \cup \{C^*\} \doteq \hat{\mathcal{C}}$ and $|V(\bigcup(\mathcal{C}'))| < |V(\bigcup(\mathcal{C}))|$.

Proof. The statement trivially holds for circuitnets that are single-elementary circuits. Let G be the fluffle associated with the circuitnet $\mathcal{C} = \{C_1, \dots, C_h\}$, $h > 1$, listed according to the ordering in Def. M30. Pick now the first circuitnet $\hat{\mathcal{C}} \subseteq \mathcal{C}$ for G such that any strict subset of $\hat{\mathcal{C}}$ is *not* anymore a circuitnet for G. Clearly, we can always find such a suitable candidate $\hat{\mathcal{C}}$ from any circuitnet $\mathcal{C}$ for G by iteratively removing the single elementary circuit C_i^* with the highest index i and checking whether the remaining set is a circuitnet for the very same fluffle G. Once $\hat{\mathcal{C}}$ is found, a further removal of the elementary circuit $\hat{C}_i^*$ with highest index i identifies a circuitnet $\hat{\mathcal{C}}' = \hat{\mathcal{C}} \setminus \hat{C}_i^*$ for $G' \subset G$ which, by Cor. M32, is itself a fluffle. Moreover, since $G' \subsetneq G$ and the removal of $\hat{C}_i^*$ removes an ear, $|V(G')| < |V(G)|$. □

Lemma M45. Let $\mathbf{K}(\boldsymbol{\kappa}^*)$ be a fluffle with irreducible autocatalytic Metzler CS matrix $\mathbf{S}[\boldsymbol{\kappa}^*]$ and let $\mathbf{K}(\boldsymbol{\kappa})$ be obtained from $\mathbf{K}(\boldsymbol{\kappa}^*)$ by adding a single ear with initial vertex in $R(\mathbf{K}(\boldsymbol{\kappa}^*))$, terminal vertex in $X(\mathbf{K}(\boldsymbol{\kappa}^*))$, and a non-empty set of internal vertices, together with all reaction-to-metabolite edges in $R(\mathbf{K}(\boldsymbol{\kappa})) \times X(\mathbf{K}(\boldsymbol{\kappa}))$. If $\mathbf{S}[\boldsymbol{\kappa}^*]$ is an autocatalytic CS matrix and $\mathbf{S}[\boldsymbol{\kappa}]$ is a Metzler matrix, then $\mathbf{S}[\boldsymbol{\kappa}]$ is an autocatalytic irreducible CS matrix.

Proof. Since $\mathbf{K}(\boldsymbol{\kappa}^*) = \mathbf{K}[\boldsymbol{\kappa}^*]$ is in particular a strong block, the addition of an ear makes the resulting graph G also a strong block, and thus a fluffle by Thm. M29 because the ear can be extended to an elementary circuit by any directed path in $\mathbf{K}(\boldsymbol{\kappa}^*)$ from its terminal to its initial vertex. Inserting the additional R-to-X edges does not affect the fluffle property, completing it to the corresponding representative CS-equivalence class, i.e., $\mathbf{K}(\boldsymbol{\kappa})$. Since $\mathbf{S}[\boldsymbol{\kappa}^*]$ is irreducible by assumption, $\mathfrak{M}(\mathbf{S}[\boldsymbol{\kappa}])$ is also irreducible.

Now suppose $\mathfrak{M}(\mathbf{S}[\boldsymbol{\kappa}])$ is a Metzler matrix and let $\mathbf{A}$ be the matrix obtained by renumbering the vertices such that the initial vertex of the ear is $k = |X(\mathbf{K}[\boldsymbol{\kappa}^*])|$, its terminal vertex is 1, and the substrate vertices

are ordered consecutively along the directed ear from $k+1$ to l. By construction $\mathbf{A}$ has the form

$$\begin{pmatrix}
\boxed{\mathbf{A}^*} & & & \cdots & & & \vec{g}_l \\
f_{k+1} & -a_{k+1} & & \cdots & & & \\
 & f_{k+2} & -a_{k+2} & \cdots & & & \\
 & & f_{k+3} & \ddots & & & \\
 & & & \ddots & -a_{l-2} & & \\
 & & & \cdots & f_{l-2} & -a_{l-1} & \\
 & & & \cdots & & f_{l-1} & -a_l
\end{pmatrix}$$

where the vector $\vec{g} \geq 0$ has a strictly positive first entry, and all a_i and f_i are strictly positive. Moreover, all entries that are left blank are non-negative, since $\mathbf{A}$, like $\mathbf{S}[\boldsymbol{\kappa}]$, is a Metzler matrix. Multiplying $\mathbf{A}$ with a strictly positive vector $\vec{u} = (\vec{u}^*, u_{k+1}, u_{k+2}, \ldots, u_l)^\top$ yields $\mathbf{A}\vec{u} = \vec{z} = (\vec{z}^*, z_{k+1}, z_{k+2}, \ldots, z_l)^\top$. Taking into account that the blank entries yield only non-negative contributions, we obtain component-wise inequalities for $\vec{z}$ from the terms that are shown explicitly:

$$\begin{aligned}
&\vec{z}^* \geq \mathbf{A}^*\vec{u}^* \\
&z_j \geq f_j u_{j-1} - a_j u_j \quad \text{for } k+1 \leq j \leq l
\end{aligned}$$

Hence $\mathbf{A}^*\vec{u}^* > 0$ implies $\vec{z}^* > 0$. The second set of inequalities implies $z_j > 0$ whenever $0 < u_j < (f_j/a_j) \cdot u_{j-1}$, for $k+1 \leq j \leq l$. With $u_k = u_k^*$ fixed, we can recursively choose $0 < u_j = (f_j/a_j) \cdot u_{j-1} - \epsilon_j$ with $\epsilon_j > 0$ small enough for $k+1 \leq j \leq l$. By induction from $j = k+1$ down to $j = l$, therefore there is always a positive choice of u_j that yields a positive entry z_j. In summary, therefore, if $\mathbf{A}^*\vec{u}^* > 0$, i.e., if $\mathbf{S}[\boldsymbol{\kappa}^*]$ is autocatalytic, then there is $\vec{u} > 0$ such that $\mathbf{A}\vec{u} > 0$, i.e., such that $\mathbf{S}[\boldsymbol{\kappa}]$ is autocatalytic. □

Theorem M46. Let $\mathbf{S}[\boldsymbol{\kappa}]$ be an irreducible Metzler CS matrix and suppose $\mathbf{S}[\boldsymbol{\kappa}]$ contains an autocatalytic core $\mathbf{S}[\boldsymbol{\kappa}^*]$ as a principal submatrix. Then $\mathbf{S}[\boldsymbol{\kappa}]$ is autocatalyic.

Proof. It suffices to recall that any fluffle can be obtained from a sub-fluffle by adding ears. Going from a fluffle to the canonical representative of its CS-equivalence class amounts to adding edges of the form (r, x), i.e., ears without internal vertices. Thus if $\mathbf{S}[\boldsymbol{\kappa}^*]$ is an autocatalytic core that is a principal submatrix of $\mathbf{S}[\boldsymbol{\kappa}]$ there is a sequence of ears, and thus a corresponding sequence of child-selections $\boldsymbol{\kappa}^* = \boldsymbol{\kappa}_0, \boldsymbol{\kappa}_1, \ldots, \boldsymbol{\kappa}_h = \boldsymbol{\kappa}$, such that $\mathbf{K}(\boldsymbol{\kappa}_i)$ is obtained from $\mathbf{K}(\boldsymbol{\kappa}_{i-1})$ by adding an ear with a non-empty set of interior vertices. Since $\mathbf{S}[\boldsymbol{\kappa}_h]$ is Metzler and all $\mathbf{S}[\boldsymbol{\kappa}_i]$, $0 \leq i \leq h$ are principal submatrices of $\mathbf{S}[\boldsymbol{\kappa}_h]$, each of the $\mathbf{S}[\boldsymbol{\kappa}_i]$ is an irreducible Metzler CS matrix. Applying Lemma M45 to each of the steps from $\mathbf{S}[\boldsymbol{\kappa}_{i-1}]$ to $\mathbf{S}[\boldsymbol{\kappa}_i]$ for $1 \leq i \leq h$ now implies that $\mathbf{S}[\boldsymbol{\kappa}_i]$ is autocatalytic whenever $\mathbf{S}[\boldsymbol{\kappa}_{i-1}]$ is autocatalytic. □

Lemma M49. Let G be a fluffle and C an elementary circuit. Then $G \cup C$ is a fluffle if and only if $\emptyset \neq V(G) \cap V(C) = V(E_1(G) \cap E_1(C))$.

Proof. Since fluffles are connected by definition, we may assume that $V(G) \cap V(C) \neq \emptyset$. We observe $B \coloneqq V(E_1(G) \cap E_1(C)) \subseteq V(E_1(G)) \cap V(E_1(C)) = V(G) \cap V(C) =: A$. First assume $A = B$. Thus $V(G) \cap V(C) \neq \emptyset$ implies that $E_1(G) \cap E_1(C) \neq \emptyset$ and thus $G \cup C$ is a strong block. Moreover, every edge in $E_1(C)$ is either contained in G or disjoint from G, and thus every maximal path in the intersection $G \cap C$ initiates with a metabolite $x \in X$ and terminates with a reaction $r \in R$. Thm. M29 now implies that $G \cup C$ is a fluffle. For the converse, assume that there is $z \in A \setminus B$. If $z \in X$, then there is a unique $y_1 \in V(G)$ with $(z, y_1) \in E_1(G)$ and $y_2 \in V(C)$ with $(z, y_2) \in E_1(C)$. We have $y_1 \neq y_2$ since otherwise $(z, y_1) = (z, y_2) \in E_1(G) \cap E(G)$. Thus $z \in X$ has out-degree 2 in $G \cup C$ and hence $G \cup C$ is not a fluffle. Similarly, if $z \in R$, there is $(y_1, z) \in E_1(G)$ and $(y_2, z) \in E_1(G)$ with $y_1 \neq y_2$ and thus z has in-degree 2 in $G \cup C$, which therefore is not a fluffle. □

Proposition M51. Let $\mathbf{S}[\boldsymbol{\kappa}]$ be a Hurwitz-stable autocatalytic CS-matrix. Then there exists a choice of parameters such that the system M(5): $\dot{z} = f(z) \coloneqq \mathbf{S} \cdot v(z)$ admits periodic solutions.

Proof. Blokhuis et al.[8] showed that any CS-matrix that is Hurwitz-stable but D-unstable admits a parameter choice for which M(5) has periodic solutions. Here, D-unstable means that there exists a positive diagonal matrix D such that $\mathbf{S}[\boldsymbol{\kappa}]D$ is Hurwitz-unstable. For simplicity, we present the case in which autocatalyticity of $\mathbf{S}[\boldsymbol{\kappa}]$ implies D-instability: namely, $\mathbf{S}[\boldsymbol{\kappa}]$ contains an autocatalytic core $\mathbf{A}[\kappa']$ as a principal submatrix, which is Hurwitz-unstable by Prop. M11. Without loss of generality, let $\mathbf{A}[\kappa']$ be the leading k'-dimensional principal submatrix of $\mathbf{S}[\boldsymbol{\kappa}]$, and define $D(\varepsilon) = \operatorname{diag} Po(1_1, \ldots, 1_{k'}, \varepsilon_{k'+1}, \ldots, \varepsilon_k)$. For $\varepsilon = 0$, $\mathbf{S}[\boldsymbol{\kappa}]D(0)$ is Hurwitz-unstable, as is $\mathbf{A}[\kappa']$. By continuity of eigenvalues, $\mathbf{S}[\boldsymbol{\kappa}]D(\varepsilon)$ remains Hurwitz-unstable for ε small enough, so $\mathbf{S}[\boldsymbol{\kappa}]$ is D-unstable. Stability together with D-instability implies the claim. A similar rescaling argument, which does not require D-instability, proves the result in full generality following Recipe 0 in Blokhuis et al.[8]. □

Proposition M53. Let $\mathbf{S}[\boldsymbol{\kappa}]$ be a $k \times k$ irreducible autocatalytic Metzler CS matrix that exhibits *centralized autocatalysis.* Then

$$\frac{\det \mathbf{S}[\boldsymbol{\kappa}]}{\prod_{m=1}^{k} \mathbf{S}[\boldsymbol{\kappa}]_{mm}} = 1 - \sum_{C} \prod_{m \in C} \frac{\mathbf{S}[\boldsymbol{\kappa}]_{m,C(m)}}{|\mathbf{S}[\boldsymbol{\kappa}]_{mm}|} \tag{4}$$

where the sum runs on all permutation cycles.

Proof. We recall the notation P_k for the permutation group on k elements. The first step is noting that if $\mathbf{S}[\boldsymbol{\kappa}]$ is centralized with center m^*, then for each permutation $\pi \in P_k$ with nonzero contribution, i.e. such that $\prod_{m=1}^{k} \mathbf{S}[\boldsymbol{\kappa}]_{m,\pi(m)} \neq 0$, we get that there exists exactly one permutation cycle C_π with $\pi = C_\pi$, i.e., any permutation with nonzero contribution is a single-cycle permutation. To confirm this, assume indirectly that there exists a permutation π with nonzero contribution and such that $\pi = C_1 \cdot \ldots \cdot C_i$, with $i \geq 2$. In particular, C_1 and C_2 have disjoint support and thus $C(m^*) \neq m^*$ cannot hold for both C_1 and C_2, which leads to a contradiction with the definition of centralized autocatalysis. The second step is just computing

$$\frac{\det \mathbf{S}[\boldsymbol{\kappa}]}{\prod_{m=1}^{k} \mathbf{S}[\boldsymbol{\kappa}]_{mm}}, \tag{5}$$

where the numerator is expanded via the Leibniz formula.

$$\begin{aligned}
\frac{\det \mathbf{S}[\boldsymbol{\kappa}]}{\prod_{m=1}^{k} \mathbf{S}[\boldsymbol{\kappa}]_{mm}} &= \frac{\sum_{\pi \in P_k} \operatorname{sgn}(\pi) \prod_{m=1}^{k} \mathbf{S}[\boldsymbol{\kappa}]_{m,\pi(m)}}{\prod_{m=1}^{k} \mathbf{S}[\boldsymbol{\kappa}]_{mm}} \\
&= \frac{\prod_{m=1}^{k} \mathbf{S}[\boldsymbol{\kappa}]_{mm}}{\prod_{m=1}^{k} \mathbf{S}[\boldsymbol{\kappa}]_{mm}} + \frac{\sum_{C} \operatorname{sgn}(C) \prod_{m \in C} \mathbf{S}[\boldsymbol{\kappa}]_{m,C(m)} \prod_{m \notin C} \mathbf{S}[\boldsymbol{\kappa}]_{mm}}{\prod_{m=1}^{k} \mathbf{S}[\boldsymbol{\kappa}]_{mm}} \\
&= 1 + \sum_{C} (-1)^{|C|-1} \prod_{m \in C} \frac{\mathbf{S}[\boldsymbol{\kappa}]_{m,C(m)}}{\mathbf{S}[\boldsymbol{\kappa}]_{mm}} \\
&= 1 - \sum_{C} \prod_{m \in C} \frac{\mathbf{S}[\boldsymbol{\kappa}]_{m,C(m)}}{|\mathbf{S}[\boldsymbol{\kappa}]_{mm}|}
\end{aligned} \tag{6}$$

□

Lemma M55. Let $\mathbf{S}[\boldsymbol{\kappa}]$ be an autocatalytic Metzler CS matrix and $\mathfrak{K}[\boldsymbol{\kappa}]$ the set of permutation cycles with non-zero contribution of length $\vartheta \geq 2$ (i.e., nontrivial cycles). Then there is a one-to-one correspondence between $\mathfrak{K}[\boldsymbol{\kappa}]$ and the elementary circuits of the induced subgraph $\mathbf{K}[\boldsymbol{\kappa}]$ such that a permutation cycle $(x_1, x_2, \ldots, x_\vartheta)$ corresponds to the elementary circuit $(x_1, \kappa(x_1), x_2, \kappa(x_2), \ldots, \kappa(x_{\vartheta-1}), x_\vartheta, \kappa(x_\vartheta), x_1)$ in $\mathbf{K}[\boldsymbol{\kappa}]$.

Proof. Let $\pi_c = (x_1, x_2, ..., x_\vartheta)$ be a nontrivial permutation cycle with nonzero contribution for the CS-matrix $\mathbf{S}[\boldsymbol{\kappa}]$, i.e., with index i following the labeling along the cycle,

$$\prod_{i=1}^{\vartheta} \mathbf{S}[\boldsymbol{\kappa}]_{\pi_c(i)i} \neq 0. \tag{7}$$

Since $\mathbf{S}[\boldsymbol{\kappa}]$ is Metzler, the nonzero off-diagonal entries are positive and correspond to products of the reaction with the respective column index. In particular, then, eq. (7) holds if and only if $\mathbf{S}[\boldsymbol{\kappa}]_{\pi_c(i)i} = s^+_{\pi_c(i)i} > 0$ for any $i = 1, ..., \vartheta$. Moreover, since $\mathbf{S}[\boldsymbol{\kappa}]$ is a CS-matrix, $\mathbf{S}[\boldsymbol{\kappa}]_{ii} = s^-_{i\kappa(i)} > 0$ for any $i = 1, ..., \vartheta$. These two observations are equivalent to the existence of edges in the induced subgraph $\mathbf{K}[\boldsymbol{\kappa}]$. Respectively, the existence of the edge $(x_i, \kappa(x_i))$ is equivalent to $s^-_{i\kappa(i)} > 0$ and the existence of the edge $(\kappa(x_i), x_{\pi_c(i)})$ is equivalent to $s^+_{\pi_c(i)i} > 0$. Following the index i along any contributing permutation cycle thus identifies one elementary circuit in the induced subgraph $\mathbf{K}[\boldsymbol{\kappa}]$. In turn, following the index i along any elementary circuit in the induced subgraph $\mathbf{K}[\boldsymbol{\kappa}]$ identifies one contributing nontrivial permutation cycle. The bijection follows. □

To see that the statement does not necessarily hold for a non-Metzler matrix, consider:

$$\mathbf{S}[\boldsymbol{\kappa}] = \begin{pmatrix} -1 & -1 \\ 1 & -1 \end{pmatrix} \tag{8}$$

which has one permutation cycle with nonzero contribution, i.e., (x_1, x_2), while the induced subgraph $\mathbf{K}[\boldsymbol{\kappa}]$ has no elementary circuit.

Theorem M58. An autocatalytic core of type I, II*, III, or IV is centralized. Moreover, Eq. (23), i.e.

$$\frac{\det \mathbf{S}[\boldsymbol{\kappa}]}{\prod_{m=1}^{k} \mathbf{S}[\boldsymbol{\kappa}]_{mm}} = 1 - \sum_{C} \prod_{m \in C} \frac{\mathbf{S}[\boldsymbol{\kappa}]_{m,C(m)}}{|\mathbf{S}[\boldsymbol{\kappa}]_{mm}|},$$

holds for all five types of cores.

Proof. We prove the theorem identifying the five autocatalytic cores exactly with the following five motifs and associated CS-matrices, respectively,

$$\textbf{Type I:} \qquad x_1 \underset{1}{\longrightarrow} x_2 \underset{2}{\longrightarrow} 2x_1 \qquad \begin{pmatrix} -1 & 2 \\ 1 & -1 \end{pmatrix} \tag{9}$$

$$\textbf{Type II}^*: \qquad \begin{cases} x_1 \underset{1}{\longrightarrow} x_2 + x_3 \\ x_2 \underset{2}{\longrightarrow} x_3 \\ x_3 \underset{3}{\longrightarrow} x_1 \end{cases} \qquad \begin{pmatrix} -1 & 0 & 1 \\ 1 & -1 & 0 \\ 1 & 1 & -1 \end{pmatrix} \tag{10}$$

$$\textbf{Type III:} \qquad \begin{cases} x_1 \underset{1}{\longrightarrow} x_2 + x_3 \\ x_2 \underset{2}{\longrightarrow} x_1 \\ x_3 \underset{3}{\longrightarrow} x_1 \end{cases} \qquad \begin{pmatrix} -1 & 1 & 1 \\ 1 & -1 & 0 \\ 1 & 0 & -1 \end{pmatrix} \tag{11}$$

$$\textbf{Type IV:} \qquad \begin{cases} x_1 \underset{1}{\longrightarrow} x_2 + x_3 \\ x_2 \underset{2}{\longrightarrow} x_1 + x_3 \\ x_3 \underset{3}{\longrightarrow} x_1 \end{cases} \qquad \begin{pmatrix} -1 & 1 & 1 \\ 1 & -1 & 0 \\ 1 & 1 & -1 \end{pmatrix} \tag{12}$$

$$\textbf{Type V:} \qquad \begin{cases} x_1 \underset{1}{\longrightarrow} x_2 + x_3 \\ x_2 \underset{2}{\longrightarrow} x_1 + x_3 \\ x_3 \underset{3}{\longrightarrow} x_1 + x_2 \end{cases} \qquad \begin{pmatrix} -1 & 1 & 1 \\ 1 & -1 & 1 \\ 1 & 1 & -1 \end{pmatrix}. \tag{13}$$

For a better visualization, we prove the theorem using the correspondence of elementary cycles in $\mathbf{K}[\boldsymbol{\kappa}]$ and the permutation cycles as cycles established in Lemma M55 above for type I-IV.
For type I, there is only one (permutation) cycle,

$$x_1 \to r_1 \to x_2 \to r_2 \to 2x_2, \tag{14}$$

and thus the autocatalytic core is centralized with centers both $\{x_1, x_2\}$.
For type II*, there are two (permutation) cycles:

$$\begin{cases} x_1 \to r_1 \to x_2 \to r_2 \to x_3 \to r_3 \to x_1; \\ x_1 \to r_1 \to x_3 \to r_3 \to x_1, \end{cases} \tag{15}$$

with $\{x_1, x_3\}$ being both centers.
For type III, there are two (permutation) cycles:

$$\begin{cases} x_1 \to r_1 \to x_2 \to r_2 \to x_1; \\ x_1 \to r_1 \to x_3 \to r_3 \to x_1, \end{cases} \tag{16}$$

with $\{x_1\}$ being a center.
For type IV, there are three (permutation) cycles:

$$\begin{cases} x_1 \to r_1 \to x_2 \to r_2 \to x_1; \\ x_1 \to r_1 \to x_3 \to r_3 \to x_1; \\ x_1 \to r_1 \to x_2 \to r_2 \to x_3 \to r_3 \to x_1, \end{cases} \tag{17}$$

with $\{x_1\}$ being a center.
For type V, there are five permutation cycles:

$$\begin{cases} x_1 \to r_1 \to x_2 \to r_2 \to x_1; \\ x_1 \to r_1 \to x_3 \to r_3 \to x_1; \\ x_2 \to r_2 \to x_3 \to r_3 \to x_2; \\ x_1 \to r_1 \to x_2 \to r_2 \to x_3 \to r_3 \to x_1; \\ x_1 \to r_1 \to x_3 \to r_3 \to x_2 \to r_2 \to x_1, \end{cases} \tag{18}$$

with no species being a center, see Fig. S1. One easily checks that those are also exactly the five elementary circuits in Fig. S1. Finally, an explicit and straightforward computation shows the validity of Eq. (23) for all five types. The straightforward generalization with different stoichiometric coefficients and by addition of monomolecular intermediates, e.g., by substituting $x_1 \to x_2$ with $x_1 \to I_1 \to \cdots \to I_n \to x_2$, is omitted for simplicity of presentation. For the latter, it suffices to say that any argument based on the number of (permutation) cycles is indeed insensitive to the addition of intermediates.

□

The Set System of Circuitnets of Fluffles

Here, we collect some properties of the set system $\mathfrak{F} \subseteq 2^{\mathcal{C}}$ of circuitnets whose union form fluffles. This is of interest because certain simple properties guarantee simple enumeration or the existence of efficient algorithms to find maximal elements. For our purposes, the following properties of the set system $(X, \mathfrak{A})$ with basis set X and $\mathfrak{A} \in 2^X$ are of interest:

(i) $(X, \mathfrak{A})$ is *accessible* if for all $A \in \mathfrak{A}, A \neq \emptyset$: there is $a \in A$ such that $A \setminus \{a\} \in \mathfrak{A}$.

(ii) $(X, \mathfrak{A})$ is *strongly accessible*[9] if it is accessible, and in addition, for any $A, B \in \mathfrak{A}$ with $A \subsetneq B$ there is $b \in B \setminus A$ such that $A \cup \{b\} \in \mathfrak{A}$.

(iii) A strongly accessible set system $(X, \mathfrak{A})$ is called *commutable*[10] if for any nonempty $A, B \in \mathfrak{A}$ and $a, b \in X : A \cup \{a\} \in \mathfrak{A}, A \cup \{b\} \in \mathfrak{A}$ and $A \cup \{a, b\} \subseteq B$ implies $A \cup \{a, b\} \in \mathfrak{A}$.

(iv) A commutable set system $(X, \mathfrak{A})$ is called *confluent* if for all $A, B, C \in \mathfrak{A}$ with $B \neq \emptyset$ that $B \subseteq A, B \subseteq C \Rightarrow A \cup C \in \mathfrak{A}$.

(v) $(X, \mathfrak{A})$ is an *independence system* or hereditary, if $A \in \mathfrak{A}$ and $\emptyset \neq B \subseteq A$ implies $B \in \mathfrak{A}$.

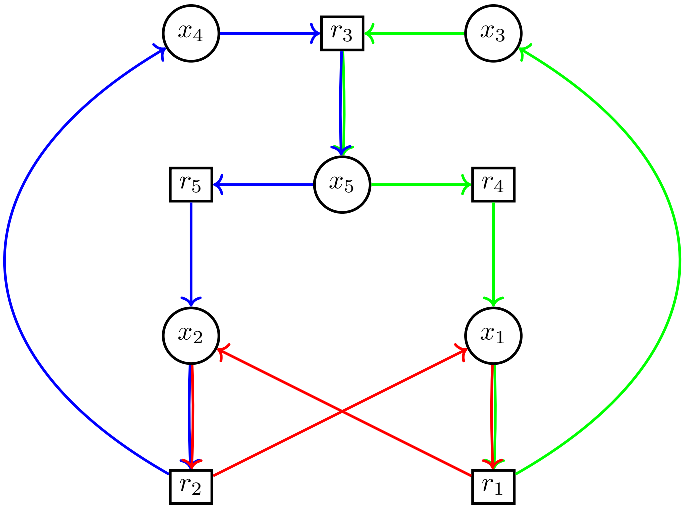

Figure S2: Counterexample to the hypothesis that $\mathfrak{F}$ is confluent. Consider the two child-selective elementary circuits $C_1 = (r_1, x_3, r_3, x_5, r_4, x_1, r_1)$ (green) and $C_2 = (r_2, x_4, r_3, x_5, r_5, x_2, r_2)$ (blue). Then $\{C_1, C_2\}$ is a circuitnet, but $\{C_1, C_2\} \notin \mathfrak{F}$ since $d_{in}(r_3) = 2$ or $d_{out}(x_5) = 2$. However, the circuit $C_3 = (x_1, r_1, x_2, r_2, x_1)$ (red) is a fluffle. In addition, $\{C_1, C_3\}, \{C_2, C_3\} \in \mathfrak{F}$, but $\{C_1, C_2, C_3\} \notin \mathfrak{F}$ since $\{C_1, C_2\} \notin \mathfrak{F}$.

Note that confluence is not comparable to the other properties.

Theorem 2. *$\mathfrak{F}$ is a commutable set system.*

Proof. If $\mathcal{C} \in \mathfrak{F}$ then there is a $C \in \mathcal{C}$ such that $\mathcal{C}' := \mathcal{C} \setminus \{C\}$ is a again a circuitnet. By Lemma M28, $\bigcup(\mathcal{C}')$ is again a fluffle, i.e., $\mathcal{C}' \in \mathfrak{F}$. That is, $\mathfrak{F}$ is accessible.

Now suppose $\mathcal{C}', \mathcal{C} \in \mathfrak{F}$ and $\mathcal{C}' \subsetneq \mathcal{C}$. Then there is a circuit $C_1 \in \mathcal{C}$ that shares an edge with a cycle $C_2 \in \mathcal{C} \setminus \mathcal{C}'$, since otherwise $\mathcal{C}$ cannot be a strong block. Thus $\mathcal{C}' \cup \{C_2\}$ is a strong block and Lemma M28 implies that $\mathcal{C}' \cup \{C_2\}$ is a fluffle. Since $\mathfrak{F}$ is accessible, it is also strongly accessible.

Let $\mathcal{C}, \mathcal{D} \in \mathfrak{F}$, $\mathcal{C} \subset \mathcal{D}$, $C_1, C_2 \in \mathcal{D} \setminus \mathcal{C}$, $\mathcal{C} \cup \{C_1\} \in \mathfrak{F}$, $\mathcal{C} \cup \{C_2\} \in \mathfrak{F}$, and $\mathcal{C} \cup \{C_1, C_2\} \subseteq \mathcal{D}$. Since $\mathcal{C} \neq \emptyset$, the union of the circuits in $\mathcal{C} \cup \{C_1\}$ and $\mathcal{C} \cup \{C_2\}$ are two strong blocks that share a strong block, namely the union of the circuits in $\mathcal{C}$. Thus $\mathcal{C}' := \mathcal{C} \cup \{C_1, C_2\}$ is also a strong block, and hence $\mathcal{C}'$ is a circuitnet. Since $\mathcal{C}' \subseteq \mathcal{D} \in \mathfrak{F}$, the union of the circuits is a fluffle by Lemma M28, and thus $\mathcal{C}' \in \mathfrak{F}$. Together with strong accessibility, this implies that $\mathfrak{F}$ is a commutable set system □

The example in Fig. S2 shows that the set system of fluffles $\mathfrak{F}$ is not confluent.

Instead of circuitnets, we use superpositions of the representatives of fluffle CS equivalence classes with the representatives of the CS equivalence classes of elementary circuits. The following statement follows immediately from Lemmas M34, M36, and Proposition M38:

Corollary 3. *Let $\mathcal{C} = \{C_1, \ldots, C_h\}$ be a circuitnet for a fluffle $G = \bigcup(\mathcal{C})$. Then the representative $[G]_{\doteq}$ of its CS-equivalence class is*

$$\left[\bigcup(\mathcal{C})\right]_{\doteq} = \bigcup_{i=1}[C_i]_{\doteq} \tag{19}$$

For every circuitnet $\mathcal{C} \in \mathfrak{F}$ we therefore can define a corresponding set of representatives $[\mathcal{C}]_{\doteq} := \{[C] \mid C \in \mathcal{C}\}$. Note that some of the representatives in $\mathcal{C}$ may be redundant. Now we consider the corresponding set system $[\mathfrak{F}] := \{[\mathcal{C}] \mid \mathcal{C} \in \mathfrak{F}\}$. As an immediate consequence of equ.(19), the arguments in the proof of Thm. 2 carry over to $[\mathfrak{F}]$, and thus we may conclude that

Corollary 4. *The set system of circuitnet representatives $[\mathfrak{F}]$ is commutable.*

This observation provides a formal basis for the stepwise enumeration on the system of fluffle representatives.

In principle one could also consider the set system $(E, \mathfrak{X})$ with $\mathfrak{X} := \{E_1(G)|G \text{ is fluffle in } \mathbf{K}\}$ on the edge set E of $\mathbf{K}$. It is easy to see, however, that $(E, \mathfrak{X})$ is not an accessible set system, since the deletion of each of the (r, x)-edges may lead to a subgraph that is not a strong block and thus also not a fluffle. Thus fluffles cannot be generated efficiently by exploring individual edge additions.

Additional Computational Data

***E. coli* core model** This CRN comprises 72 metabolites and 95 reactions [11]. We excluded the following set of small, highly connected molecules, which are of minor interest for autocatalysis: cytosolic NAD, NADH, NADP, NADPH, AMP, ADP, H^+, H_2O, CO_2, coenzyme A, phosphate, oxygen, ubiquinone, and ubiquinol. Since this model describes the central carbon metabolism of *E. coli*, we did not remove ATP as the major energy carrier.

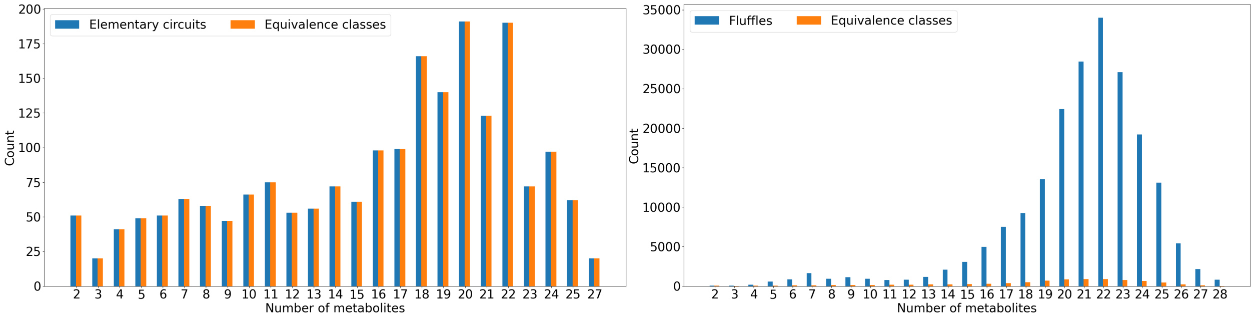

Figure S3: Length distribution of elementary circuits (left) and size distribution of fluffles and their CS-equivalance classes (right) for the *E. coli* core model.

Fig. 7 in the main text summarizes the distribution of elementary circuits and fluffles for the *E. coli* core network on a log scale. Here, we include the same data on a linear scale.

The comparison of our results with the ILP-formulation of Gagrani et al. [12] revealed two autocatalytic cores that were detected by our graph-theoretical algorithm only, see Fig. S4. Both are localized in the Pentose-Phosphate-Pathway.

Large *E. coli DH5*α5 model This CRN consists of 1,951 metabolites and 2,779 [13]. Compared to the core model, the list of molecules that were removed was augmented to allow for computational feasibility and meaningful results. For brevity, we only provide the species identifier. The assignment to the full names can be found at `http://bigg.ucsd.edu/models/iEC1368_DH5a/metabolites`. The list contains the metabolites from cytosol (c), periplasm (p), and extracellular space (e):
M_23camp_p, M_23ccmp_p, M_23cgmp_p, M_23cump_p, M_2fe1s_c, M_2fe2s_c, M_35cgmp_c, M_3amp_p, M_3cmp_p, M_3fe4s_c, M_3gmp_p, M_3ump_p, M_4fe4s_c, M_ACP_c, M_adp_c, M_alpp_p, M_amp_c, M_amp_p, M_apoACP_c, M_arbtn_e, M_arbtn_fe3_e, M_atp_c, M_btn_c, M_btnso_c, M_ca2_c, M_ca2_p, M_camp_c, M_cdp_c, M_cl_c, M_cmp_c, M_cmp_p, M_co2_c, M_co2_p, M_coa_c, M_colipa_e, M_cpgn_e, M_cpgn_un_e, M_ctp_c, M_cu2_c, M_cu2_p, M_cu_p, M_dadp_c, M_damp_c, M_damp_p, M_datp_c, M_dcamp_c, M_dcdp_c, M_dcmp_c, M_dcmp_p, M_dctp_c, M_dgdp_c, M_dgmp_c, M_dgmp_p, M_dgtp_c, M_didp_c, M_dimp_c, M_dimp_p, M_ditp_c, M_dnad_c, M_dsbaox_p, M_dsbard_p, M_dsbcox_p, M_dsbcrd_p, M_dsbdox_c, M_dsbdrd_c, M_dsbgox_p, M_dsbgrd_p, M_dtdp_c, M_dtmp_c, M_dtmp_p, M_dttp_c, M_dudp_c, M_dump_c, M_dump_p, M_dutp_c, M_enter_e, M_fad_c, M_fadh2_c', M_fe2_c, M_fe2_p, M_fe3_e, M_fe3hox_e, M_fe3hox_un_e, M_fecrm_e, M_fecrm_un_e, M_feenter_e, M_feoxam_e, M_feoxam_un_e, M_fmn_c, M_fmnh2_c, M_gdp_c, M_gdp_p, M_gmp_c, M_gmp_p, M_gtp_c, M_gtp_p, M_h2_c, M_h2_p, M_h2o2_c, M_h2o2_p, M_h2o_c, M_h2o_e, M_h2o_p, M_h2s_c, M_h_c, M_h_e, M_h_p, M_hacolipa_e, M_halipa_e, M_hco3_c, M_hdca_e, M_hqn_c, M_idp_c, M_imp_c, M_imp_p, M_itp_c, M_lipa_e, M_lipidA_e, M_lipidAp_e, M_metsox_R__L_e, M_metsox_S__L_e, M_mql8_c, M_mqn8_c, M_n2o_c, M_na1_c, M_na1_p, M_nad_c, M_nadh_c, M_nadp_c, M_nadph_c, M_nh4_c, M_nh4_p, M_nmn_c, M_nmn_p, M_no2_c,

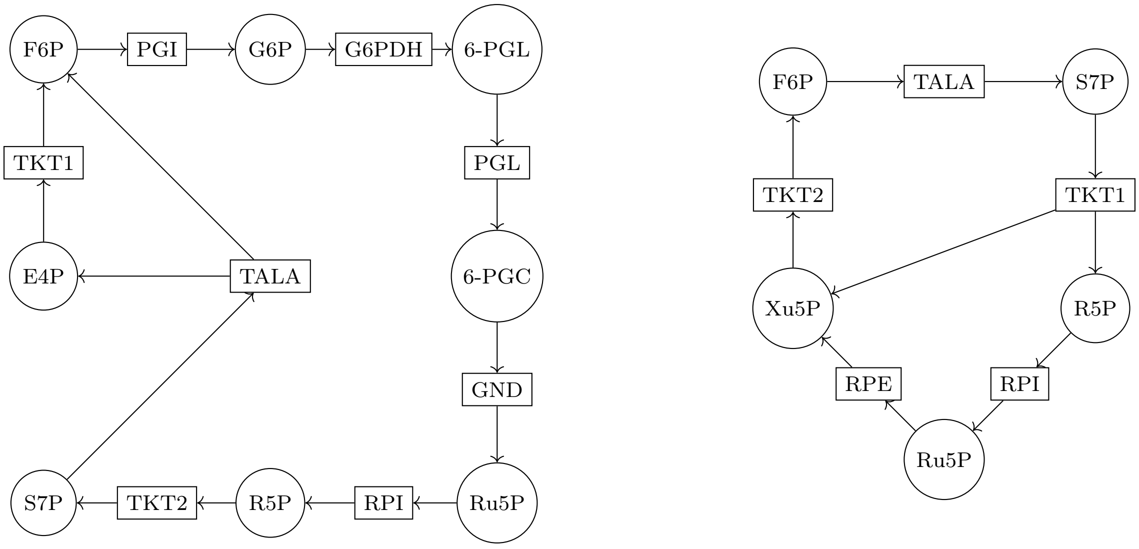

Figure S4: Two autocatalytic cores in the *E. coli* core model were identified by our graph-theoretical algorithm, but not by the ILP formulation of Gagrani et al.[12]

M_no2_p, M_no3_c, M_no3_p, M_no_c, M_o2_c, M_o2_p, M_o2s_c, M_o2s_p, M_pi_c, M_pi_p, M_ppi_c, M_pppi_c, M_q8_c, M_q8h2_c, M_rbflvrd_c, M_ribflv_c, M_s_c, M_sel_c, M_seln_c, M_slnt_c, M_so2_c, M_so3_c, M_so3_p, M_so4_c, M_thm_c, M_thmmp_c, M_thmpp_c, M_trnaala_c, M_trnaarg_c, M_trnaasn_c, M_trnaasp_c, M_trnacys_c, M_trnagln_c, M_trnaglu_c, M_trnagly_c, M_trnahis_c, M_trnaile_c, M_trnaleu_c, M_trnalys_c, M_trnamet_c, M_trnaphe_c, M_trnapro_c, M_trnasecys_c, M_trnaser_c, M_trnathr_c, M_trnatrp_c, M_trnatyr_c, M_trnaval_c, M_tsul_c, M_tsul_p, M_udp_c, M_ump_c, M_ump_p, M_utp_c.

***Homo sapiens* erythrocyte model** This CRN consists in total of 342 metabolites and 469[14]. It was constructed by taking advantage of the human RECON-1 metabolic model[15] and proteomic data from enucleated erythrocytes and covers two compartments only, the cytosol and the extracellular space. After removal of the below provided list of small molecules, 151 metabolites and 261 reactions in mainly two strongly connected components remained. Again, we only provide the species identifier. The assignment to the full names can be found at `http://bigg.ucsd.edu/models/iAB_RBC_283/metabolites`. The list contains the metabolites from the cytosol (c) and the extracellular space (e):
M_gdp_c, M_thmtp_c, M_nad_c, M_ump_c, M_arg__L_e, M_pi_c, M_3moxtyr_e, M_normete__L_e, M_cl_c, M_mal__L_e, M_spmd_e, M_gluala_e, M_thmpp_c, M_thm_e, M_imp_c, M_cdp_c, M_o2_c, M_band_c, M_utp_c, M_cl_e, M_dnad_c, M_35cgmp_c, M_hco3_c, M_dopa_e, M_adp_c, M_na1_e, M_h_c, M_coa_c, M_ptrc_e, M_cmp_c, M_ala__L_e, M_nadp_c, M_nadh_c, M_k_c, M_ppi_c, M_gmp_c, M_nh4_c, M_co_c, M_ctp_c, M_k_e, M_bandmt_c, M_na1_c, M_acnam_e, M_gtp_c, M_nmn_c, M_camp_c, M_udp_c, M_h2o_c, M_4pyrdx_e, M_mepi_e, M_h_e, M_ribflv_c, M_nrpphr_e, M_h2o2_c, M_nadph_c, M_ca2_c, M_fad_c, M_ncam_e, M_ca2_e, M_thmmp_c, M_thm_c, M_atp_c, M_amp_c, M_co2_c, M_pi_e, M_fmn_c, M_gly_e, M_fe2_c

***Methanosarcina Barkeri* model** This CRN consists of 628 metabolites and 690[14]. Again, we only provide the species identifier. The assignment to the full names can be found at `http://bigg.ucsd.edu/models/iAF692/metabolites`. The list contains metabolites from the cytosol (c) and the extracellular space (e):
M_f420_2_c, M_trnathr_c, M_h_c, M_h_e, M_f430p2_c, M_f420_3_c, M_imp_c, M_s_c, M_cu2_c, M_dctp_c, M_dtdp_c,M_trnaser_c, M_trnaarg_c, M_so3_e, M_trnaile_c, M_pppi_c, M_cobya_c, M_mma_e, M_ctp_c, M_ni2_c, M_dma_e, M_f420_1_c, M_ala__L_e, M_trnagly_c, M_dcdp_c, M_tma_e, M_nmn_c, M_itp_c, M_h2_c, M_cd2_e, M_btn_c, M_dcmp_c, M_dudp_c, M_no2_c,

M_cmp_c, M_tsul_c, M_dgtp_c, M_ch4_e, M_cobalt2_c, M_cbi_e, M_nh4_c, M_adp_c, M_n2_e, M_nad_c, M_f420_0_c, M_cd2_c, M_co2_c, M_dtmp_c, M_trnatrp_c, M_trnalys_c, M_camp_c, M_trnagln_c, M_ca2_c, M_k_e, M_h2s_c, M_f420_5_c, M_trnaasp_c, M_mg2_c, M_co_c, M_f420_4_c, M_pi_c, M_dttp_c, M_f390a_c, M_o2_c, M_f420_6_c, M_nadh_c, M_trnaala_c, M_ind3ac_e, M_dgdp_c, M_h2o_c, M_cdp_c, M_f390g_c, M_fe2_c, M_meoh_e, M_com_c, M_dms_e, M_o2s_c, M_f420_2h2_c, M_datp_c, M_cu2_e, M_cl_e, M_na1_e, M_hco3_c, M_so3_c, M_trnamet_c, M_pac_e, M_alac__S_e, M_co1dam_c, M_dadp_c, M_gtp_c, M_trnaval_c, M_coa_c, M_nadp_c, M_thmpp_c, M_ppi_c, M_f430p1_c, M_glyald_e, M_trnacys_c, M_fmn_c, M_thm_c, M_thmmp_c, M_f420_7_c, M_dcamp_c, M_na1_c, M_nadph_c, M_actn__R_e, M_atp_c, M_dutp_c, M_cob_c, M_co2dam_c, M_dnad_c, M_ump_c, M_cl_c, M_f430p3_c, M_damp_c, M_gmp_c, M_trnaglu_c, M_k_c, M_gdp_c, M_idp_c, M_s_e, M_trnaphe_c, M_f430_c, M_cbl1hbi_e, M_btn_e, M_ribflv_c, M_h2o2_c, M_udp_c, M_trnaleu_c, M_trnatyr_c, M_h2_e, M_trnahis_c, M_unknown_rbfdeg_e, M_amp_c, M_ca2_e, M_dump_c, M_unknown_cbl1deg_e, M_ch4s_e, M_utp_c, M_trnapro_c

Examples

Example 1 (Autocatalytic core of Type III does not admit an elementary-circuit CS-representative)**.** The CS-equivalence class of the autocatalytic cores of Types I, II, IV, and V contains a single circuit circuitnet; see the proof of Thm. M58 for a direct verification. In each of these cases, the list of elementary circuits includes at least one (two for Type V) circuit that passes through all species and reaction vertices. The only exception is Type III, shown in Fig. M5g. Here, the CS-matrix is

$$\mathbf{S}[\boldsymbol{\kappa}] = \begin{pmatrix} -1 & 1 & 1 \\ 1 & -1 & 0 \\ 1 & 0 & -1 \end{pmatrix}, \tag{20}$$

and it admits exactly two elementary circuits:

$$\begin{cases} x_1 \to r_1 \to x_2 \to r_2 \to x_1, \\ x_1 \to r_1 \to x_3 \to r_3 \to x_1, \end{cases} \tag{21}$$

neither of which traverses all vertices (both have length 2).

Example 2 (Autocatalytic core Type IV in the *E. Coli* core network)**.** Most autocatalytic cores described in the literature are of types I, II, or III. In the *E. coli* core network, we found a single example of a Type IV core in the pentose-phosphate-pathway (PPP). This example served as motivation for introducing the concept of centralized autocatalysis.

The Type IV autocatalytic core in Fig. S5 introduces one unit of G3P, which yields one unit of E4P and F6P each. E4P then generates one unit of G3P and one unit of F6P. Each of the two units of F6P finally produces a G3P, resulting in a gross yield of three G3P. There are indeed three elementary circuits in this network that contain G3P, while all other species are located on at most two elementary circuits. G3P therefore differs from the residual species and, since all elementary circuits coalesce in G3P, it forms the autocatalytic center.

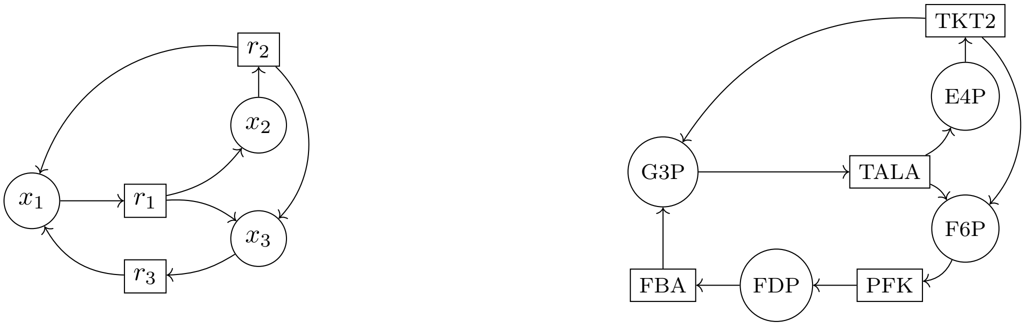

Figure S5: Example of an autocatalytic core of Type IV according to the classification of Blokhuis et al.[16] (left) and a topologically equivalent autocatalytic core detected in the pentose-phosphate-pathway (PPP) of the *E. coli* core network (right). Abbreviations of metabolites: G3P glyceraldehyde 3-phosphate; E4P erythrose 4-phosphate; F6P fructose 6-phosphate; FDP fructose 1,6-bisphosphatase. Reactions are labeled by the enzymes that catalyze them: TALA transaldolase A; TKT2 transketolase 2; PFK phosphofructokinase; FBA fructose-bisphosphate aldolase.

Algorithmic Overview

The algorithm constitutes five main parts, computing the set of all autocatalytic cycles and their properties from the König graph of a CRN:

- preparing the network
- decomposition of the network to biochemically functional units
- enumeration of elementary circuits
- enumeration of equivalence classes of fluffles
- classification of enumerated fluffles

The individual steps will be described in the following sections.

Algorithm S1: Algorithm overview

Require: $\mathbf{K}(X, R)$, set of small molecules to remove S
Output : $\mathcal{A}$ - Set of all autocatalytic Metzler matrices
$\mathcal{A} \leftarrow \emptyset$;
$\mathcal{A}_Z \leftarrow \emptyset$;
$\mathbf{K}(X, R) \leftarrow$ `RemoveSmallMolecules`$(S, \mathbf{K}(X, R))$; `// see Sec.` *Network Preparation*
for *SCC of* $\mathbf{K}(X, R)$ **do**
  $\mathbb{T} \leftarrow$ `PartitionNetwork`$(\mathbf{K}[SCC], (V, \leq))$; `// see Sec.` *Partitioning*
  $\mathfrak{Q} \leftarrow$ `EnumerateElementaryCircuits`$(\mathbb{T})$; `// see Sec.` *Enumeration of Elementary circuits*
  $\mathcal{E} \leftarrow$ `EquivalenceClassAssembly`$(\mathfrak{Q})$; `// see Alg. M1`
  $\mathcal{A} \leftarrow \mathcal{A} \cup$ `AutocatalyticActivity`$(\mathcal{E})$; `// see Sec.` *Autocatalytic capacity*
  $\mathcal{A}_Z \leftarrow$ `CheckCentrality`$(\mathcal{A})$; `// see Sec. M` *Centralized autocatalysis*

An overview of all components is given in Alg. S1. The enumeration of equivalence classes of fluffles is described in the main text in Alg. M1 and `CheckCentrality` follows Sec. M *Centralized autocatalysis.* The remaining components will be covered in the following sections.

Network Preparation

Highly interconnected metabolites that do not constitute a focal compound of a reaction, i.e., co-factors like ATP, NADH, etc., and small molecules such as CO_2 and H_2O, do not contribute to the generation of

chemically meaningful autocatalytic cycles. They are therefore removed to reduce complexity. We provide a manually curated list of small molecules for this purpose. Otherwise, the König graph of the input CRN is not modified and follows standard definitions.

Partitioning

Algorithm S2: PartitionNetwork

Require: $\mathbf{K}(X, R)$
Output : Partitioning tree $\mathbb{T}$
$\mathbb{T} \leftarrow (\mathbf{K}(X, R), \emptyset)$;
`PartitionNetworkRecursion`$(\mathbf{K}(X, R), \mathbb{T})$;

def `PartitionNetworkRecursion`*(*$\mathbf{K}(X, R)$*, shared* $\mathbb{T}$*)***:**
  $\mathcal{R} \leftarrow$ `GenerateReactionNetwork`$(\mathbf{K}(X, R))$;
  $ShReD \leftarrow$ `ComputeActualShReDMatrix`$(\mathcal{R})$;
  $P \leftarrow$ `ComputeExpectedShReDMatrix`$(\mathcal{R})$;
  $G \coloneqq P - ShReD$;
  $v \leftarrow$ `ComputeLeadingEigenvector`(G);
  $s \in \{-1, 1\}^n, s_i \coloneqq \begin{cases} -1 & \text{if } v_i \leq 0 \\ 1 & \text{else} \end{cases}$;
  if $v = 0$ **then**
    $s \in \{-1, 1\}^n, s_i \coloneqq \begin{cases} -1 & \text{if } v_i < 0 \\ 1 & \text{else} \end{cases}$;
  if $\sum_i \sum_j Q_{ij} s_i s_j \leq 0$ **then**
    return
  $\mathbf{K}(X_1, R_1), \mathbf{K}(X_2, R_2) \leftarrow$ `SplitNetwork`$(v, \mathbf{K}(X, R))$;
  if $\mathbf{K}(X_1, R_1)$ *or* $\mathbf{K}(X_2, R_2)$ *is a DAG* **then**
    return
  $V(\mathbb{T}) \leftarrow V(\mathbb{T}) \cup \mathbf{K}(X_1, R_1) \cup \mathbf{K}(X_2, R_2)$;
  $E(\mathbb{T}) \leftarrow E(\mathbb{T}) \cup (\mathbf{K}(X, R), \mathbf{K}(X_1, R_1))$;
  $E(\mathbb{T}) \leftarrow E(\mathbb{T}) \cup (\mathbf{K}(X, R), \mathbf{K}(X_2, R_2))$;
  `PartitionNetwork`$(\mathbf{K}(X_1, R_1), \mathbb{T})$;
  `PartitionNetwork`$(\mathbf{K}(X_2, R_2), \mathbb{T})$;

The partition algorithm takes the König graph of a CRN (without small molecules) as input and computes a partition tree $\mathbb{T}$ whose nodes are labeled by subnetworks in König representation. The resulting partition tree $\mathbb{T}$ is then used to determine interfaces between modules.

To ensure that all elements of $\mathcal{E}$ can be generated via linear combinations of elementary circuits, we treat strongly connected components S independently. For every $\mathbf{K}[S]$, a network $\mathcal{R} \coloneqq (R, E)$ with the reactions as vertices and $E \coloneqq \{(r_1, r_2) | \exists x \in X(S), s^+_{xr_1} > 0, s^-_{xr_2} > 0\}$ is generated. The next step partitions $\mathcal{R}$ based on a round trip distance metric, called *Shortest Retroactive Distance* (ShReD)[17]. The difference between the expected and actual *ShReD* matrices, i.e. $G \coloneqq P -$ ShReD, is employed to solve an integer linear programming (ILP) problem: $\max Q \coloneqq \sum_{i=1}^n \sum_{j=1}^n G_{ij} \cdot s_i \cdot s_j$, s.t. $s \in \{-1, 1\}^n$. By construction, G is symmetric. Thus all eigenvalues are real, and for the sake of reduced runtime, we take advantage of the fact that the leading eigenvector of G, i.e., the eigenvector to the largest eigenvalue, approximates the solution vector for the given optimization problem as proposed in[18]. More details can be found in[17]. Importantly, each partitioning step yields two submodules $\mathbf{K}(X_1, R_1), \mathbf{K}(X_2, R_2)$, where $R = R_1 \cup R_2, R_1 \cap R_2 = \emptyset$ and $X_i \coloneqq \{x \in X \mid \exists r \in R_i : s^-_{xr} > 0 \text{ or } s^+_{xr} > 0\}$. A partitioning tree $\mathbb{T}$ is constructed such that $(\mathbf{K}(X, R), \mathbf{K}(X_1, R_1)), (\mathbf{K}(X, R), \mathbf{K}(X_2, R_2)) \in E(\mathbb{T})$. The upper steps are repeated recursively until either $Q = 0$ or one of the submodules contained is a DAG.

For more details on the functions `GenerateReactionNetwork()`, `ComputeActualShReDMatrix()`, `Compute-ExpectedShReDMatrix()`, and `SplitNetwork()` we refer to the original publication of the implemented

partition algorithm[17].

Enumeration of Elementary circuits

The enumeration of elementary circuits follows the partition tree $\mathbb{T}$ of the last step from bottom to top. First, Johnson's algorithm[19] is applied to all leaf nodes, which ensures the detection of all elementary circuits within biochemical functional modules. Upon merging two modules, i.e., for interior nodes of $\mathbb{T}$, we restrict Johnson's algorithm to metabolites in the intersection that lie along directed paths from one child module into the another, see Fig. S6. It should be noted that while we expect that exhaustive enumeration of all circuits will always be possible, enumeration for joined partitions may become infeasible for larger networks, and is therefore size-limited in practice.

Algorithm S3: OrientedNetwork

```
Require: Root, OutNetwork, InNetwork, L
Output : G
G ← Root;
for u ∈ L do
    V(G) ← V(G) ∪ {u_in, u_out};
    for v ∈ V_in^G(u) do
        if v ∈ V(InNetwork) then
            E(G) ← E(G) ∪ {(v, u_in)};
        else if v ∈ V(OutNetwork) then
            E(G) ← E(G) ∪ {(v, u_out)};
    for v ∈ V_out^G(u) do
        if v ∈ V(InNetwork) then
            E(G) ← E(G) ∪ {(u_in, v)};
        else if v ∈ V(OutNetwork) then
            E(G) ← E(G) ∪ {(u_out, v)};
        V(G) ← V(G) \ {u};
return G
```

Theorem 5. *Let (X, R) be a CRN, $\mathfrak{Q}(\mathbf{K})$ be the set of all elementary circuits of its König graph, and $\mathcal{C}$ the set of elementary circuits generated via Algorithm S4. Then $\mathfrak{Q}(\mathbf{K}) = \mathcal{C}$.*

Proof. We consider each strongly connected component independently since there are no elementary circuits connecting two strongly connected components by definition. By construction, $\mathbb{T}$ is a strict binary tree, leading to a simple bottom enumeration scheme where a node is visited only after the full sub-trees of both children have been visited. Note that from a purely algorithmic perspective, nodes may be visited in arbitrary order, as circuit sets for each node are independent. However, we require an order of closed sets of nodes for proof by induction. Leaf nodes serve as the base case, representing the minimal sub-networks of the partition. Here, the enumeration of elementary circuits is achieved by Johnson's Algorithm[19,20], which has shown to be complete. In the inductive step, we only consider inner tree nodes; therefore, as the partition tree is strict, parent nodes $\mathbf{K}(X_\kappa, R_\kappa)$ (node) with non-empty children, $\mathbf{K}(X_1, R_1)$ (left) and $\mathbf{K}(X_2, R_2)$ (right).

We first note that by construction, $R_\kappa = R_1 \cup R_2$ and $X_\kappa = X_1 \cup X_2$. While $R_1 \cap R_2 = \emptyset$, $X_1 \cap X_2$ is not necessarily empty. Thus, for fusing two children, we consider only elementary circuits containing at least one intersecting metabolite. If $X_1 \cap X_2 = \emptyset$, there is nothing to do since there are no edges connecting $\mathbf{K}(X_1, R_1)$ and $\mathbf{K}(X_2, R_2)$. In any other case, we enumerate elementary circuits containing at least one compound of $X_1 \cap X_2$. Several algorithms have been proposed to enumerate circuits containing a fixed node, such as modifications of Johnsons' Algorithm[19], with available pre-existing implementations[21]. Simply applying Johnson on each node $y_i \in Y = X_1 \cap X_2$ yields a superset of desired circuits, as we also enumerate subsets of elementary circuits of $\mathbf{K}(X_1, R_1)$ and $\mathbf{K}(X_2, R_2)$, which have already been enumerated by induction. This is unproblematic from a purely mathematical standpoint, but would drastically increase runtime complexity.

Algorithm S4: EnumerateElementaryCircuits

```
Require: Partitioning tree 𝕋
Output : Elementary Circuits 𝔔
𝔔 ← ∅;
t ← root(𝕋);
EnumElemCircuitsRec(t, 𝔔);

def EnumElemCircuitsRec(tree node t ∈ V(𝕋), shared 𝔔):
    if t == Leaf then
        𝔔 ← 𝔔 ∪ Johnsons(t, {})
    else
        t1, t2 = children(t);
        EnumElemCircuitsRec(t1);
        EnumElemCircuitsRec(t2);
        Y ← X(t1) ∩ X(t2);
        L ← {};
        if Y ≠ ∅ then
            for y ∈ Y do
                LIRO ← OrientedNetwork(t, t1, t2, L);        // see Alg. S3, proof Thm. 5
                RILO ← OrientedNetwork(t, t2, t1, L);
                𝔔'1 ← Johnsons(LIRO, {y});
                𝔔'2 ← Johnsons(RILO, {y});
                𝔔1 ← Backtranslation(𝔔'1);                   // see proof Thm. 5
                𝔔2 ← Backtranslation(𝔔'2);
                𝔔 ← 𝔔 ∪ 𝔔1 ∪ 𝔔2;
                L ← L ∪ {y};
```

To avoid this duplicate enumeration, we need to additionally enforce the inclusion of reactions from both R_1 and R_2 in each circuit. We say a circuit crosses child borders, implying that an intersecting metabolite is a product for a reaction from the left child and a reactant for a reaction from the right child or vice versa. More formally, a border node $y_i \in Y \cap C$ of circuit C is product of a unique reaction $r_i^+ \in C$, connected by an ingoing edge $(r_i^+, y_i) \in E(C)$, and educt of unique reaction $r_i^- \in C$, connected by an outgoing edge $(y_i, r_i^-) \in E(C)$. If $r_i^+, r_i^- \in R_1$ or $r_i^+, r_i^- \in R_2$ for all $y_i \in Y \cap X(C)$, the circuit C includes no crossing and was therefore already enumerated by induction. Otherwise there is at least one $y_i \in Y \cap X(C)$ s.t. $r_i^+ \in R_1$ and $r_i^- \in R_2$ or vice versa. This leads to the following procedure (Fig. S6): For each vertex $y_i \in Y$ we consider two antidromic networks left-in-right-out (LIRO) and right-in-left-out (RILO), derived by removing all outgoing edges (respectively ingoing) in R_1 and all ingoing (respectively outgoing) in R_2 adjacent to y_i. Calling Johnson on y_i on both networks now yields exactly the set of desired circuits containing y_i. Therefore, in the i-th iteration, all elementary circuits containing y_i are enumerated crossing y_i from the left to the right child or from the right to the left child, avoiding exactly all circuits not crossing at y_i. Again, completeness of the Johnsons algorithm guarantees finding all circuits. However, completeness also implies that circuits crossing metabolites on multiple points $Y' \subseteq Y \cap X(C)$ will be enumerated exactly $|Y'|$ times without further algorithmic restrictions. Given a fixed node order in Y, on i-th iteration any circuit with $y_i \in Y'$ was already enumerated iff there exists a $y_k \in Y' : k < i$. To restrict re-enumeration, we split nodes $y_k \in Y' : k < i$ into novel nodes $y_{k(in)}$ and $y_{k(out)}$ s.t. $y_{k(in)}$ inherits edges in $\mathbf{K}(X_1, R_1)$ and $y_{k(out)}$ edges in $\mathbf{K}(X_2, R_2)$, thereby exactly disabling crossing, but not inclusion of previous metabolites. `Backtranslation` reverses this operation on the cycle node and edge sets. We note that elementary circuits containing both versions of an intersecting metabolite, $y_{i(in)}$ and $y_{i(out)}$, such as the blue circuit depicted in Fig. S6 (y_3), are removed by backtranslation. It follows that all elementary circuits for the parent, $\mathbf{K}(X_\kappa, R_\kappa)$, are exactly enumerated without duplication.

By induction, we conclude that all elementary circuits for $\mathbf{K}(X, R)$ are enumerated. □

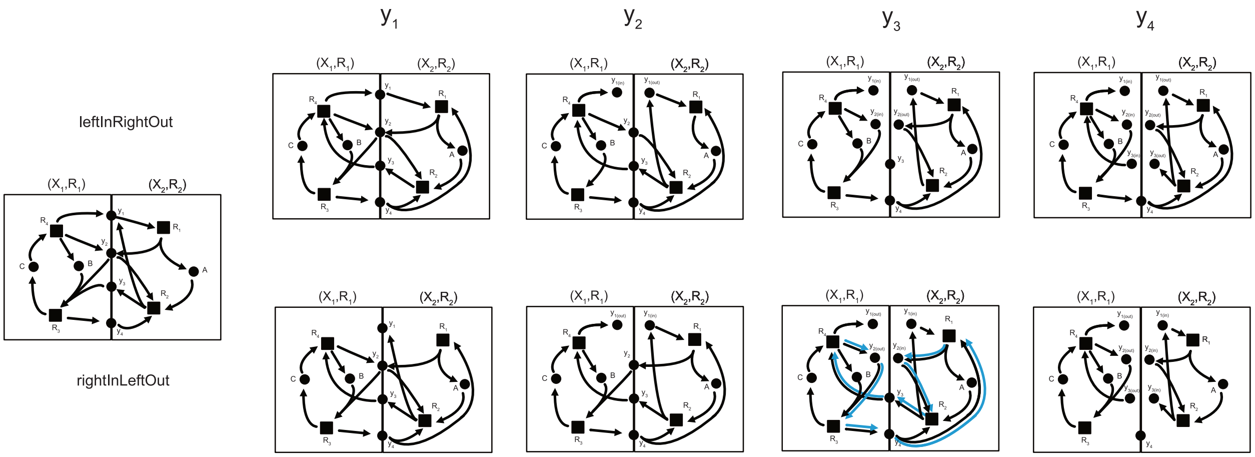

Figure S6: Depiction of an example for the fusion of two vertices of the partitioning tree $\mathbb{T}$ with four intersecting metabolites, i.e. $Y \coloneqq X_1 \cap X_2 = \{y_1, y_2, y_3, y_4\}$. Iterative steps for intersecting metabolites are depicted in the four right panels. The upper and lower panel are illustrating the two antidromic oriented networks, left-in-right-out (LIRO) and right-in-left-out (RILO), for each iteration of intersecting metabolites. In detail, in the i-th iteration and LIRO orientation (i-th upper panel), y_i exhibits incoming and outgoing edges only from the left and right child, respectively, while for RILO orientation, y_i receives only incoming and outgoing edges from the right and the left child, respectively. In the $i+1$-th iteration for both orientations, LIRO and RILO, y_i is split into $y_{i(in)}$ and $y_{i(out)}$. Subsequently, $y_{i(in)}$ is added to the left and $y_{i(out)}$ to the right network (LIRO) or vice-versa (RILO). In addition, edges pointing into y_i are strictly retained in the appurtenant oriented network, i.e., $y_{i(in)}$ is only incident to edges with origin or target in the in-oriented network. In the lower third panel, an elementary circuit is depicted in blue, which contains both versions of the original vertex. Nevertheless, after re-translation into the original vertex sequence, it is not an elementary circuit. Thus, it is not considered to be checked for its autocatalytic capacity or for the assembly of larger cycles.

Autocatalytic capacity

The autocatalytic capacity of a CS matrix can be determined for Metzler and non-Metzler matrices utilizing different methods. In the Metzler case, spectral properties are of great value, while for non-Metzler matrices, an optimization problem needs to be solved. Consider an $n \times n$ irreducible, Metzler matrix A. If A possesses any eigenvalue with a positive real part, in particular the leading one, then A is autocatalytic (see Lemma M11). For non-Metzler matrices, autocatalytic capacity is determined by the existence of a positive vector $v > 0$, s.t. $Av > 0$. This allows for a relatively straightforward implementation as it is shown in the pseudocode presented in Algorithm S5.

Algorithm S5: AutocatalyticCapacity

Require: $\mathcal{E}$, set of equivalence classes
Output : $\mathcal{A}$, set of autocatalytic matrices
$\mathcal{A} \leftarrow \emptyset$;
for $E_1(C) \in \mathcal{E}$ **do**
    $X(C), R(C) \leftarrow$ `SplitVertices`$(E_1(C))$;
    $n \leftarrow |X(C)|$;
    $A \leftarrow \mathbf{S}[X(C), \kappa_C(R(C))]$; `// Compute Matrix from Graph`
    if $A == \mathfrak{M}(A)$ **then** `// Case: Is Metzler Matrix`
        $r_{\max} \leftarrow \max\{\mathrm{Real}(\lambda) \mid \lambda \in \mathrm{spectrum}(A)\}$;
        if $r_{\max} > 0$ **then** `// If Hurwitz unstable`
            $\mathcal{A} \leftarrow \mathcal{A} \cup \{A\}$;
    else `// Case: Is non-Metzler Matrix`
        if $\exists v \in \mathbb{R}^{|n|}_{>0} : Av > 0$ **then**
            $\mathcal{A} \leftarrow \mathcal{A} \cup \{A\}$;

References

[1] Fink, J. Constant Time Enumeration of Perfect Bipartite Matchings. 2025; DOI: 10.48550/arxiv.2509.16135.

[2] Galluccio, A.; Loebl, M. (P,q)-Odd Digraphs. *Journal of Graph Theory* **1996**, *23 (2)*, 175–184.

[3] Loebl, M.; Matamala, M. Some Remarks on Cycles in Graphs and Digraphs. *Discrete Mathematics* **2001**, *233 (1-3)*, 175–182.

[4] Gleiss, P. M.; Leydold, J.; Stadler, P. F. Circuit Bases of Strongly Connected Digraphs. *Discussiones Mathematicae Graph Theory* **2003**, *23 (2)*, 241.

[5] Berge, C. *Graphs and Hypergraphs*; North-Holland Mathematical Library; North Holland: Amsterdam, 1973; Vol. 6.

[6] Havet, F.; Nisse, N. Constrained Ear Decompositions in Graphs and Digraphs. *Discrete Mathematics & Theoretical Computer Science* **2019**, *21 (4)*, 4544.

[7] Grötschel, M. On Minimal Strong Blocks. *Journal of Graph Theory* **1979**, *3 (3)*, 213–219.

[8] Blokhuis, A.; Stadler, P. F.; Vassena, N. Stoichiometric Recipes for Periodic Oscillations in Reaction Networks. 2025; DOI: 10.48550/arXiv.2508.15273.

[9] Boley, M.; Horváth, T.; Poigné, A.; Wrobel, S. Listing Closed Sets of Strongly Accessible Set Systems with Applications to Data Mining. *Theoretical Computer Science* **2010**, *411 (3)*, 691–700.

[10] Conte, A.; Grossi, R.; Marino, A.; Versari, L. Listing Maximal Subgraphs Satisfying Strongly Accessible Properties. *SIAM Journal on Discrete Mathematics* **2019**, *33 (2)*, 587–613.

[11] Orth, J. D.; Fleming, R. M. T.; Palsson, B. Ø. Reconstruction and Use of Microbial Metabolic Networks: The Core *Escherichia Coli* Metabolic Model as an Educational Guide. *EcoSal Plus* **2010**, *4 (1)*, 10.1128/ecosalplus.10.2.1.

[12] Gagrani, P.; Blanco, V.; Smith, E.; Baum, D. Polyhedral Geometry and Combinatorics of an Autocatalytic Ecosystem. *Journal of Mathematical Chemistry* **2024**, *62 (5)*, 1012–1078.

[13] Monk, J. M.; Koza, A.; Campodonico, M. A.; Machado, D.; Seoane, J. M.; Palsson, B. O.; Herrgård, M. J.; Feist, A. M. Multi-Omics Quantification of Species Variation of Escherichia Coli Links Molecular Features with Strain Phenotypes. *Cell Systems* **2016**, *3 (3)*, 238–251.e12.

[14] Bordbar, A.; Jamshidi, N.; Palsson, B. O. iAB-RBC-283: A Proteomically Derived Knowledge-Base of Erythrocyte Metabolism That Can Be Used to Simulate Its Physiological and Patho-Physiological States. *BMC systems biology* **2011**, *5*, 110.

[15] Duarte, N. C.; Becker, S. A.; Jamshidi, N.; Thiele, I.; Mo, M. L.; Vo, T. D.; Srivas, R.; Palsson, B. Ø. Global Reconstruction of the Human Metabolic Network Based on Genomic and Bibliomic Data. *Proceedings of the National Academy of Sciences of the United States of America* **2007**, *104 (6)*, 1777–1782.

[16] Blokhuis, A.; Lacoste, D.; Nghe, P. Universal Motifs and the Diversity of Autocatalytic Systems. *Proceedings of the National Academy of Sciences* **2020**, *117 (41)*, 25230–25236.

[17] Sridharan, G. V.; Hassoun, S.; Lee, K. Identification of Biochemical Network Modules Based on Shortest Retroactive Distances. *PLoS Computational Biology* **2011**, *7 (11)*, e1002262.

[18] Newman, M. E. J. Modularity and Community Structure in Networks. *Proceedings of the National Academy of Sciences* **2006**, *103 (23)*, 8577–8582.

[19] Johnson, D. B. Finding All the Elementary Circuits of a Directed Graph. *SIAM Journal on Computing* **1975**, *4 (1)*, 77–84.

[20] Gupta, A.; Suzumura, T. Finding All Bounded-Length Simple Cycles in a Directed Graph. 2021; DOI: 10.48550/arXiv.2105.10094.

[21] Hagberg, A. A.; Schult, D. A.; Swart, P. J. Exploring Network Structure, Dynamics, and Function Using NetworkX. Proceedings of the 7th Python in Science Conference. Pasadena, CA USA, 2008; pp 11–15.
